# Allosterically Regulated Guest Binding Determines Framework Symmetry for an Fe^II^_4_L_4_ Cage

**DOI:** 10.1002/anie.202301319

**Published:** 2023-03-28

**Authors:** Weichao Xue, Kai Wu, Nianfeng Ouyang, Thierry Brotin, Jonathan R. Nitschke

**Affiliations:** ^1^ Yusuf Hamied Department of Chemistry University of Cambridge Cambridge CB2 1EW UK; ^2^ Laboratoire de chimie Université Lyon Ens de Lyon, CNRS UMR 5182 69342 Lyon France

**Keywords:** Allosteric Regulation, Host–Guest Chemistry, Metal-Organic Cages, Structural Adaptation, Symmetry

## Abstract

Self‐assembly of a flexible tritopic aniline and 3‐substituted 2‐formylpyridine subcomponents around iron(II) templates gave rise to a low‐spin Fe^II^_4_L_4_ capsule, whereas a high‐spin Fe^II^_3_L_2_ sandwich species formed when a sterically hindered 6‐methyl‐2‐formylpyridine was used. The Fe^II^_4_L_4_ cage adopted a new structure type with *S*_4_ symmetry, having two *mer*‐*Δ* and two *mer*‐*Ʌ* metal vertices, as confirmed by NMR and X‐ray crystallographic analysis. The flexibility of the face‐capping ligand endows the resulting Fe^II^_4_L_4_ framework with conformational plasticity, enabling it to adapt structurally from *S*_4_ to *T* or *C*_3_ symmetry upon guest binding. The cage also displayed negative allosteric cooperativity in simultaneously binding different guests within its cavity and at the apertures between its faces.

## Graphical abstract

Metal‐organic cages are an important class of synthetic hosts, which are able to mimic the structural adaptability and allosteric regulation of naturally occurring systems. A low‐symmetry Fe^II^_4_L_4_ tetrahedral cage reported here was able to adapt its geometry from *S*_4_ to *C*_3_ or *T* symmetry upon guest binding; multiple binding sites allowed the host to simultaneously bind distinct guests with negative allostery.

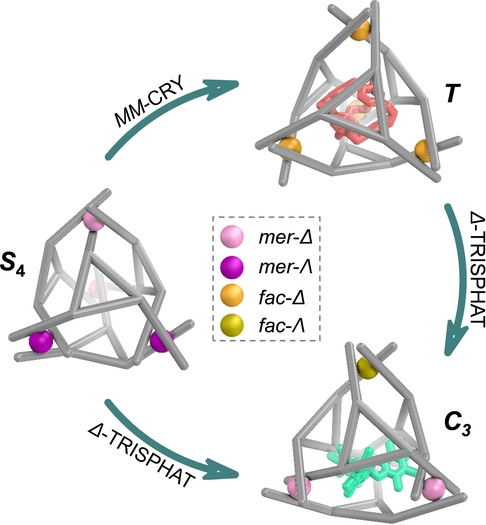

Biological receptors can dynamically adapt to optimize binding affinity, thus enhancing or inhibiting signal transduction in living systems.^[1]^ Allosteric regulation, whereby biological receptors transmit the effect of binding a substrate at one site to another at a distant site, is an essential process by which natural systems process information.^[2]^ The development of artificial allosteric systems, which are capable of emulating the biologically occurring processes of activity regulation, can enable new applications,^[3]^ but these systems are challenging to design.^[4]^

Metal‐organic cages formed by coordination‐driven self‐assembly, with well‐defined cavities and binding sites, have emerged as a powerful platform for the modular design of biomimetic supramolecular systems. These cages have also proven useful across diverse areas, including molecular recognition and sensing,^[5]^ chemical separation,^[6]^ stabilization of otherwise unstable species,^[7]^ and catalytic transformations.^[8]^ The reversible linkages between metal vertices and coordination sites can enable these cages to disassemble and reassemble in response to external stimuli,^[9]^ for instance, temperature,^[10]^ light,^[11]^ redox,^[12]^ and pH.^[13]^

Most cages contain symmetric and rigid ligands, which produce high‐symmetry structures resembling Platonic and Archimedean solids, such as tetrahedra,^[14]^ octahedra,^[15]^ cubes,^[16]^ cuboctahedra^[17]^ and other higher‐order structures.^[18]^ The high symmetry of such synthetic hosts differentiates them from biological systems, as natural receptors are rarely isotropic, highly symmetric species.^[19]^ To this end, the rational design of low‐symmetry host systems with inherent conformational adaptability is needed, in order to study intricate binding behaviors in adaptable chemical systems, which may led to the development of bioinspired applications.

Here we present the synthesis of a *S*_4_‐symmetric Fe^II^_4_L_4_ cage **1** by subcomponent self‐assembly (Figure 1). This cage dynamically adapted upon guest encapsulation to form host–guest complexes with either lower (*C*_3_) or higher (*T*) symmetry. The cage also simultaneously bound different guests at two distinct sites, with negative allosteric modulation observed between them.

**Figure 1:**
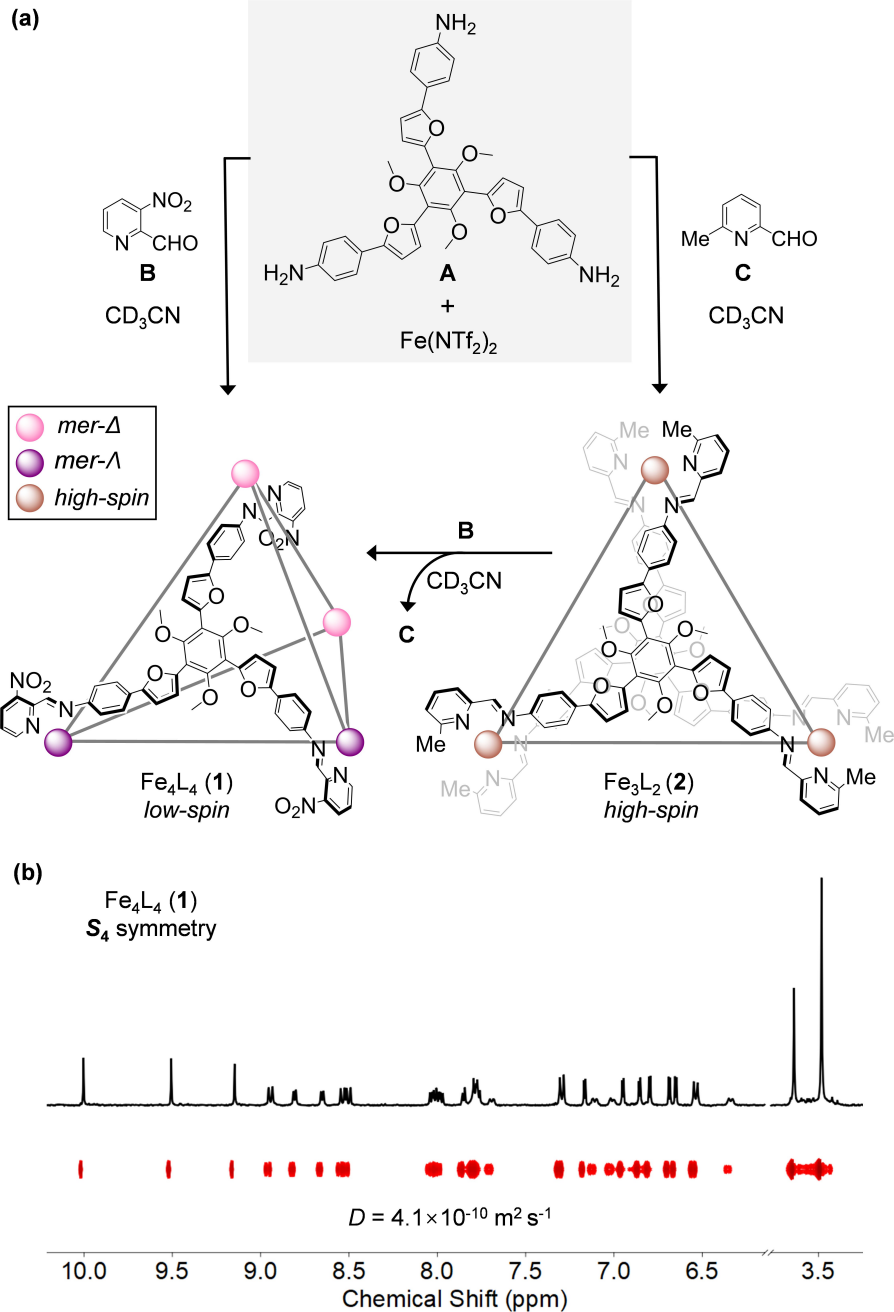
a) Subcomponent self‐assembly of Fe^II^_4_L_4_ capsule **1** and Fe^II^_3_L_2_ sandwich **2** from **A**, Fe^II^(NTf_2_)_2_ and 2‐formylpyridines **B** and **C**, respectively. All reactions were performed at 343 K in CD_3_CN. b) Partial ^1^H NMR and DOSY spectra of *S*_4_‐symmetric **1** (400 MHz, CD_3_CN, 298 K), showing three sets of magnetically‐distinct peaks.

Tritopic aniline subcomponent **A**, with a 1,3,5‐tris(2‐furyl)‐2,4,6‐trimethoxybenzene central core, was synthesized from commercially available 1,3,5‐trimethoxybenzene over four steps (Figure S1). Steric clash between the furan rings and methoxy groups engenders a non‐planar conformation of the central core. The three‐dimensionality and structural flexibility of the core of **A** were elegantly exploited by Fujita, Takezawa et al. in a knotted cage prepared from a related ligand.^[20]^

The reaction of trianiline **A** (4 equiv) and 3‐nitro‐2‐formylpyridine **B** (12 equiv) with iron(II) bis(trifluoromethanesulfonyl)imide (Fe^II^(NTf_2_)_2_, 4 equiv) in CD_3_CN at 343 K for 2 h produced cage **1** (Figure 1a), with its Fe^II^_4_L_4_ composition confirmed by electrospray ionization mass spectrometry (ESI‐MS, Figure S11). The ^1^H NMR spectrum of **1** exhibited three sets of magnetically‐distinct proton signals, with all signals displaying the same diffusion coefficient in the ^1^H diffusion‐ordered spectroscopy (DOSY) spectrum (Figures 1b and S6). ^1^H‐^1^H nuclear Overhauser effect (NOE) correlations between protons of pyridyl and phenylene rings were consistent with low‐symmetry *meridional* (*mer*) configurations at the iron(II) centers (Figure S8).^[21]^ We therefore inferred Fe^II^_4_L_4_ cage **1** to possess *S*_4_ symmetry,^[22]^ in which all iron(II) centers adopt a *mer* stereochemical configuration, with two exhibiting *Δ* handedness, and the other two *Λ*.

In place of subcomponent **B**, other 2‐formylpyridine derivatives were also attempted. Changing the substituent at the 3‐position of the 2‐formylpyridine did not affect the stereochemical outcome during self‐assembly, with the formation of a series of Fe^II^_4_L_4_ cages (**3**–**5**) possessing *S*_4_ symmetry (Figures S14–S23).

Intriguingly, high‐spin complex **2** was formed by the reaction of 6‐methyl‐2‐formylpyridine **C** with **A** and Fe^II^(NTf_2_)_2_ (Figure 1a), as confirmed by the wide‐sweep ^1^H NMR spectrum, with signals in the range −42 to 208 ppm (Figure S12). The ESI‐MS spectrum confirmed the Fe^II^_3_L_2_ composition of **2** (Figure S13). In accordance with the 18‐electron rule,^[23]^ we inferred that each iron(II) vertex of **2** might be surrounded by two bidentate chelating units and two extra solvent molecules, with the high‐spin character of **2** being a consequence of steric clash between methyl groups and the adjacent pyridyl rings around the iron(II) centers, as observed previously.^[24]^ By elongating the Fe^II^−N bonds, such steric repulsion also results in the destabilization of Fe^II^_3_L_2_ **2** relative to Fe^II^_4_L_4_ **1** species. The formation of shorter, stronger Fe^II^−N bonds in **1** was thus inferred to drive subcomponent exchange during the conversion of **2** to **1**, following the addition of 2‐formylpyridine **B** (Figures 1a and S24).

Although many attempts to grow single crystals of **1** were unsuccessful, X‐ray quality crystals of Fe^II^_4_L_4_ analog **3**, constructed from 3‐bromo‐2‐formylpyridine, were obtained by slow vapor diffusion of diethyl ether into an acetonitrile solution of **3** (Figure 2a). The solid‐state structure of **3** adopts idealized *S*_4_ symmetry, consistent with the solution‐state NMR data (Figures 2b and S15).

**Figure 2:**
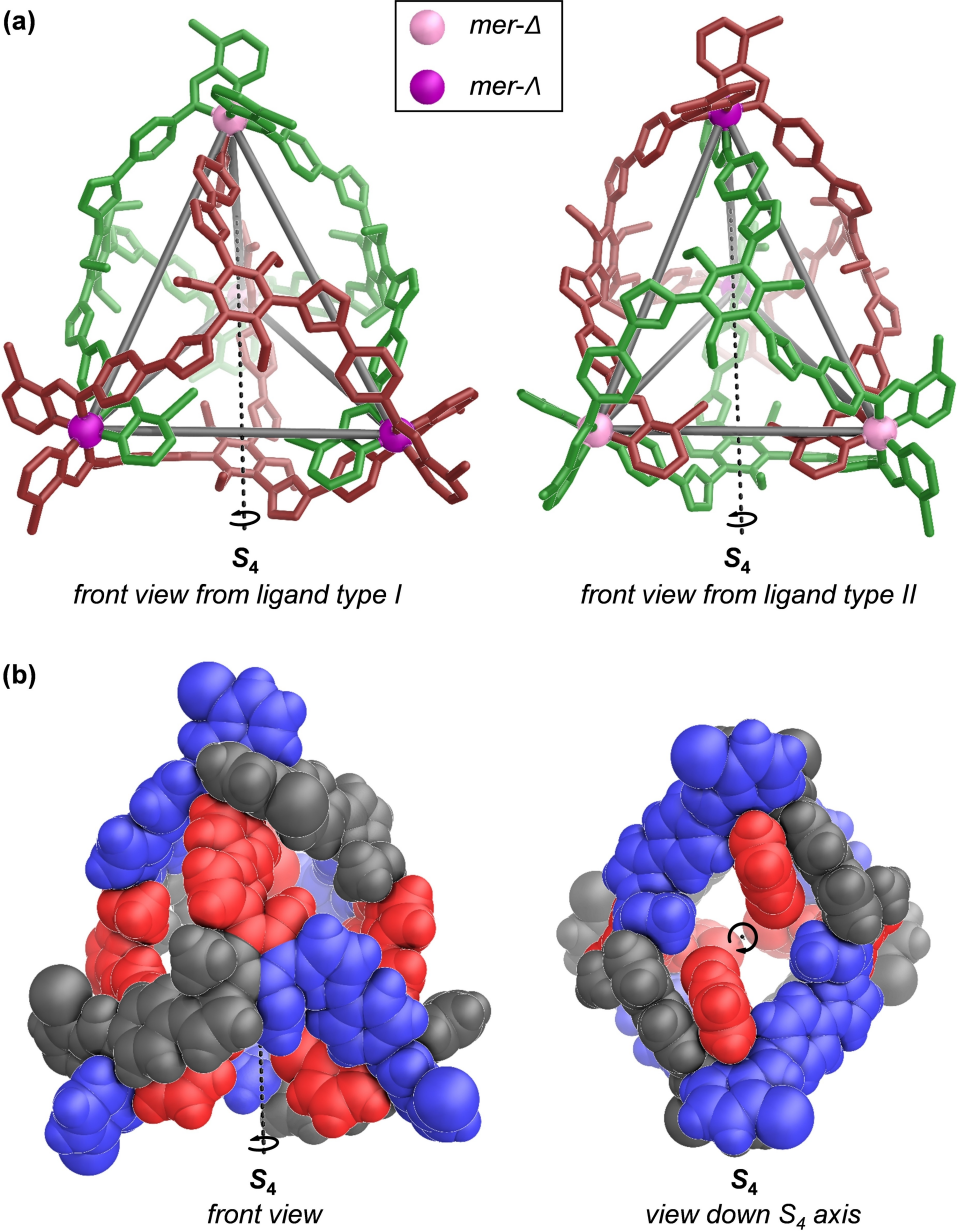
Different views of the crystal structure of Fe^II^_4_L_4_ cage **3**, assembled from 3‐bromo‐2‐formylpyridine, **A** and Fe^II^(NTf_2_)_2_. a) Front views from ligand walls, with clockwise (type I) and anticlockwise (type II) oriented ligands colored brown and green, respectively. b) Front view and view down the *pseudo‐S*_4_ axis, with the ligands shown in space‐filling mode and the three magnetically‐distinct environments shown in blue, red, and black. Disorder, anions, solvents and hydrogen atoms are omitted for clarity.

The structure of **3** consists of four face‐capping ligands, with two ligands adopting a clockwise orientation and the other two anticlockwise. These ligands bridge four iron(II) vertices, two *mer*‐*Δ* and two *mer*‐*Ʌ*. The mean Fe^II^⋅⋅⋅Fe^II^ distance along the four edges between *Δ* and *Ʌ* metal centers is 18.1±0.3 Å; the Fe^II^⋅⋅⋅Fe^II^ distance between the two *mer*‐*Λ* metal centers (15.4 Å) is slightly longer than between the two *mer*‐*Δ* metal centers (14.4 Å). A cavity volume of 986 Å^3^ for **3** was calculated using the MoloVol program.^[25]^

In the structure of **3**, the three dihedral angles between the central phenyl ring and the furan rings within the same clockwise face‐capping ligand were observed to differ (40.1°, 42.8°, 44.5°), as were the three torsion angles (52.2°, 58.2°, 62.2°) between the phenylene rings and the N−Fe^II^−N chelate planes around each *mer*‐*Ʌ* metal vertex. These differing angles thus reflect the structural flexibility and plasticity of the Fe^II^_4_L_4_ framework to adopt different ligand configurations as required to minimize the energy of the system.

Encouraged by the flexible nature of its *S*_4_‐symmetric Fe^II^_4_L_4_ framework and enclosed cavity, we next investigated the structural and stereochemical adaptability of **1** upon guest binding. Cage **1** was observed to bind both neutral and anionic guests (Table 1), with binding affinities quantified by ^1^H NMR titrations, and binding stoichiometries gauged by NMR or ESI‐MS, as detailed in Supporting Information Section 5.

**Table 1:** Host‐Guest Properties of **1**.

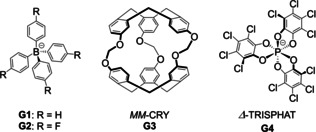		
Guest^[a]^	*K*_a_ [M^−1^]^[b]^	Host Symmetry^[c]^
**G1**	(1.82±0.05)×10^2^	*S*_4_
**G2**	(1.21±0.05)×10^2^	*S*_4_
**G3** ^[d]^	(2.33±0.10)×10^3^	*T*
**G4**	(1.17±0.15)×10^5^	*C*_3_

[a] ^1^H NMR titrations were performed by the addition of aliquots of a CD_3_CN solution of **G1** or **G2** (50 mM) into a stock solution of **1** in CD_3_CN (0.5 mM); ^1^H NMR titrations were performed by portionwise addition of **G3** or **G4** as a solid into a stock solution of **1** in CD_3_CN (0.75 mM) using *tert*‐butyl acetate as an internal standard. [b] Binding constant determined by ^1^H NMR titration. [c] Host point symmetry inferred from NMR spectroscopy. [d] The binding constant for *PP*‐CRY (**G3′**) of *K*_a_=(2.40±0.10)×10^3^ M^−1^ was identical within error.

Upon addition of tetraphenylborate **G1** or **G2**, the proton signals of **1** were observed to shift, in line with fast guest exchange on the NMR time scale (Figures S25 and S29). The signals of the cage furan and phenylene rings as well as the OMe groups at the apertures of **1** shifted most. In addition, when the central cavity of **1** (ca. 986 Å^3^) was occupied by **G3** (596 Å^3^) or **G4** (471 Å^3^), **1** was observed to bind **G1** (321 Å^3^) simultaneously, as shown in Figure 4. These observations led us to infer that **G1** and **G2** are bound peripherally at the edges of **1**.^[17c]^ The exact binding stoichiometries could not be gauged using ^1^H NMR titrations (Figures S27 and S31), which fitted slightly, but not overwhelmingly better to a 1 : 2 model using BindFit.^[26]^ Chemical shift changes for **1** were plotted and fitted to a Hill function (Figures S28 and S32),^[27]^ with apparent association constants determined to be (1.82±0.05)×10^2^ M^−1^ and (1.21±0.05)×10^2^ M^−1^ for **G1** and **G2**, respectively. In both cases, the Hill coefficients were approximately 1, indicating non‐cooperative binding of tetraphenylborates to **1**. Putative peripheral binding of tetraphenylborates during the titration process did not alter the *S*_4_ symmetry of the framework of **1** (Figure 3b).

**Figure 3:**
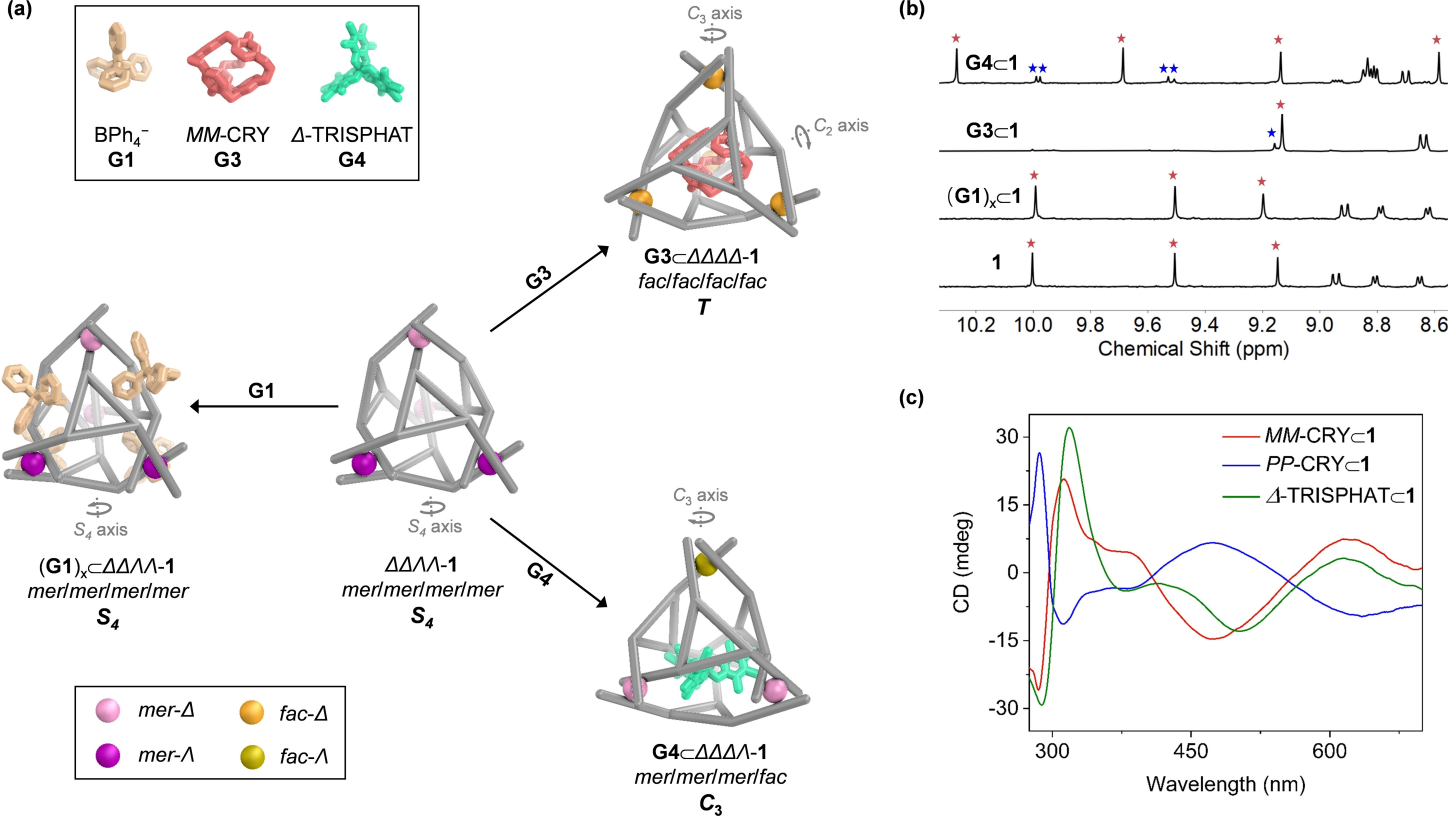
a) Schematic diagram showing the symmetry and stereochemical adaptation of **1** driven by guest binding. b) Partial ^1^H NMR spectra of the imine region of **1** and its host–guest complexes (400 MHz, CD_3_CN, 298 K), with imine peaks marked by stars. For **G3**⊂**1** and **G4**⊂**1**, red stars correspond to the major host–guest complex diastereomer (**G3**⊂*ΔΔΔΔ*‐**1** or **G4**⊂*ΔΔΔΛ*‐**1**), whereas blue stars correspond to the minor diastereomer with an enantiomorphous host configuration. c) CD spectra of host–guest complexes in acetonitrile at equal concentrations.

By contrast, enantiopure guest **G3**, *MM*‐cryptophane (CRY),[^25^, ^28^] with a calculated volume of 596 Å^3^ was observed to bind centrally within the cavity of **1** in slow exchange on the NMR time scale, with a binding constant of (2.33±0.10)×10^3^ M^−1^ (Figures 3a and S33). During titration experiments, proton signals corresponding to **G3**⊂**1** emerged as free **1** disappeared from the ^1^H NMR spectrum. NMR integration revealed the formation of a 1 : 1 host–guest complex. All proton signals from the bound guest shifted significantly upfield as a result of shielding effects, consistent with central binding of **G3** (Figure S35). Interestingly, cage **1** was not observed to encapsulate C_60_ (549 Å^3^), which we attribute to a poor fit between the bumpy inner surface of the host and the smooth curvature of the guest, precluding effective stacking interactions between them.

The ^1^H NMR spectrum of **G3**⊂**1** was consistent with the formation of a *T*‐symmetric species (Figures 3b and S35), with ligands in a threefold‐symmetric environment and all metal centers adopting *facial* (*fac*) stereochemical configurations with the same handedness, as observed in high‐symmetry face‐capped tetrahedra.^[29]^ The presence of *fac* stereochemistry was also confirmed by the absence of characteristic NOE correlations between protons on the pyridyl and phenylene rings (Figure S38).

Two groups of proton peaks in a ratio of 4.8 : 1 were observed in the ^1^H NMR spectrum, indicating that **G3**⊂**1** existed as a pair of diastereomers, *MM–*CRY⊂*ΔΔΔΔ*‐**1** and *MM–*CRY⊂*ΛΛΛΛ*‐**1**. The circular dichroism (CD) spectrum of **G3**⊂**1** displayed strong Cotton effects in the ranges 280–400 nm and 410–680 nm, assigned to π–π* and metal‐to‐ligand charge transfer (MLCT) transitions, respectively (Figure 3c). The negative sign of the MLCT bands correlates with *Δ* handedness,^[30]^ suggesting that the major diastereomer of *MM–*CRY⊂**1** has four *fac*‐*Δ* metal vertices. Cage **1** also bound the enantiomer *PP*‐CRY to yield *PP*‐CRY⊂**1** possessing four *fac*‐*Ʌ* metal centers as the major diastereomer, with an identical‐within‐error binding affinity of *K*_a_=(2.40±0.10)×10^3^ M^−1^ (Figures S34 and S40).

Host‐guest investigations also revealed that **1** was able to accommodate an equimolar amount of *Δ*‐tris(tetrachloro‐1,2‐benzenediolato)phosphate (*Δ*‐TRISPHAT, **G4**)^[31]^ with a binding affinity of *K*_a_=(1.17±0.15)×10^5^ M^−1^. When more than one equivalent of *Δ*‐TRISPHAT was added during NMR titrations, no further changes were observed in ^1^H and ^31^P NMR spectra (Figures S44 and S45), consistent with the formation of a 1 : 1 host–guest complex **G4**⊂**1**, as was also observed by ESI‐MS (Figure S51). The ^1^H NMR spectrum had two groups of signals, with each group containing four sets of signals (Figures 3b and S46), consistent with the presence of two *C*_3_‐symmetric diastereomers of **G4**⊂**1** in a 4.1 : 1 ratio. NOE correlations provided evidence for a 1 : 3 *fac*:*mer* configuration of metal centers for the major diastereomer (Figure S49), while the MLCT bands in the CD spectrum indicated an excess of *Δ* stereochemistry within **G4**⊂**1** (Figure 3c). We thus inferred that the major diastereomer of **G4**⊂**1** with *C*_3_ symmetry contains one *fac*‐*Ʌ* and three *mer*‐*Δ* metal centers, as in the elegant “sorting hat” structure reported by Hooley and co‐workers.^[32]^

The conversion of *S*_4_‐symmetric **1** into *T*‐ and *C*_3_‐symmetric host–guest complexes did not occur at low temperatures, requiring to be heated at 343 K for 2 h to complete these transformations. The high temperatures required to overcome the energy barriers for this symmetry breaking and rearranging suggested a mechanism involving extensive disassembly and reassembly.^[33]^

Having identified that **1** has two distinct binding sites that can bind different guests, we then explored allosteric effects by simultaneously treating **1** with two different guests (Figure 4). To consider cooperative effects, we determined the factor *α*=*K*′_a_/*K*_a_ to quantify allosteric regulation, where *K*′_a_ and *K*_a_ are the binding constants of a guest bound by the host–guest complex and the empty host, respectively. A value of *α*>1 indicates positive cooperativity, whereas *α*<1 suggests negative cooperativity.[^2^, ^26a^]

**Figure 4:**
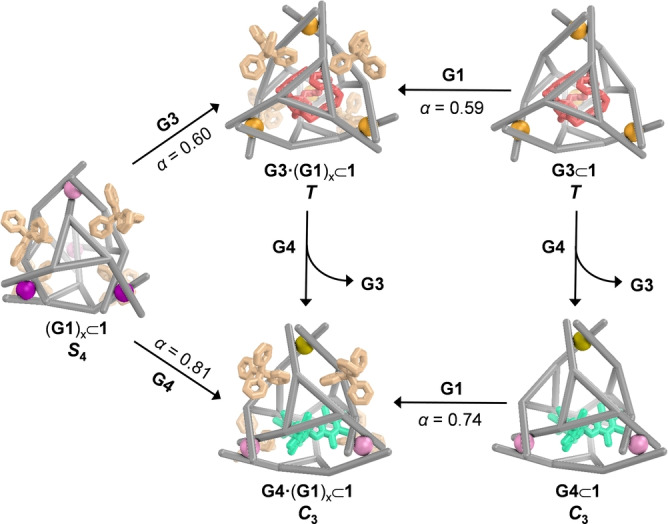
Schematic showing the allosteric effects and cooperative binding.

Both *T*‐symmetric **G3**⊂**1** and *C*_3_‐symmetric **G4**⊂**1** maintained their symmetries upon binding tetraphenylborate in fast exchange on the NMR time scale (Figure 4). Negative cooperativity (*α*=0.59 and 0.74) was observed in both cases (Figures S52–S57). When **1** bound BPh_4_^−^ peripherally, the central binding of both **G3** and **G4** was likewise inhibited. Negative cooperative effects (*α*=0.60 and 0.81) were observed on the binding of these two guests, which gave rise to *T*‐symmetric **G3** ⋅ **(G1)_x_** ⊂**1** and *C*_3_‐symmetric **G4** ⋅ **(G1)_x_** ⊂**1**, respectively (Figures S58 and S59). This negative cooperativity was inferred to be a consequence of changes in aperture size and cavity volumes that took place in order to optimize binding, but which do not favor the binding of two guests at once. The binding of **G3** or **G4** within the cavity of **1** may also physically block **G1** from reaching inside the windows to bind. The presence of an electron‐rich anionic or neutral guests within electron‐deficient **1** may also weaken the binding of further electron‐rich guests by electrostatic repulsion.

The higher binding affinity of **G4** over **G3** allowed for guest displacement, thus enabling the conversion of the *T*‐symmetric cage framework into the *C*_3_‐symmetric one (Figures S60 and S61). The most strongly‐binding guest in the system thus dictated the framework symmetry.

The ability of **1** to adopt three distinct diastereomeric conformations in order to optimize guest binding, including the singular *S*_4_ framework, with two *mer*‐*Δ* and two *mer*‐*Ʌ* metal vertices, thus complements and builds usefully upon previous studies of cage stereochemistry. Its two allosterically active sites enabled the simultaneous binding of two distinct guests, peripherally and centrally. Future work may enable such allosteric effects to control reactions that are catalyzed within the cage cavity or at a peripheral site, potentially allowing up‐ or down‐regulation of catalysis in biomimetic fashion.

## Conflict of interest

The authors declare no conflict of interest.

## Supporting information

As a service to our authors and readers, this journal provides supporting information supplied by the authors. Such materials are peer reviewed and may be re‐organized for online delivery, but are not copy‐edited or typeset. Technical support issues arising from supporting information (other than missing files) should be addressed to the authors.

Supporting Information

Supporting Information

Supporting Information

## Data Availability

The data that support the findings of this study are available in the Supporting Information of this article.

## References

[1] D. E. Koshland, Jr. , Angew. Chem. Int. Ed. 1994, 33, 2375–2378;

[2a] C. A. Hunter , H. L. Anderson , Angew. Chem. Int. Ed. 2009, 48, 7488–7499; 10.1002/anie.200902490 19746372

[2b] A. S. Mahadevi , G. N. Sastry , Chem. Rev. 2016, 116, 2775–2825. 26840650 10.1021/cr500344e

[3a] C. Colomban , G. Szalóki , M. Allain , L. Gómez , S. Goeb , M. Sallé , M. Costas , X. Ribas , Chem. Eur. J. 2017, 23, 3016–3022; 28112436 10.1002/chem.201700273

[3b] R. Djemili , L. Kocher , S. Durot , A. Peuronen , K. Rissanen , V. Heitz , Chem. Eur. J. 2019, 25, 1481–1487; 30536482 10.1002/chem.201805498

[3c] V. Martí-Centelles , R. L. Spicer , P. J. Lusby , Chem. Sci. 2020, 11, 3236–3240. 34122830 10.1039/d0sc00341g PMC8157338

[4a] A. M. Lifschitz , M. S. Rosen , C. M. McGuirk , C. A. Mirkin , J. Am. Chem. Soc. 2015, 137, 7252–7261; 26035450 10.1021/jacs.5b01054

[4b] P. W. J. M. Frederix , I. Patmanidis , S. J. Marrink , Chem. Soc. Rev. 2018, 47, 3470–3489; 29688238 10.1039/c8cs00040a PMC5961611

[4c] F. J. Rizzuto , L. K. S. von Krbek , J. R. Nitschke , Nat. Chem. Rev. 2019, 3, 204–222;

[4d] A. E. Martín Díaz , J. E. Lewis , Front. Chem. 2021, 9, 706462. 34336791 10.3389/fchem.2021.706462 PMC8317845

[5] For selected examples on molecular sensing and recognition, see:

[5a] W. Xuan , M. Zhang , Y. Liu , Z. Chen , Y. Cui , J. Am. Chem. Soc. 2012, 134, 6904–6907; 22494630 10.1021/ja212132r

[5b] J. Dong , Y. Zhou , F. Zhang , Y. Cui , Chem. Eur. J. 2014, 20, 6455–6461; 24710843 10.1002/chem.201304606

[5c] A. J. Plajer , E. G. Percástegui , M. Santella , F. J. Rizzuto , Q. Gan , B. W. Laursen , J. R. Nitschke , Angew. Chem. Int. Ed. 2019, 58, 4200–4204; 10.1002/anie.201814149 30666756

[5d] T. R. Schulte , J. J. Holstein , G. H. Clever , Angew. Chem. Int. Ed. 2019, 58, 5562–5566; 10.1002/anie.201812926 PMC6563462 30761694

[5e] P. Howlader , E. Zangrando , P. S. Mukherjee , J. Am. Chem. Soc. 2020, 142, 9070–9078. 32315163 10.1021/jacs.0c03551

[6] For selected examples and reviews on chemical separation, see:

[6a] K. Wu , K. Li , Y.-J. Hou , M. Pan , L.-Y. Zhang , L. Chen , C.-Y. Su , Nat. Commun. 2016, 7, 10487; 26839048 10.1038/ncomms10487 PMC4742817

[6b] C. Fuertes-Espinosa , A. Gomez-Torres , R. Morales-Martinez , A. Rodriguez-Fortea , C. Garcia-Simon , F. Gandara , I. Imaz , J. Juanhuix , D. Maspoch , J. M. Poblet , L. Echegoyen , X. Ribas , Angew. Chem. Int. Ed. 2018, 57, 11294–11299; 10.1002/anie.201806140 29917307

[6c] C. Fuertes-Espinosa , M. Pujals , X. Ribas , Chem 2020, 6, 3219–3262;

[6d] D. Zhang , T. K. Ronson , Y.-Q. Zou , J. R. Nitschke , Nat. Chem. Rev. 2021, 5, 168–182; 10.1038/s41570-020-00246-1 37117530

[6e] A. B. Sainaba , M. Venkateswarulu , P. Bhandari , K. S. A. Arachchige , J. K. Clegg , P. S. Mukherjee , J. Am. Chem. Soc. 2022, 144, 7504–7513. 35436087 10.1021/jacs.2c02540

[7] For selected examples and reviews on stabilizing reactive species, see:

[7a] P. Mal , B. Breiner , K. Rissanen , J. R. Nitschke , Science 2009, 324, 1697–1699; 19556504 10.1126/science.1175313

[7b] M. Yamashina , Y. Sei , M. Akita , M. Yoshizawa , Nat. Commun. 2014, 5, 4662; 25130933 10.1038/ncomms5662

[7c] A. Galan , P. Ballester , Chem. Soc. Rev. 2016, 45, 1720–1737; 26797259 10.1039/c5cs00861a

[7d] S. Hasegawa , S. L. Meichsner , J. J. Holstein , A. Baksi , M. Kasanmascheff , G. H. Clever , J. Am. Chem. Soc. 2021, 143, 9718–9723. 34156243 10.1021/jacs.1c02860

[8] For selected examples and reviews on cage catalysis, see:

[8a] M. Yoshizawa , M. Tamura , M. Fujita , Science 2006, 312, 251–254; 16614218 10.1126/science.1124985

[8b] C. J. Brown , R. G. Bergman , K. N. Raymond , J. Am. Chem. Soc. 2009, 131, 17530–17531; 19950985 10.1021/ja906386w

[8c] C. García-Simón , R. Gramage-Doria , S. Raoufmoghaddam , T. Parella , M. Costas , X. Ribas , J. N. H. Reek , J. Am. Chem. Soc. 2015, 137, 2680–2687; 25632976 10.1021/ja512637k

[8d] D. M. Kaphan , M. D. Levin , R. G. Bergman , K. N. Raymond , F. D. Toste , Science 2015, 350, 1235–1238; 26785485 10.1126/science.aad3087

[8e] W. Cullen , M. C. Misuraca , C. A. Hunter , N. H. Williams , M. D. Ward , Nat. Chem. 2016, 8, 231–236; 26892554 10.1038/nchem.2452

[8f] W. Cullen , A. J. Metherell , A. B. Wragg , C. G. P. Taylor , N. H. Williams , M. D. Ward , J. Am. Chem. Soc. 2018, 140, 2821–2828; 29412665 10.1021/jacs.7b11334

[8g] C. Tan , D. Chu , X. Tang , Y. Liu , W. Xuan , Y. Cui , Chem. Eur. J. 2019, 25, 662–672; 30076749 10.1002/chem.201802817

[8h] J. Guo , Y.-Z. Fan , Y.-L. Lu , S.-P. Zheng , C.-Y. Su , Angew. Chem. Int. Ed. 2020, 59, 8661–8669; 10.1002/anie.201916722 32011801

[8i] M. Morimoto , S. M. Bierschenk , K. T. Xia , R. G. Bergman , K. N. Raymond , F. D. Toste , Nat. Catal. 2020, 3, 969–984;

[8j] G. Olivo , G. Capocasa , D. Del Giudice , O. Lanzalunga , S. Di Stefano , Chem. Soc. Rev. 2021, 50, 7681–7724; 34008654 10.1039/d1cs00175b

[8k] C. Ngai , H.-T. Wu , B. da Camara , C. G. Williams , L. J. Mueller , R. R. Julian , R. J. Hooley , Angew. Chem. Int. Ed. 2022, 61, e202117011; 10.1002/anie.202117011 PMC8885886 35030288

[9a] W. Wang , Y.-X. Wang , H.-B. Yang , Chem. Soc. Rev. 2016, 45, 2656–2693; 27009833 10.1039/c5cs00301f

[9b] J. D. Crowley , L. S. Lisboa , Q. V. C. van Hilst in Comprehensive Coordination Chemistry III (Eds.: E. C. Constable , G. Parkin , L. Que, Jr. ), Elsevier, Oxford, 2021, pp. 174–205.

[10] D. Zhang , T. K. Ronson , S. Güryel , J. D. Thoburn , D. J. Wales , J. R. Nitschke , J. Am. Chem. Soc. 2019, 141, 14534–14538. 31478658 10.1021/jacs.9b07307 PMC6753657

[11] H. Lee , J. Tessarolo , D. Langbehn , A. Baksi , R. Herges , G. H. Clever , J. Am. Chem. Soc. 2022, 144, 3099–3105. 35081312 10.1021/jacs.1c12011 PMC8874908

[12] V. Croué , S. Goeb , G. Szalóki , M. Allain , M. Sallé , Angew. Chem. Int. Ed. 2016, 55, 1746–1750; 10.1002/anie.201509265 26693832

[13] L. S. Lisboa , J. A. Findlay , L. J. Wright , C. G. Hartinger , J. D. Crowley , Angew. Chem. Int. Ed. 2020, 59, 11101–11107; 10.1002/anie.202003220 32220036

[14a] R. W. Saalfrank , A. Stark , K. Peters , H. G. von Schnering , Angew. Chem. Int. Ed. Engl. 1988, 27, 851–853;

[14b] D. L. Caulder , R. E. Powers , T. N. Parac , K. N. Raymond , Angew. Chem. Int. Ed. 1998, 37, 1840–1843;

[14c] P. Mal , D. Schultz , K. Beyeh , K. Rissanen , J. R. Nitschke , Angew. Chem. Int. Ed. 2008, 47, 8297–8301; 10.1002/anie.200803066 18729112

[14d] Y. Liu , X. Wu , C. He , Y. Jiao , C. Duan , Chem. Commun. 2009, 7554–7556; 10.1039/b915358f 20024277

[14e] L.-L. Yan , C.-H. Tan , G.-L. Zhang , L.-P. Zhou , J.-C. Bünzli , Q.-F. Sun , J. Am. Chem. Soc. 2015, 137, 8550–8555. 26065490 10.1021/jacs.5b03972

[15a] M. Fujita , D. Oguro , M. Miyazawa , H. Oka , K. Yamaguchi , K. Ogura , Nature 1995, 378, 469–471;

[15b] P. J. Stang , B. Olenyuk , D. C. Muddiman , R. D. Smith , Organometallics 1997, 16, 3094–3096;

[15c] O. Chepelin , J. Ujma , X. Wu , A. M. Z. Slawin , M. B. Pitak , S. J. Coles , J. Michel , A. C. Jones , P. E. Barran , P. J. Lusby , J. Am. Chem. Soc. 2012, 134, 19334–19337. 23137068 10.1021/ja309031h

[16a] I. S. Tidmarsh , T. B. Faust , H. Adams , L. P. Harding , L. Russo , W. Clegg , M. D. Ward , J. Am. Chem. Soc. 2008, 130, 15167–15175; 18855358 10.1021/ja805605y

[16b] W. Meng , B. Breiner , K. Rissanen , J. D. Thoburn , J. K. Clegg , J. R. Nitschke , Angew. Chem. Int. Ed. 2011, 50, 3479–3483; 10.1002/anie.201100193 21394866

[16c] X.-P. Zhou , J. Liu , S.-Z. Zhan , J.-R. Yang , D. Li , K.-M. Ng , R. W.-Y. Sun , C.-M. Che , J. Am. Chem. Soc. 2012, 134, 8042–8045; 22545574 10.1021/ja302142c

[16d] Y. Yang , J.-H. Jia , X.-L. Pei , H. Zheng , Z.-A. Nan , Q.-M. Wang , Chem. Commun. 2015, 51, 3804–3807. 10.1039/c5cc00087d 25649958

[17a] B. Olenyuk , J. A. Whiteford , A. Fechtenkötter , P. J. Stang , Nature 1999, 398, 796–799; 10235260 10.1038/19740

[17b] K. Ghosh , J. Hu , H. S. White , P. J. Stang , J. Am. Chem. Soc. 2009, 131, 6695–6697; 19397325 10.1021/ja902045q PMC2775065

[17c] F. J. Rizzuto , J. R. Nitschke , Nat. Chem. 2017, 9, 903–908. 28837174 10.1038/nchem.2758

[18a] M. Wang , C. Wang , X.-Q. Hao , X. Li , T. J. Vaughn , Y.-Y. Zhang , Y. Yu , Z.-Y. Li , M.-P. Song , H.-B. Yang , X. Li , J. Am. Chem. Soc. 2014, 136, 10499–10507; 24978202 10.1021/ja505414x

[18b] M. Han , Y. Luo , B. Damaschke , L. Gómez , X. Ribas , A. Jose , P. Peretzki , M. Seibt , G. H. Clever , Angew. Chem. Int. Ed. 2016, 55, 445–449; 10.1002/anie.201508307 26609916

[18c] D. Fujita , Y. Ueda , S. Sato , H. Yokoyama , N. Mizuno , T. Kumasaka , M. Fujita , Chem 2016, 1, 91–101;

[18d] D. Luo , X.-Z. Wang , C. Yang , X.-P. Zhou , D. Li , J. Am. Chem. Soc. 2018, 140, 118–121; 29235858 10.1021/jacs.7b11285

[18e] H. Wang , K. Wang , Y. Xu , W. Wang , S. Chen , M. Hart , L. Wojtas , L.-P. Zhou , L. Gan , X. Yan , Y. Li , J. Lee , X.-S. Ke , X.-Q. Wang , C.-W. Zhang , S. Zhou , T. Zhai , H.-B. Yang , M. Wang , J. He , Q.-F. Sun , B. Xu , Y. Jiao , P. J. Stang , J. L. Sessler , X. Li , J. Am. Chem. Soc. 2021, 143, 5826–5835. 33848163 10.1021/jacs.1c00625

[19a] J. E. M. Lewis , J. D. Crowley , ChemPlusChem 2020, 85, 815–827; 32364332 10.1002/cplu.202000153

[19b] C. T. McTernan , J. A. Davies , J. R. Nitschke , Chem. Rev. 2022, 122, 10393–10437; 35436092 10.1021/acs.chemrev.1c00763 PMC9185692

[19c] J. E. M. Lewis , Chem. Commun. 2022, 58, 13873–13886. 10.1039/d2cc05560k 36448362

[20] Y. Tamura , H. Takezawa , M. Fujita , J. Am. Chem. Soc. 2020, 142, 5504–5508. 32149516 10.1021/jacs.0c00459

[21] F. J. Rizzuto , P. Pröhm , A. J. Plajer , J. L. Greenfield , J. R. Nitschke , J. Am. Chem. Soc. 2019, 141, 1707–1715. 30612431 10.1021/jacs.8b12323

[22a] M. Hardy , M. Engeser , A. Lützen , Beilstein J. Org. Chem. 2020, 16, 2701–2708; 33214795 10.3762/bjoc.16.220 PMC7653331

[22b] Q. Shi , X. Zhou , W. Yuan , X. Su , A. Neniškis , X. Wei , L. Taujenis , G. Snarskis , J. S. Ward , K. Rissanen , J. de Mendoza , E. Orentas , J. Am. Chem. Soc. 2020, 142, 3658–3670. 31983204 10.1021/jacs.0c00722

[23] C. A. Tolman , Chem. Soc. Rev. 1972, 1, 337–353.

[24] A. J. McConnell , C. M. Aitchison , A. B. Grommet , J. R. Nitschke , J. Am. Chem. Soc. 2017, 139, 6294–6297. 28426930 10.1021/jacs.7b01478 PMC5537689

[25] J. B. Maglic , R. Lavendomme , J. Appl. Crystallogr. 2022, 55, 1033–1044. 35974729 10.1107/S1600576722004988 PMC9348874

[26a] P. Thordarson , Chem. Soc. Rev. 2011, 40, 1305–1323; 21125111 10.1039/c0cs00062k

[26b] H. Takezawa , T. Murase , G. Resnati , P. Metrangolo , M. Fujita , J. Am. Chem. Soc. 2014, 136, 1786–1788; 24422785 10.1021/ja412893c

[26c] D. Brynn Hibbert , P. Thordarson , Chem. Commun. 2016, 52, 12792–12805. 10.1039/c6cc03888c 27779264

[27] G. Ercolani , J. Am. Chem. Soc. 2003, 125, 16097–16103. 14678002 10.1021/ja038396c

[28a] Van der Waals Volume of *MM*-cryptophane was calculated by MoloVol program, using reported crystal structures, see: T. Buffeteau , D. Pitrat , N. Daugey , N. Calin , M. Jean , N. Vanthuyne , L. Ducasse , F. Wien , T. Brotin , Phys. Chem. Chem. Phys. 2017, 19, 18303–18310; 28676874 10.1039/c7cp02045g

[28b] D. Zhang , A. Martinez , J.-P. Dutasta , Chem. Rev. 2017, 117, 4900–4942. 28277650 10.1021/acs.chemrev.6b00847

[29a] C. M. Hong , D. M. Kaphan , R. G. Bergman , K. N. Raymond , F. D. Toste , J. Am. Chem. Soc. 2017, 139, 8013–8021; 28581740 10.1021/jacs.7b03812

[29b] D. Zhang , T. K. Ronson , J. L. Greenfield , T. Brotin , P. Berthault , E. Léonce , J.-L. Zhu , L. Xu , J. R. Nitschke , J. Am. Chem. Soc. 2019, 141, 8339–8345; 31034215 10.1021/jacs.9b02866

[29c] S.-J. Hu , X.-Q. Guo , L.-P. Zhou , D.-N. Yan , P.-M. Cheng , L.-X. Cai , X.-Z. Li , Q.-F. Sun , J. Am. Chem. Soc. 2022, 144, 4244–4253. 35195993 10.1021/jacs.2c00760

[30a] S. E. Howson , L. E. N. Allan , N. P. Chmel , G. J. Clarkson , R. J. Deeth , A. D. Faulkner , D. H. Simpson , P. Scott , Dalton Trans. 2011, 40, 10416–10433; 21743936 10.1039/c1dt10588d

[30b] J. M. Dragna , G. Pescitelli , L. Tran , V. M. Lynch , E. V. Anslyn , L. Di Bari , J. Am. Chem. Soc. 2012, 134, 4398–4407. 22272943 10.1021/ja211768v PMC3329375

[31] J. Lacour , C. Ginglinger , C. Grivet , G. Bernardinelli , Angew. Chem. Int. Ed. 1997, 36, 608–610;

[32] M. C. Young , L. R. Holloway , A. M. Johnson , R. J. Hooley , Angew. Chem. Int. Ed. 2014, 53, 9832–9836; 10.1002/anie.201405242 25044629

[33] W. Meng , J. K. Clegg , J. D. Thoburn , J. R. Nitschke , J. Am. Chem. Soc. 2011, 133, 13652–13660. 21790184 10.1021/ja205254s

[34] Deposition Numbers 2235215 (for cage **3**) contains the supplementary crystallographic data for this paper. These data are provided free of charge by the joint Cambridge Crystallographic Data Centre and Fachinformationszentrum Karlsruhe Access Structures service.

