# Supplementary material for: Allosterically Regulated Guest Binding Determines Framework Symmetry for an FeII 4L4 Cage
Source: Angew Chem Int Ed Engl. 2023 Mar 28;62(18):e202301319. doi: 10.1002/anie.202301319 (PMC10947561; doi:10.1002/anie.202301319)

checkCIF/PLATON report

You have not supplied any structure factors. As a result the full set of tests cannot be run.

THIS REPORT IS FOR GUIDANCE ONLY. IF USED AS PART OF A REVIEW PROCEDURE FOR PUBLICATION, IT SHOULD NOT REPLACE THE EXPERTISE OF AN EXPERIENCED CRYSTALLOGRAPHIC REFEREE.

No syntax errors found. CIF dictionary Interpreting this report

Datablock: xwc399_sq

Bond precision:	C-C = 0.0199 A		Wavelength=0.68890
Cell:	a=38.8028(3)	b=24.9335(2)	c=35.8662(3)
	alpha=90	beta=95.670(1)	gamma=90
Temperature:	100 K		

	Calculated	Reported
Volume	34530.4(5)	34530.4(5)
Space group	P 2/c	P 2/c
Hall group	-P 2yc	-P 2yc
Moiety formula	2(C228 H156 Br12 Fe4 N24 O24), 16(C2 F6 N O4 S2), 7(C4 H10 O),	?
Sum formula	C532 H406 Br24 F96 Fe8 N72 O119 S32 [+ solvent]	C532 H406 Br24 F96 Fe8 N72 O119 S32
Mr	14925.63	14925.82
Dx,g cm-3	1.436	1.436
Z	2	2
Mu (mm-1)	1.609	1.747
F000	14956.0	14956.0
F000’	14967.78	
h,k,lmax	35,22,32	35,22,32
Nref	27192	27151
Tmin,Tmax	0.840,0.840	0.893,1.000
Tmin’	0.840	

Correction method= # Reported T Limits: Tmin=0.893 Tmax=1.000
AbsCorr = EMPIRICAL

Data completeness= 0.998 Theta(max)= 18.248

R(reflections)= 0.1247(14325)

wR2(reflections)= 0.3721(27151)

S = 1.295 Npar= 4124

The following ALERTS were generated. Each ALERT has the format
test-name_ALERT_alert-type_alert-level.
Click on the hyperlinks for more details of the test.

Alert level A

THETM01_ALERT_3_A The value of sine(theta_max)/wavelength is less than 0.550
Calculated sin(theta_max)/wavelength = 0.4545

Author Response: Disorder in the solvent region and extremely poor scattering power of the crystal does not allow for higher order data to be collected, even though using a synchrotron source.

Alert level B

PLAT084_ALERT_3_B High wR2 Value (i.e. > 0.25) .... 0.37 Report

Author Response: The high wR2 reflects the flexibility of the structure and weak crystal quality. Nevertheless geometry and ADP restraints ensure a chemically and physically meaningful structural model.

PLAT088_ALERT_3_B Poor Data / Parameter Ratio .... 6.59 Note

Author Response: With this structure we are clearly sailing into the macromolecular domain of structures and have extensively used restraints in a macromolecular fashion to compensate for limited observations.

PLAT241_ALERT_2_B High 'MainMol' Ueq as Compared to Neighbors of N1_8 Check

Author Response: The B level alert results from the high level of thermal motion round the NTf2 anions. The C level alerts of this type result from the generally high level of thermal motion throughout the structure.

PLAT241_ALERT_2_B High 'MainMol' Ueq as Compared to Neighbors of S1_9 Check

Author Response: The B level alert results from the high level of thermal motion round the NTf2 anions. The C level alerts of this type result from the generally high level of thermal motion throughout the structure.

```
PLAT242_ALERT_2_B Low    'MainMol' Ueq as Compared to Neighbors of       S1_8 Check
```

Author Response: The B level alert results from the high level of thermal motion round the NTf2 anions. The C level alerts of this type result from the generally high level of thermal motion throughout the structure.

```
PLAT242_ALERT_2_B Low    'MainMol' Ueq as Compared to Neighbors of       S2_8 Check
```

Author Response: The B level alert results from the high level of thermal motion round the NTf2 anions. The C level alerts of this type result from the generally high level of thermal motion throughout the structure.

```
PLAT242_ALERT_2_B Low    'MainMol' Ueq as Compared to Neighbors of       N1_9 Check
```

Author Response: The B level alert results from the high level of thermal motion round the NTf2 anions. The C level alerts of this type result from the generally high level of thermal motion throughout the structure.

```
PLAT242_ALERT_2_B Low    'MainMol' Ueq as Compared to Neighbors of      S1_12 Check
```

Author Response: The B level alert results from the high level of thermal motion round the NTf2 anions. The C level alerts of this type result from the generally high level of thermal motion throughout the structure.

```
PLAT242_ALERT_2_B Low    'MainMol' Ueq as Compared to Neighbors of      S2_13 Check
```

Author Response: The B level alert results from the high level of thermal motion round the NTf2 anions. The C level alerts of this type result from the generally high level of thermal motion throughout the structure.

```
PLAT260_ALERT_2_B Large Average Ueq of Residue Including      S1_9      0.342 Check
```

Author Response: The B level alert results from the high level of thermal motion round the NTf2 anions. The C level alerts of this type result from the generally high level of thermal motion throughout the structure.

```
PLAT260_ALERT_2_B Large Average Ueq of Residue Including     S1_12      0.406 Check
```

Author Response: The B level alert results from the high level of thermal motion round the NTf2 anions. The C level alerts of this type result from the generally high level of thermal motion throughout the structure.

```
PLAT260_ALERT_2_B Large Average Ueq of Residue Including     S1_14      0.424 Check
```

Author Response: The B level alert results from the high level of thermal motion round the NTf2 anions. The C level alerts of this type result from the generally high level of thermal motion throughout the structure.

```
PLAT260_ALERT_2_B Large Average Ueq of Residue Including    S1_15A      0.390 Check
```

Author Response: The B level alert results from the high level of thermal motion round the NTf2 anions. The C level alerts of this type result from the generally high level of thermal motion throughout the structure.

```
PLAT260_ALERT_2_B Large Average Ueq of Residue Including     O1_18      0.328 Check
```

Author Response: The B level alert results from the high level of thermal motion round the NTf2 anions. The C level alerts of this type result from the generally high level of thermal motion throughout the structure.

```
PLAT341_ALERT_3_B Low Bond Precision on  C-C Bonds ...............    0.01993 Ang.
```

Author Response: The flexibility of the modelled structure is reflected in the low precision of C-C bonds.

```
PLAT431_ALERT_2_B Short Inter HL..A Contact  Br71_4   ..O26_5    .       2.89 Ang.
                                                    x,y,z  =      1_555 Check
```

Author Response: This short intramolecular contact is in between the Br71_4 of ligand (RESI 4 FIT) and the oxygen atom O26_5 of ligand (RESI 5 FIT) or the Br70_6 of ligand (RESI 6 FIT) and the oxygen atom O1_12 of NTf2 (RESI 12 NTF). As inidicated by large displacement parameters in this structure, which results in higher uncertainty in the atomic positions and this short contact.

```
PLAT431_ALERT_2_B Short Inter HL..A Contact  Br70_6   ..O1_12    .       3.02 Ang.
                                               1-x,y,3/2-z  =      2_656 Check
```

Author Response: This short intramolecular contact is in between the Br71_4 of ligand (RESI 4 FIT) and the oxygen atom O26_5 of ligand (RESI 5 FIT) or the Br70_6 of ligand (RESI 6 FIT) and the oxygen atom O1_12 of NTf2 (RESI 12 NTF). As inidicated by large displacement parameters in this structure, which results in higher uncertainty in the atomic positions and this short contact.

Alert level C

```
ABSTY02_ALERT_1_C  An _exptl_absorpt_correction_type has been given without
            a literature citation. This should be contained in the
            _exptl_absorpt_process_details field.
            Absorption correction given as empirical
PLAT082_ALERT_2_C High R1 Value ..................................        0.12 Report
PLAT221_ALERT_2_C Solv./Anion  Resd11  O   Ueq(max)/Ueq(min) Range         7.1 Ratio
PLAT221_ALERT_2_C Solv./Anion  Resd11  S   Ueq(max)/Ueq(min) Range         4.3 Ratio
PLAT241_ALERT_2_C High   'MainMol' Ueq as Compared to Neighbors of       N1_7 Check
```

Author Response: The B level alert results from the high level of thermal motion round the NTf2 anions. The C level alerts of this type result from the generally high level of thermal motion throughout the structure.

```
PLAT241_ALERT_2_C High   'MainMol' Ueq as Compared to Neighbors of       C1_8 Check
```

Author Response: The B level alert results from the high level of thermal motion round the NTf2 anions. The C level alerts of this type result from the generally high level of thermal motion throughout the structure.

```
PLAT241_ALERT_2_C High   'MainMol' Ueq as Compared to Neighbors of       C2_8 Check
```

Author Response: The B level alert results from the high level of thermal motion round the NTf2 anions. The C level alerts of this type result from the generally high level of thermal motion throughout the structure.

```
PLAT241_ALERT_2_C High   'MainMol' Ueq as Compared to Neighbors of      C1_10 Check
```

Author Response: The B level alert results from the high level of thermal motion round the NTf2 anions. The C level alerts of this type result from the generally high level of thermal motion throughout the structure.

```
PLAT241_ALERT_2_C High   'MainMol' Ueq as Compared to Neighbors of      C2_11 Check
```

Author Response: The B level alert results from the high level of thermal motion round the NTf2 anions. The C level alerts of this type result from the generally high level of thermal motion throughout the structure.

```
PLAT241_ALERT_2_C High   'MainMol' Ueq as Compared to Neighbors of      C2_13 Check
```

Author Response: The B level alert results from the high level of thermal motion round the NTf2 anions. The C level alerts of this type result from the generally high level of thermal motion throughout the structure.

PLAT242_ALERT_2_C Low 'MainMol' Ueq as Compared to Neighbors of O28_4 Check

Author Response: The B level alert results from the high level of thermal motion round the NTf2 anions. The C level alerts of this type result from the generally high level of thermal motion throughout the structure.

PLAT242_ALERT_2_C Low 'MainMol' Ueq as Compared to Neighbors of C65_3 Check

Author Response: The B level alert results from the high level of thermal motion round the NTf2 anions. The C level alerts of this type result from the generally high level of thermal motion throughout the structure.

PLAT242_ALERT_2_C Low 'MainMol' Ueq as Compared to Neighbors of C5_4 Check

Author Response: The B level alert results from the high level of thermal motion round the NTf2 anions. The C level alerts of this type result from the generally high level of thermal motion throughout the structure.

PLAT242_ALERT_2_C Low 'MainMol' Ueq as Compared to Neighbors of C65_4 Check

Author Response: The B level alert results from the high level of thermal motion round the NTf2 anions. The C level alerts of this type result from the generally high level of thermal motion throughout the structure.

PLAT242_ALERT_2_C Low 'MainMol' Ueq as Compared to Neighbors of C5_6 Check

Author Response: The B level alert results from the high level of thermal motion round the NTf2 anions. The C level alerts of this type result from the generally high level of thermal motion throughout the structure.

PLAT242_ALERT_2_C Low 'MainMol' Ueq as Compared to Neighbors of S1_7 Check

Author Response: The B level alert results from the high level of thermal motion round the NTf2 anions. The C level alerts of this type result from the generally high level of thermal motion throughout the structure.

PLAT242_ALERT_2_C Low 'MainMol' Ueq as Compared to Neighbors of S2_7 Check

Author Response: The B level alert results from the high level of thermal motion round the NTf2 anions. The C level alerts of this type result from the generally high level of thermal motion throughout the structure.

```
PLAT242_ALERT_2_C Low    'MainMol' Ueq as Compared to Neighbors of       S2_9 Check
```

Author Response: The B level alert results from the high level of thermal motion round the NTf2 anions. The C level alerts of this type result from the generally high level of thermal motion throughout the structure.

```
PLAT242_ALERT_2_C Low    'MainMol' Ueq as Compared to Neighbors of      N1_10 Check
```

Author Response: The B level alert results from the high level of thermal motion round the NTf2 anions. The C level alerts of this type result from the generally high level of thermal motion throughout the structure.

```
PLAT242_ALERT_2_C Low    'MainMol' Ueq as Compared to Neighbors of      S2_11 Check
```

Author Response: The B level alert results from the high level of thermal motion round the NTf2 anions. The C level alerts of this type result from the generally high level of thermal motion throughout the structure.

```
PLAT242_ALERT_2_C Low    'MainMol' Ueq as Compared to Neighbors of      N1_13 Check
```

Author Response: The B level alert results from the high level of thermal motion round the NTf2 anions. The C level alerts of this type result from the generally high level of thermal motion throughout the structure.

```
PLAT260_ALERT_2_C Large Average Ueq of Residue Including      S1_7      0.173 Check
```

Author Response: The B level alert results from the high level of thermal motion round the NTf2 anions. The C level alerts of this type result from the generally high level of thermal motion throughout the structure.

```
PLAT260_ALERT_2_C Large Average Ueq of Residue Including      S1_8      0.257 Check
```

Author Response: The B level alert results from the high level of thermal motion round the NTf2 anions. The C level alerts of this type result from the generally high level of thermal motion throughout the structure.

```
PLAT260_ALERT_2_C Large Average Ueq of Residue Including     S1_10      0.216 Check
```

Author Response: The B level alert results from the high level of thermal motion round the NTf2 anions. The C level alerts of this type result from the generally high level of thermal motion throughout the structure.

PLAT260_ALERT_2_C Large Average Ueq of Residue Including S1_11 0.215 Check

Author Response: The B level alert results from the high level of thermal motion round the NTf2 anions. The C level alerts of this type result from the generally high level of thermal motion throughout the structure.

PLAT260_ALERT_2_C Large Average Ueq of Residue Including S1_13 0.252 Check

Author Response: The B level alert results from the high level of thermal motion round the NTf2 anions. The C level alerts of this type result from the generally high level of thermal motion throughout the structure.

PLAT260_ALERT_2_C Large Average Ueq of Residue Including S1_16B 0.137 Check

Author Response: The B level alert results from the high level of thermal motion round the NTf2 anions. The C level alerts of this type result from the generally high level of thermal motion throughout the structure.

PLAT260_ALERT_2_C Large Average Ueq of Residue Including O1_17 0.210 Check

Author Response: The B level alert results from the high level of thermal motion round the NTf2 anions. The C level alerts of this type result from the generally high level of thermal motion throughout the structure.

PLAT260_ALERT_2_C Large Average Ueq of Residue Including O1_19 0.187 Check

Author Response: The B level alert results from the high level of thermal motion round the NTf2 anions. The C level alerts of this type result from the generally high level of thermal motion throughout the structure.

PLAT260_ALERT_2_C Large Average Ueq of Residue Including O1_20 0.274 Check

Author Response: The B level alert results from the high level of thermal motion round the NTf2 anions. The C level alerts of this type result from the generally high level of thermal motion throughout the structure.

PLAT260_ALERT_2_C Large Average Ueq of Residue Including N1_21 0.172 Check

Author Response: The B level alert results from the high level of thermal motion round the NTf2 anions. The C level alerts of this type result from the generally high level of thermal motion throughout the structure.

```
PLAT260_ALERT_2_C Large Average Ueq of Residue Including     N1_22      0.242 Check
```

Author Response: The B level alert results from the high level of thermal motion round the NTf2 anions. The C level alerts of this type result from the generally high level of thermal motion throughout the structure.

```
PLAT260_ALERT_2_C Large Average Ueq of Residue Including     N1_23      0.276 Check
```

Author Response: The B level alert results from the high level of thermal motion round the NTf2 anions. The C level alerts of this type result from the generally high level of thermal motion throughout the structure.

```
PLAT260_ALERT_2_C Large Average Ueq of Residue Including     N1_24      0.246 Check
```

Author Response: The B level alert results from the high level of thermal motion round the NTf2 anions. The C level alerts of this type result from the generally high level of thermal motion throughout the structure.

```
PLAT360_ALERT_2_C Short  C(sp3)-C(sp3) Bond  C4_17    - C5_17    .       1.40 Ang.
PLAT431_ALERT_2_C Short Inter HL..A Contact  Br70_3   ..O30_4    .       3.16 Ang.
                                               2-x,1-y,1-z  =      3_766 Check
```

Author Response: This short intramolecular contact is in between the Br71_4 of ligand (RESI 4 FIT) and the oxygen atom O26_5 of ligand (RESI 5 FIT) or the Br70_6 of ligand (RESI 6 FIT) and the oxygen atom O1_12 of NTf2 (RESI 12 NTF). As inidicated by large displacement parameters in this structure, which results in higher uncertainty in the atomic positions and this short contact.

```
PLAT431_ALERT_2_C Short Inter HL..A Contact  Br71_3   ..O1_14    .       3.09 Ang.
                                               2-x,y,3/2-z  =      2_756 Check
```

Author Response: This short intramolecular contact is in between the Br71_4 of ligand (RESI 4 FIT) and the oxygen atom O26_5 of ligand (RESI 5 FIT) or the Br70_6 of ligand (RESI 6 FIT) and the oxygen atom O1_12 of NTf2 (RESI 12 NTF). As inidicated by large displacement parameters in this structure, which results in higher uncertainty in the atomic positions and this short contact.

```
PLAT431_ALERT_2_C Short Inter HL..A Contact  Br72_4   ..O3_8     .       3.26 Ang.
                                               2-x,y,3/2-z  =      2_756 Check
```

Author Response: This short intramolecular contact is in between the Br71_4 of ligand (RESI 4 FIT) and the oxygen atom O26_5 of ligand (RESI 5 FIT) or the Br70_6 of ligand (RESI 6 FIT) and the oxygen atom O1_12 of NTf2 (RESI 12 NTF). As inidicated by large displacement parameters in this structure, which results in higher uncertainty in the atomic positions and this short contact.

```
PLAT431_ALERT_2_C Short Inter HL..A Contact  Br71_6   ..O26_3    .       3.11 Ang.
                                               2-x,y,3/2-z  =      2_756 Check
```

Author Response: This short intramolecular contact is in between the Br71_4 of ligand (RESI 4 FIT) and the oxygen atom O26_5 of ligand (RESI 5 FIT) or the Br70_6 of ligand (RESI 6 FIT) and the oxygen atom O1_12 of NTf2 (RESI 12 NTF). As inidicated by large displacement parameters in this structure, which results in higher uncertainty in the atomic positions and this short contact.

```
PLAT431_ALERT_2_C Short Inter HL..A Contact  F5_7     ..O2_8     .       2.84 Ang.
                                                     x,y,z  =      1_555 Check
```

Author Response: This short intramolecular contact is in between the Br71_4 of ligand (RESI 4 FIT) and the oxygen atom O26_5 of ligand (RESI 5 FIT) or the Br70_6 of ligand (RESI 6 FIT) and the oxygen atom O1_12 of NTf2 (RESI 12 NTF). As inidicated by large displacement parameters in this structure, which results in higher uncertainty in the atomic positions and this short contact.

Alert level G

```
PLAT002_ALERT_2_G Number of Distance or Angle Restraints on AtSite        453 Note
PLAT003_ALERT_2_G Number of Uiso or Uij Restrained non-H Atoms ...        442 Report
PLAT051_ALERT_1_G Mu(calc) and Mu(CIF) Ratio Differs from 1.0 by .       7.89 %
PLAT092_ALERT_4_G Check: Wavelength Given is not Cu,Ga,Mo,Ag,In Ka    0.68890 Ang.
PLAT142_ALERT_4_G s.u. on b - Axis Small or Missing .............    0.00020 Ang.
PLAT143_ALERT_4_G s.u. on c - Axis Small or Missing .............    0.00030 Ang.
PLAT172_ALERT_4_G The CIF-Embedded .res File Contains DFIX Records        101 Report
PLAT173_ALERT_4_G The CIF-Embedded .res File Contains DANG Records        143 Report
PLAT174_ALERT_4_G The CIF-Embedded .res File Contains FLAT Records         87 Report
PLAT178_ALERT_4_G The CIF-Embedded .res File Contains SIMU Records          1 Report
PLAT186_ALERT_4_G The CIF-Embedded .res File Contains ISOR Records          1 Report
PLAT187_ALERT_4_G The CIF-Embedded .res File Contains RIGU Records          1 Report
PLAT242_ALERT_2_G Low    'MainMol' Ueq as Compared to Neighbors of       C1_9 Check
```

Author Response: The B level alert results from the high level of thermal motion round the NTf2 anions. The C level alerts of this type result from the generally high level of thermal motion throughout the structure.

```
PLAT302_ALERT_4_G Anion/Solvent/Minor-Residue Disorder (Resd 11  )       100% Note
PLAT302_ALERT_4_G Anion/Solvent/Minor-Residue Disorder (Resd 12  )       100% Note
PLAT304_ALERT_4_G Non-Integer Number of Atoms in ..... (Resd 11  )       9.09 Check
PLAT304_ALERT_4_G Non-Integer Number of Atoms in ..... (Resd 12  )       5.91 Check
PLAT344_ALERT_2_G Unusual sp?      Angle Range in Solvent/Ion for      C2_24 Check
PLAT398_ALERT_2_G Deviating  C-O-C   Angle From 120 for O16_3    .      106.5 Degree
PLAT398_ALERT_2_G Deviating  C-O-C   Angle From 120 for O33_3    .      105.5 Degree
PLAT398_ALERT_2_G Deviating  C-O-C   Angle From 120 for O52_3    .      106.1 Degree
PLAT398_ALERT_2_G Deviating  C-O-C   Angle From 120 for O16_4    .      106.1 Degree
PLAT398_ALERT_2_G Deviating  C-O-C   Angle From 120 for O33_4    .      106.9 Degree
PLAT398_ALERT_2_G Deviating  C-O-C   Angle From 120 for O52_4    .      106.2 Degree
```

```
PLAT398_ALERT_2_G Deviating  C-O-C   Angle From 120 for O16_5    .      106.1 Degree
PLAT398_ALERT_2_G Deviating  C-O-C   Angle From 120 for O33_5    .      105.1 Degree
PLAT398_ALERT_2_G Deviating  C-O-C   Angle From 120 for O52_5    .      106.8 Degree
PLAT398_ALERT_2_G Deviating  C-O-C   Angle From 120 for O16_6    .      105.6 Degree
PLAT398_ALERT_2_G Deviating  C-O-C   Angle From 120 for O33_6    .      106.1 Degree
PLAT398_ALERT_2_G Deviating  C-O-C   Angle From 120 for O52_6    .      106.8 Degree
PLAT398_ALERT_2_G Deviating  C-O-C   Angle From 120 for O1_20    .      106.4 Degree
PLAT431_ALERT_2_G Short Inter HL..A Contact  Br70_6   ..N1_12    .       3.29 Ang.
                                                    x,y,z  =      1_555 Check
```

Author Response: This short intramolecular contact is in between the Br71_4 of ligand (RESI 4 FIT) and the oxygen atom O26_5 of ligand (RESI 5 FIT) or the Br70_6 of ligand (RESI 6 FIT) and the oxygen atom O1_12 of NTf2 (RESI 12 NTF). As inidicated by large displacement parameters in this structure, which results in higher uncertainty in the atomic positions and this short contact.

```
PLAT432_ALERT_2_G Short Inter X...Y Contact  F6_8     ..C29_6    .       2.88 Ang.
                                                  x,1+y,z  =      1_565 Check
PLAT432_ALERT_2_G Short Inter X...Y Contact  O1_8     ..C48_6    .       2.99 Ang.
                                              x,1-y,1/2+z  =      4_566 Check
PLAT432_ALERT_2_G Short Inter X...Y Contact  O4_9     ..C3_21    .       2.79 Ang.
                                                    x,y,z  =      1_555 Check
PLAT432_ALERT_2_G Short Inter X...Y Contact  O4_9     ..C2_21    .       2.99 Ang.
                                                    x,y,z  =      1_555 Check
PLAT432_ALERT_2_G Short Inter X...Y Contact  O4_11    ..C44_6    .       3.01 Ang.
                                                    x,y,z  =      1_555 Check
PLAT432_ALERT_2_G Short Inter X...Y Contact  O1_13    ..C44_4    .       2.90 Ang.
                                              2-x,y,3/2-z  =      2_756 Check
PLAT432_ALERT_2_G Short Inter X...Y Contact  O3_15A   ..C7_3     .       2.80 Ang.
                                                    x,y,z  =      1_555 Check
PLAT432_ALERT_2_G Short Inter X...Y Contact  O3_16B   ..C2_4     .       2.99 Ang.
                                               2-x,-y,1-z  =      3_756 Check
PLAT432_ALERT_2_G Short Inter X...Y Contact  O3_16B   ..C68_3    .       3.00 Ang.
                                              x,-y,-1/2+z  =      4_555 Check
PLAT432_ALERT_2_G Short Inter X...Y Contact  O3_16B   ..C67_3    .       3.02 Ang.
                                              x,-y,-1/2+z  =      4_555 Check
PLAT432_ALERT_2_G Short Inter X...Y Contact  O4_16B   ..C7_3     .       2.86 Ang.
                                                    x,y,z  =      1_555 Check
PLAT434_ALERT_2_G Short Inter HL..HL Contact Br72_4   ..F4_8     .       3.03 Ang.
                                              2-x,y,3/2-z  =      2_756 Check
PLAT434_ALERT_2_G Short Inter HL..HL Contact Br70_5   ..F5_7     .       2.98 Ang.
                                                    x,y,z  =      1_555 Check
PLAT434_ALERT_2_G Short Inter HL..HL Contact Br72_5   ..F3_9     .       3.15 Ang.
                                             x,1-y,-1/2+z  =      4_565 Check
PLAT434_ALERT_2_G Short Inter HL..HL Contact F5_7     ..F1_8     .       2.74 Ang.
                                                    x,y,z  =      1_555 Check
PLAT606_ALERT_4_G Solvent Accessible VOID(S) in Structure ........          ! Info
PLAT720_ALERT_4_G Number of Unusual/Non-Standard Labels ..........        661 Note
PLAT790_ALERT_4_G Centre of Gravity not Within Unit Cell: Resd.  #          6 Note
              C2 F6 N O4 S2
PLAT790_ALERT_4_G Centre of Gravity not Within Unit Cell: Resd.  #          9 Note
              C2 F6 N O4 S2
PLAT790_ALERT_4_G Centre of Gravity not Within Unit Cell: Resd.  #         15 Note
              C4 H10 O
PLAT790_ALERT_4_G Centre of Gravity not Within Unit Cell: Resd.  #         18 Note
```

```
              C2 H3 N
PLAT794_ALERT_5_G Tentative Bond Valency for Fe1_1     (III)     .       3.82 Info
PLAT794_ALERT_5_G Tentative Bond Valency for Fe2_1     (III)     .       3.76 Info
PLAT794_ALERT_5_G Tentative Bond Valency for Fe1_2     (III)     .       3.68 Info
PLAT794_ALERT_5_G Tentative Bond Valency for Fe2_2     (III)     .       3.85 Info
PLAT860_ALERT_3_G Number of Least-Squares Restraints .............       7318 Note
PLAT869_ALERT_4_G ALERTS Related to the Use of SQUEEZE  Suppressed          ! Info
PLAT883_ALERT_1_G No Info/Value for _atom_sites_solution_primary .     Please Do !
PLAT984_ALERT_1_G The Br-f'=   -0.2901 Deviates from the B&C-Value    -0.1853 Check
PLAT984_ALERT_1_G The Fe-f'=    0.3463 Deviates from the B&C-Value     0.3429 Check
PLAT984_ALERT_1_G The  S-f'=    0.1246 Deviates from the B&C-Value     0.1187 Check
PLAT985_ALERT_1_G The Br-f"=    2.4595 Deviates from the B&C-Value     2.3459 Check
PLAT985_ALERT_1_G The Fe-f"=    0.8444 Deviates from the B&C-Value     0.8028 Check
PLAT985_ALERT_1_G The  S-f"=    0.1234 Deviates from the B&C-Value     0.1169 Check
```

---

 1 **ALERT level A** = Most likely a serious problem - resolve or explain
17 **ALERT level B** = A potentially serious problem, consider carefully
40 **ALERT level C** = Check. Ensure it is not caused by an omission or oversight
66 **ALERT level G** = General information/check it is not something unexpected

 9 ALERT type 1 CIF construction/syntax error, inconsistent or missing data
86 ALERT type 2 Indicator that the structure model may be wrong or deficient
 5 ALERT type 3 Indicator that the structure quality may be low
20 ALERT type 4 Improvement, methodology, query or suggestion
 4 ALERT type 5 Informative message, check

It is advisable to attempt to resolve as many as possible of the alerts in all categories. Often the minor alerts point to easily fixed oversights, errors and omissions in your CIF or refinement strategy, so attention to these fine details can be worthwhile. In order to resolve some of the more serious problems it may be necessary to carry out additional measurements or structure refinements. However, the purpose of your study may justify the reported deviations and the more serious of these should normally be commented upon in the discussion or experimental section of a paper or in the "special_details" fields of the CIF. checkCIF was carefully designed to identify outliers and unusual parameters, but every test has its limitations and alerts that are not important in a particular case may appear. Conversely, the absence of alerts does not guarantee there are no aspects of the results needing attention. It is up to the individual to critically assess their own results and, if necessary, seek expert advice.

Publication of your CIF in IUCr journals

A basic structural check has been run on your CIF. These basic checks will be run on all CIFs submitted for publication in IUCr journals (*Acta Crystallographica*, *Journal of Applied Crystallography*, *Journal of Synchrotron Radiation*); however, if you intend to submit to *Acta Crystallographica Section C* or *E* or *IUCrData*, you should make sure that full publication checks are run on the final version of your CIF prior to submission.

Publication of your CIF in other journals

Please refer to the *Notes for Authors* of the relevant journal for any special instructions relating to CIF submission.

PLATON version of 28/11/2022; check.def file version of 28/11/2022

Datablock xwc399_sq - ellipsoid plot

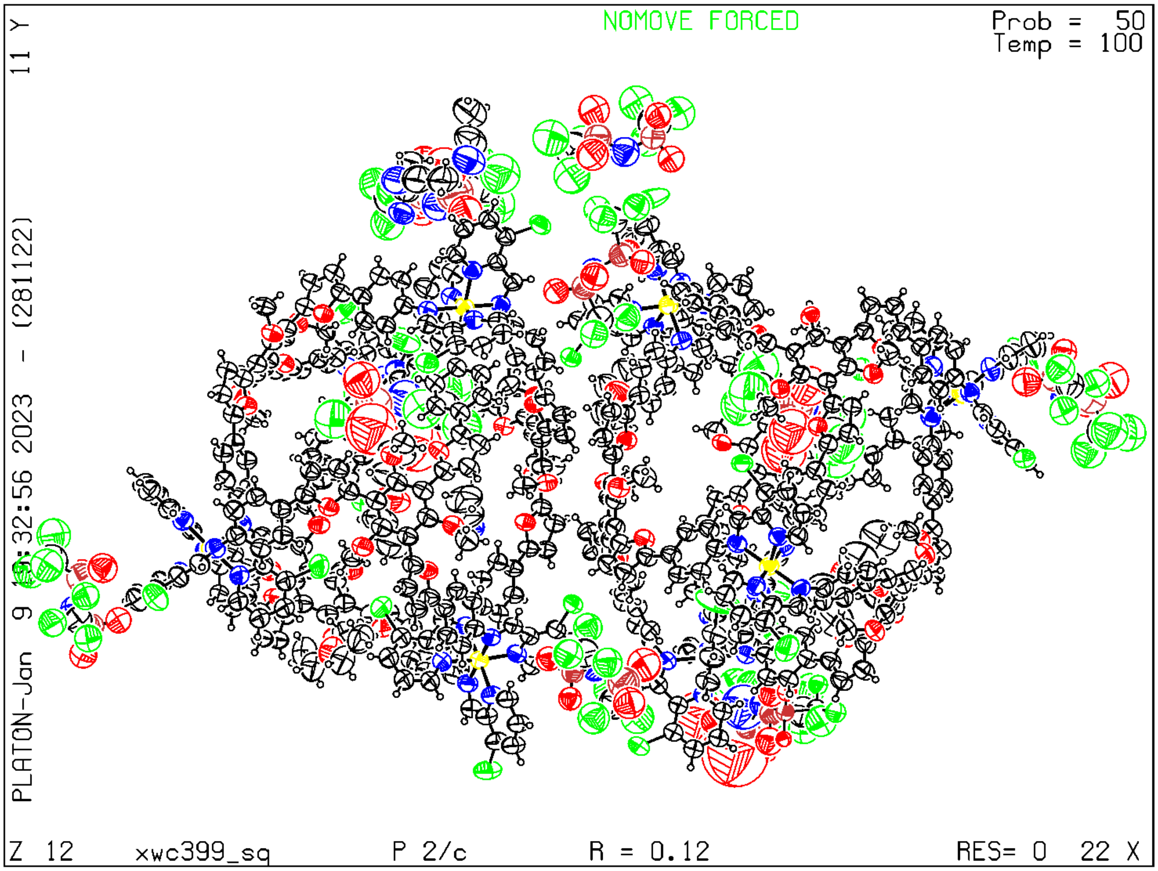

Supplement: Supplementary file 2 — Supporting Information [file ANIE-62-0-s001.pdf]
