# Supplementary material for: Allosterically Regulated Guest Binding Determines Framework Symmetry for an FeII 4L4 Cage
Source: Angew Chem Int Ed Engl. 2023 Mar 28;62(18):e202301319. doi: 10.1002/anie.202301319 (PMC10947561; doi:10.1002/anie.202301319)
Supplement: Supplementary file 3 — Supporting Information [file ANIE-62-0-s003.pdf]

Angewandte Chemie
Eine Zeitschrift der Gesellschaft Deutscher Chemiker

Supporting Information

Allosterically Regulated Guest Binding Determines Framework Symmetry for an $Fe^{II}_4L_4$ Cage

*W. Xue, K. Wu, N. Ouyang, T. Brotin, J. R. Nitschke**

Supporting Information

Table of Contents

1 General Information S3

2 Synthesis and Characterization of Subcomponent **A** S4

3 Self-Assembly and Characterization of $Fe^{II}_4L_4$ and $Fe^{II}_3L_2$ S6

3.1 Self-Assembly of Cage **1** S6

3.2 Self-Assembly of Sandwich **2** S13

3.3 Self-Assembly of Cages **3−5** S16

4 Conversion of Sandwich **2** to Cage **1** S24

5 Host-Guest Properties of **1** S25

5.1 Host-Guest Interaction of **1** with **G1** and **G2** S26

5.2 Host-Guest Interaction of **1** with **G3** and **G3’** S31

5.3 Host-Guest Interaction of **1** with **G4** S39

5.4 Host-Guest Interaction of **G3⊂1** and **G4⊂1** with **G1** S45

5.5 Host-Guest Interaction of **(G1)$_x$⊂1** with **G3** and **G4** S49

5.6 Host-Guest Interaction of **G3⊂1** and **G3·(G1)$_x$⊂1** with **G4** S51

6 Volume Calculations S53

7 Crystal Structure of **3** S54

8 References S58

1 General Information

Unless otherwise specified, all reagents were purchased from commercial sources and used as received. Precursor **S1** was prepared according to a reported procedure.[1] *MM*-cryptophane (**G3**) and *PP*-cryptophane (**G3'**) were synthesized according to a reported procedure and resolved by HPLC.[2] $Fe(NTf_2)_2 \cdot 4.5H_2O$ was prepared according to a reported procedure.[3] Self-assembly reactions were performed in either CD_3CN or distilled MeCN.

NMR spectra were recorded using the following NMR spectrometers: Bruker 400 MHz Avance III HD smart probe (^{1}H, ^{13}C, ^{19}F, ^{31}P,^{1}H-DOSY, and 2D NMR), Bruker 500 MHz AVIII HD Smart Probe (^{1}H and ^{19}F), Bruker Avance 500 MHz DCH cryoprobe (^{1}H, ^{13}C, and 2D NMR). Chemical shifts of the NMR spectra are reported relative to $CDCl_3$ (^{1}H NMR: δ = 7.26 ppm, ^{13}C NMR: δ = 77.0 ppm), CD_3CN (^{1}H NMR: δ = 1.94 ppm, ^{13}C NMR: δ = 118.3 ppm). Data for ^{1}H NMR spectra were reported as follows: chemical shift (ppm), peak shape (s = singlet, d = doublet, t = triplet, m = multiplet, br = broad signal), coupling constant (Hz), and integration. Data for ^{13}C, ^{19}F and ^{31}P NMR are reported in terms of chemical shift (ppm). Data for ^{13}C NMR are reported with chemical shift (ppm) values referenced to the residual solvent peak; data for ^{19}F and ^{31}P NMR are reported with chemical shift (ppm) values as observed.

UV-vis measurements were employed to fine-tune the solution concentration for subsequent CD measurements, and were performed on a Varian Cary 400 scan UV-vis spectrophotometer with a 1 mm path-length cuvette at 25 °C. Circular Dichroism was performed on an Applied-Photophysics Chirascan CD spectrometer using a 1 mm path-length cuvette. Experiments were recorded at 298 K, maintained with a Peltier temperature control. Measurements were background subtracted from blank solvent in an identical cuvette. The sample concentrations were adjusted to maintain a HV below 800 V.

High resolution electrospray ionisation mass spectra (HR-ESI-MS) were recorded on a Waters Synapt G2-Si instrument.

2 Synthesis and Characterization of Subcomponent A

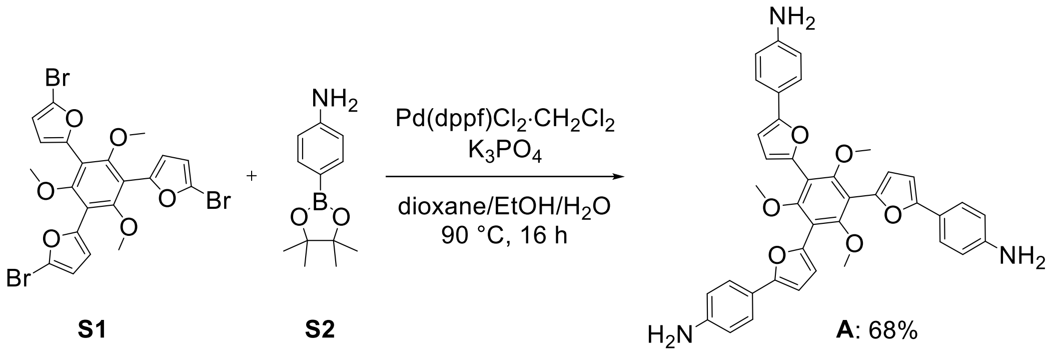

To a solution of 5,5',5''-(2,4,6-trimethoxybenzene-1,3,5-triyl)tris(2-bromofuran) (**S1**, 180.9 mg, 0.3 mmol, 1.0 equiv) and 4-aminophenylboronic acid pinacol ester (**S2**, 262.9 mg, 1.2 mmol, 4.0 equiv) in a mixed solvent of 1,4-dioxane (7 mL), EtOH (2 mL) and H_2O (1 mL), was added Pd(dppf)Cl_2·CH_2Cl_2 (73.5 mg, 0.09 mmol, 0.3 equiv) and K_3PO_4 (254.7 mg, 1.2 mmol, 4.0 equiv). The reaction mixture was refluxed at 90 °C for 16 hours under nitrogen. After cooling down to room temperature, CH_2Cl_2 (50 mL) was added, and the mixture was then filtrated through Celite. The organic phase was washed with brine (50 mL) and H_2O (2 x 50mL), and the combined aqueous phase was extracted with CH_2Cl_2 (2 x 50mL). The organic phases were combined, dried over anhydrous Na_2SO_4, and filtered. The solvent was removed under reduced pressure. The residual solid was purified by flash column chromatography filled with silica gel, using CH_2Cl_2/EtOAc = 90/10 as eluent, affording subcomponent **A** as a brown solid (130.5 mg, 68%).

R_f = 0.50 (CH_2Cl_2/EtOAc = 90/10). **^{1}H NMR** (400 MHz, $CDCl_3$): δ 3.57 (s, 9H), 3.73 (br, 6H), 6.605 (d, *J* = 3.3, 3H), 6.70 (d, *J* = 8.5 Hz, 6H), 6.725 (d, *J* = 3.3, 3H), 7.55 (d, *J* = 8.5 Hz, 6H) ppm. **^{13}C NMR** (100 MHz, $CDCl_3$): δ 61.4, 104.1, 113.2, 115.2, 116.4, 122.1, 125.1, 144.7, 145.8, 154.1, 157.9 ppm.

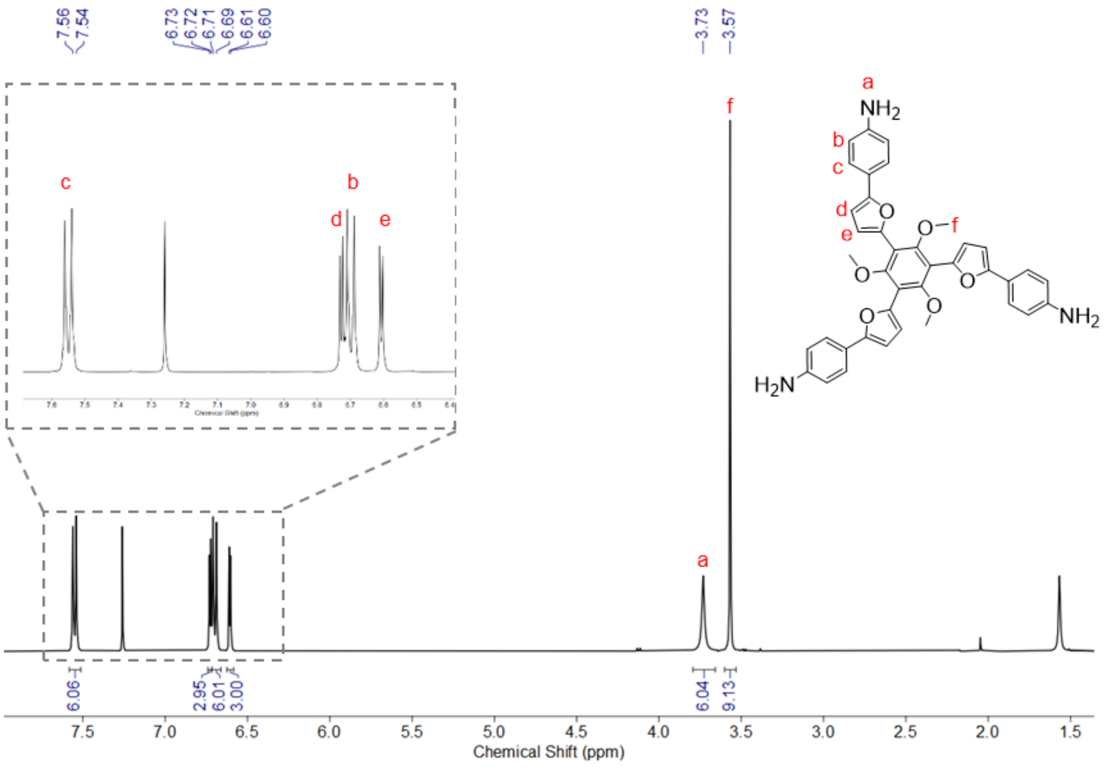

Figure S1. ^{1}H NMR spectrum of subcomponent **A** (400 MHz, $CDCl_3$, 25 °C).

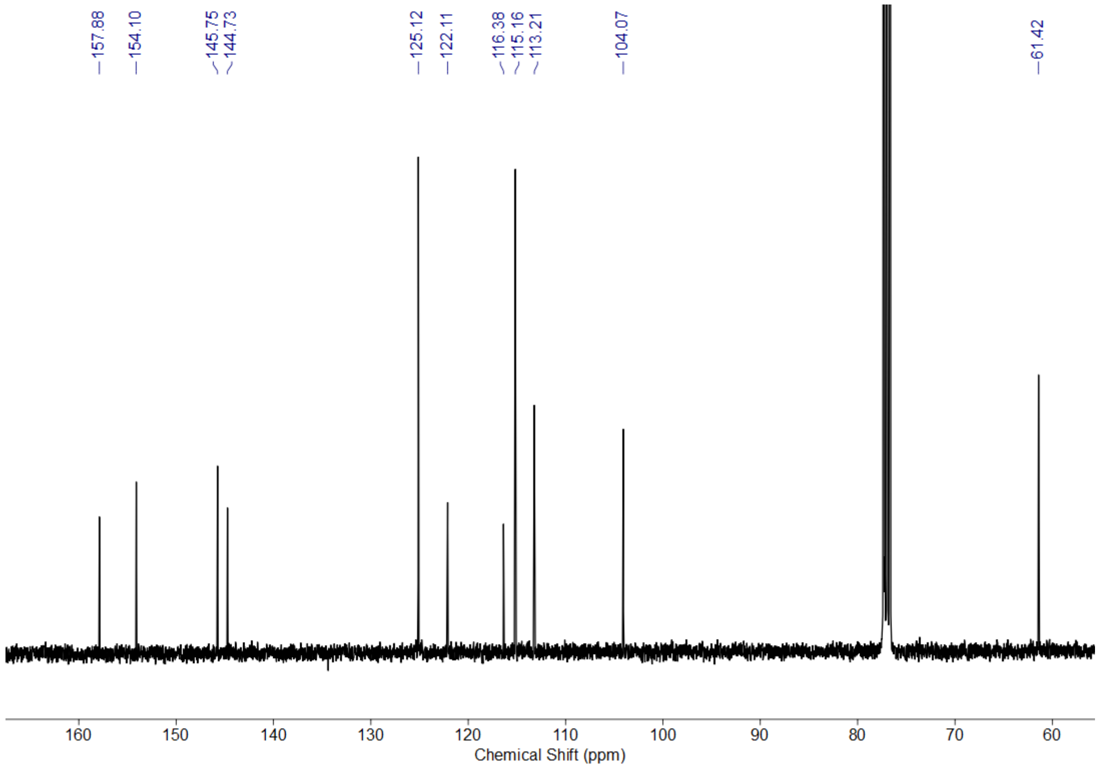

Figure S2. ^{13}C NMR spectrum of subcomponent **A** (100 MHz, $CDCl_3$, 25 °C).

3 Self-Assembly and Characterization of $Fe^{II}_4L_4$ and $Fe^{II}_3L_2$

3.1 Self-Assembly of Cage 1

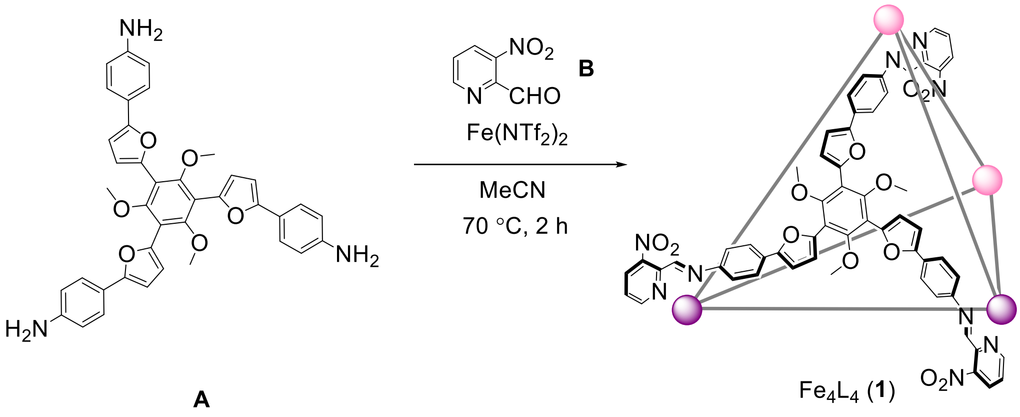

Subcomponent **A** (9.6 mg, 15.0 µmol, 1.0 equiv), 3-nitro-2-formylpyridine **B** (6.9 mg, 45.0 µmol, 3.0 equiv) and $Fe(NTf_2)_2·4.5H_2O$ (10.5 mg, 15.0 µmol, 1.0 equiv) were combined in MeCN (3 mL) in a 15 mL tube. The reaction mixture was stirred at 70 °C for 2 hours under nitrogen. The solvent was evaporated to around 0.5 mL, and Et_2O (15 mL) was then added. The precipitate was collected by centrifugation and washed with excess Et_2O, affording cage **1** as a purple solid (23.4 mg, 87%).

^{1}H NMR (400 MHz, CD_3CN): δ 10.002 (s, 4H), 9.509 (s, 4H), 9.151 (s, 4H), 8.945 (dd, *J* = 1.0, 8.7 Hz, 4H), 8.808 (d, *J* = 5.5 Hz, 4H), 8.652 (d, *J* = 5.5 Hz, 4H), 8.537 (dd, *J* = 1.0, 8.7 Hz, 4H), 8.503 (dd, *J* = 1.0, 8.7 Hz, 4H), 8.025 (dd, *J* = 5.5, 8.7 Hz, 4H), 7.986 (dd, *J* = 5.5, 8.7 Hz, 4H), 7.851 (d, *J* = 5.5 Hz, 4H), 7.728–7.834 (m, 20H), 7.695 (d, *J* = 8.6 Hz, 4H), 7.293 (d, *J* = 8.6 Hz, 8H), 7.167 (d, *J* = 3.5 Hz, 4H), 7.109 (d, *J* = 8.6 Hz, 4H), 7.010 (d, *J* = 8.6 Hz, 4H), 6.950 (d, *J* = 3.5 Hz, 4H), 6.856 (d, *J* = 3.5 Hz, 4H), 6.799(d, *J* = 3.5 Hz, 4H), 6.688 (d, *J* = 3.5 Hz, 4H), 6.651 (d, *J* = 3.5 Hz, 4H), 6.539 (d, *J* = 8.6 Hz, 8H), 6.340 (d, *J* = 8.6 Hz, 4H), 3.638 (s, 12H), 3.482 (s, 24H). **^{13}C NMR** (125 MHz, CD_3CN): δ 175.24, 172.10, 168.38, 161.69, 161.18, 159.71, 158.97, 158.89, 158.72, 153.03, 152.22, 151.99, 151.80, 151.57, 151.24, 150.13, 149.21, 147.96, 147.86, 147.36, 146.93, 145.78, 144.59, 136.78, 136.32, 136.20, 132.78, 132.25, 131.79, 131.54, 130.95, 129.90, 125.38, 125.23, 124.70, 124.56, 124.43,

124.14, 123.80, 123.02, 121.88, 116.60, 116.59, 115.99, 115.62, 114.89, 114.84, 114.49, 110.59, 110.18, 109.96, 62.61, 61.96, 61.84 ppm.

HR-ESI-MS: m/z = 548.8513 [**1**-8(NTf_2)]$^{8+}$, 667.2468 [**1**-7(NTf_2)]$^{7+}$, 825.2737 [**1**-6(NTf_2)]$^{6+}$, 1046.3129 [**1**-5(NTf_2)]$^{5+}$, 1377.8697 [**1**-4(NTf_2)]$^{4+}$, 1930.4709 [**1**-3(NTf_2)]$^{3+}$.

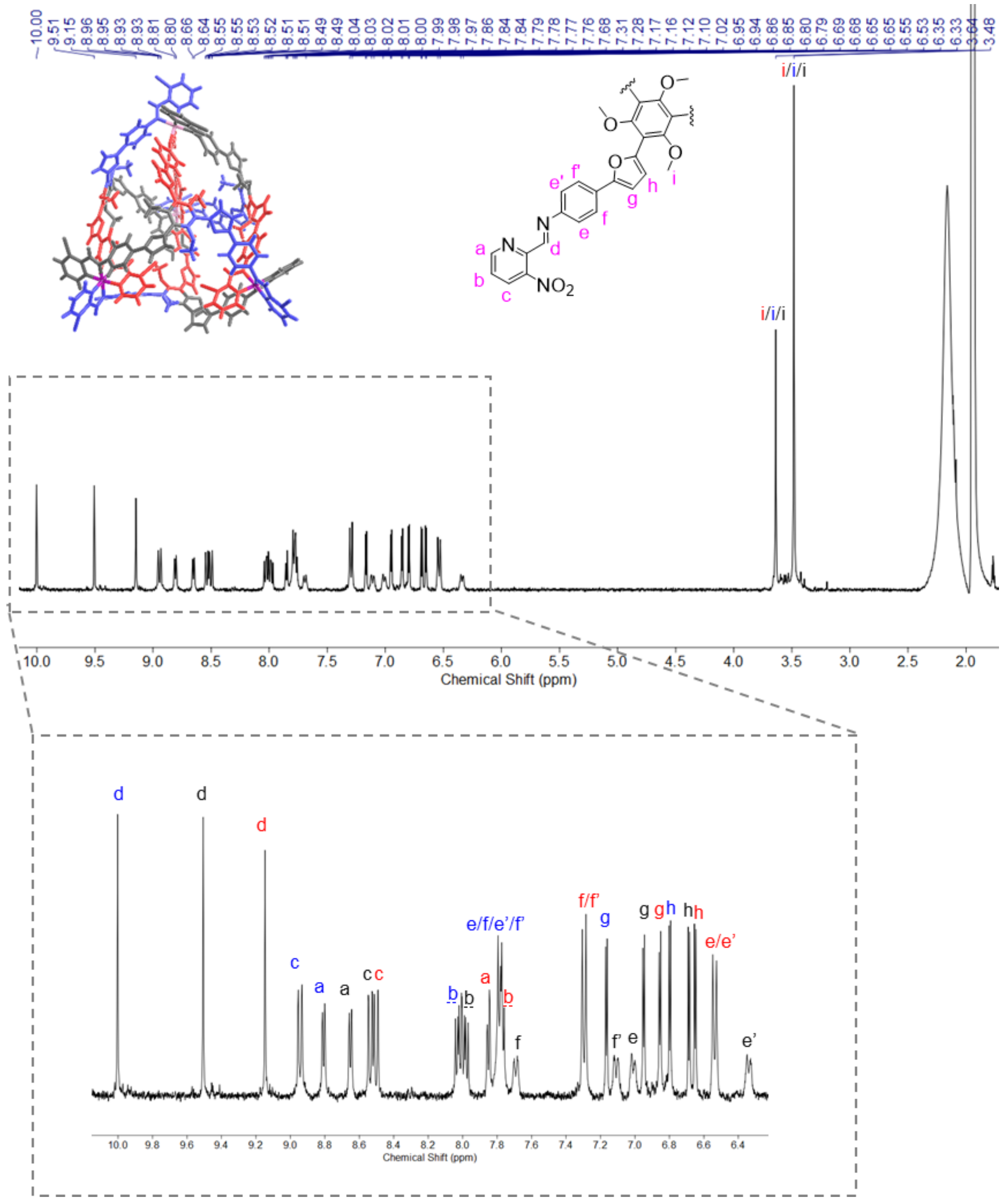

Figure S3. ^{1}H NMR spectrum of cage **1** (400 MHz, CD_3CN, 25 °C).

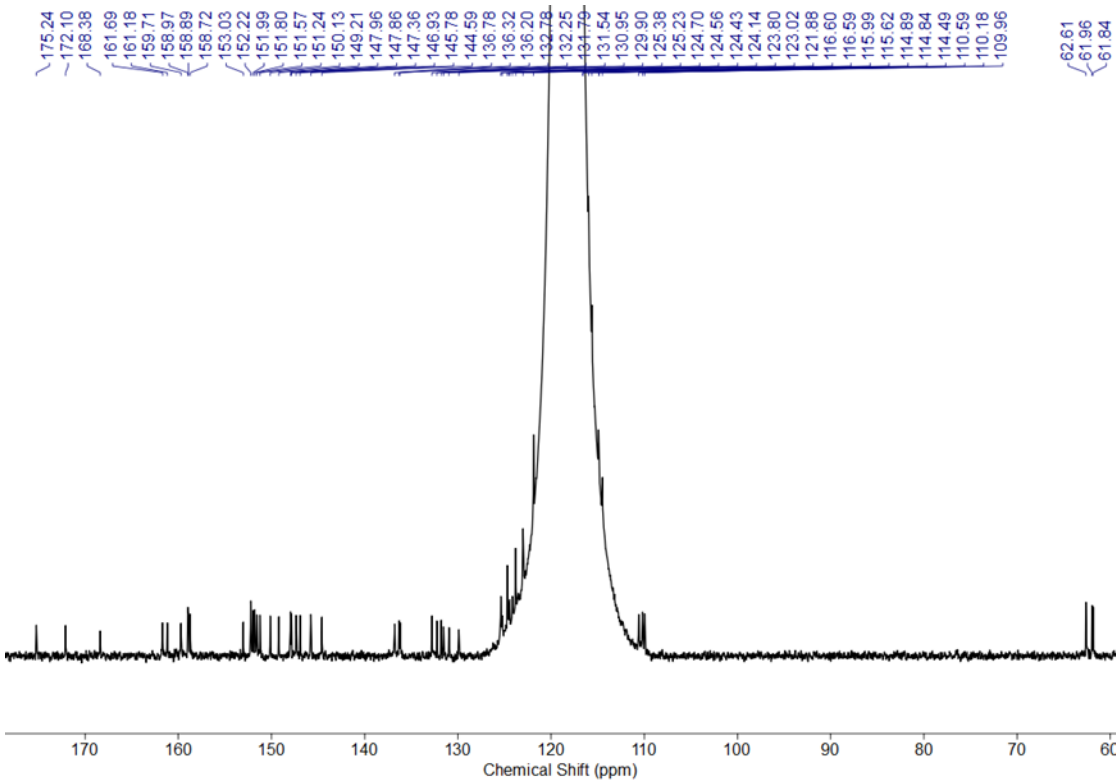

Figure S4. ^{13}C NMR spectrum of cage **1** (125 MHz, CD_3CN, 25 °C).

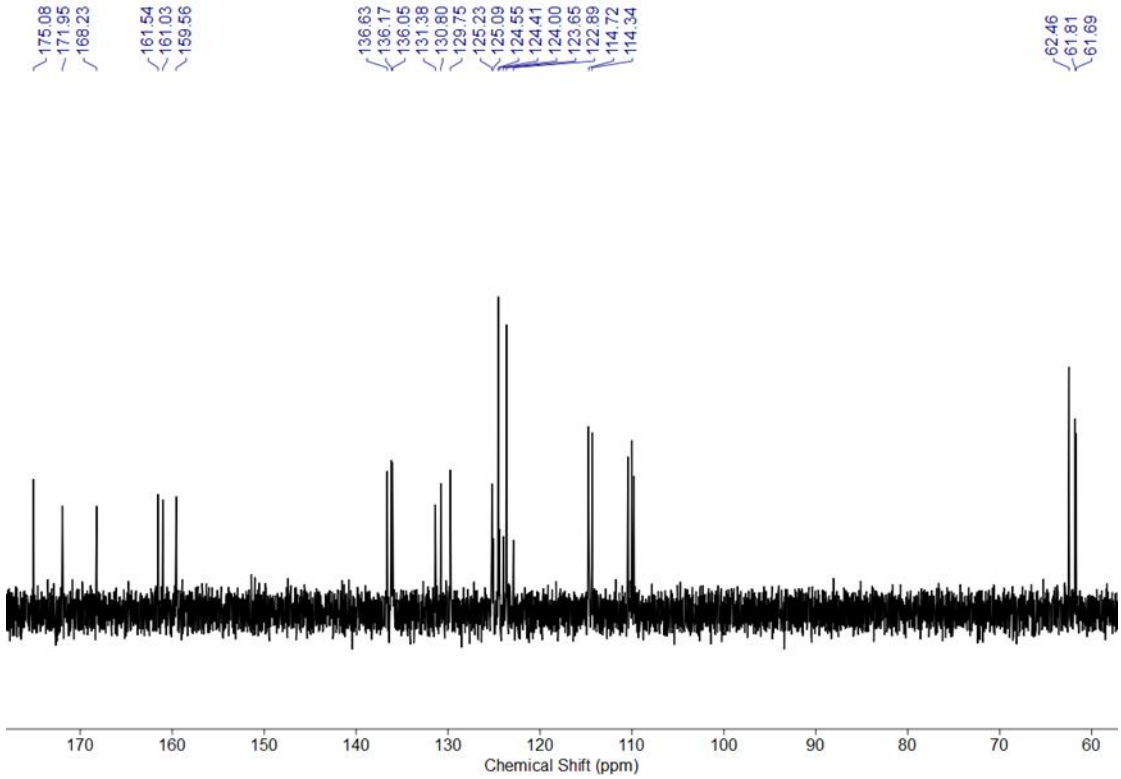

Figure S5. ^{13}C DEPT-135 NMR spectrum of cage **1** (125 MHz, CD_3CN, 25 °C).

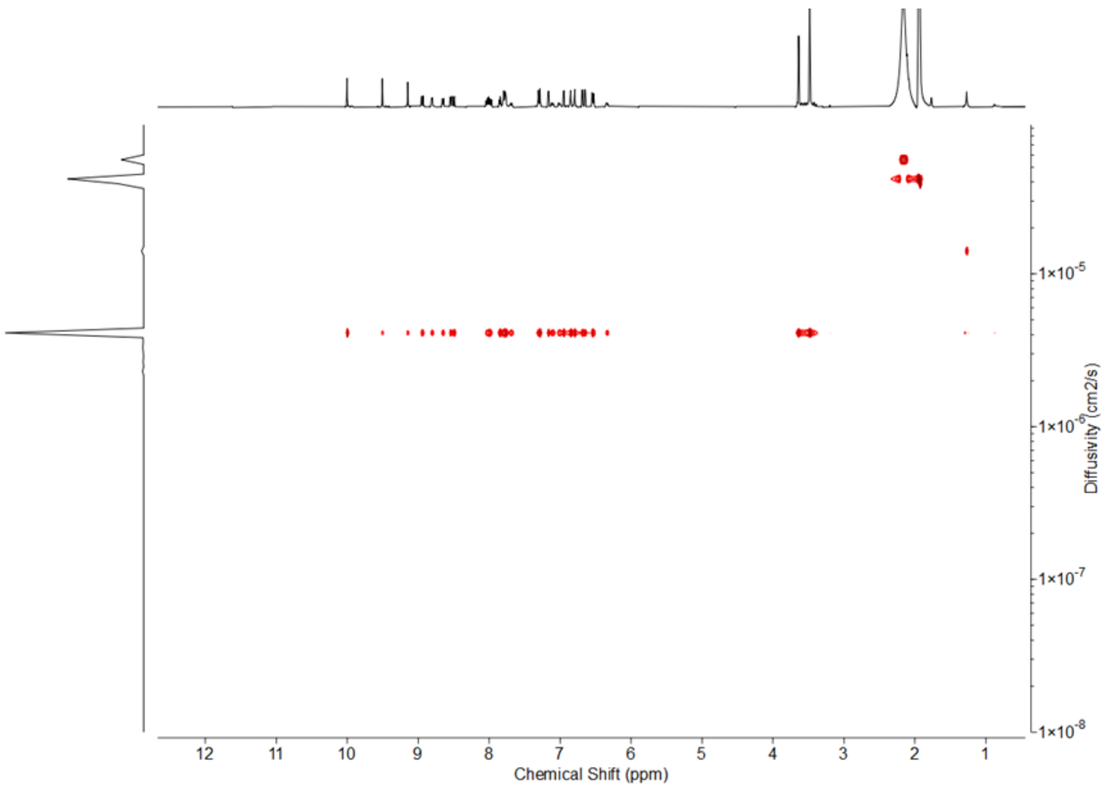

Figure S6. ^{1}H DOSY spectrum of cage **1** (400 MHz, CD_3CN, 25 °C). The diffusion coefficient was measured to be 4.10×10^{-6} cm^2/s.

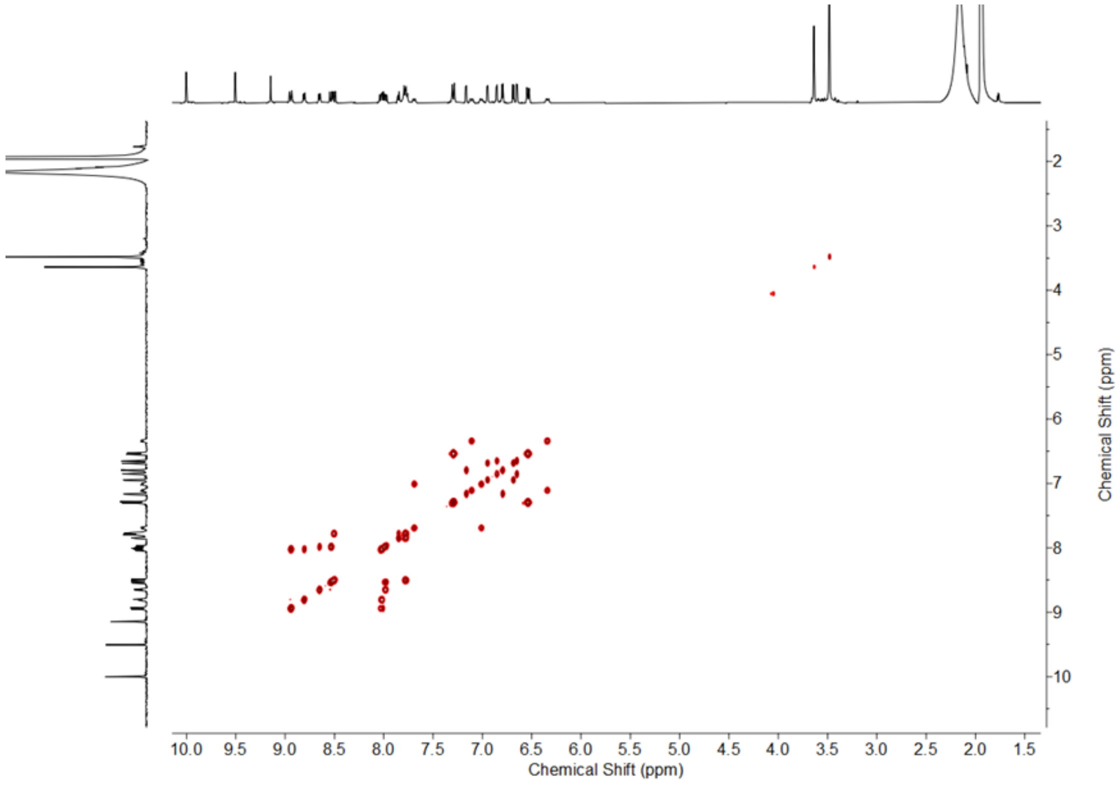

Figure S7. ^{1}H-^{1}H COSY NMR spectrum of cage **1** (500 MHz, CD_3CN, 25 °C).

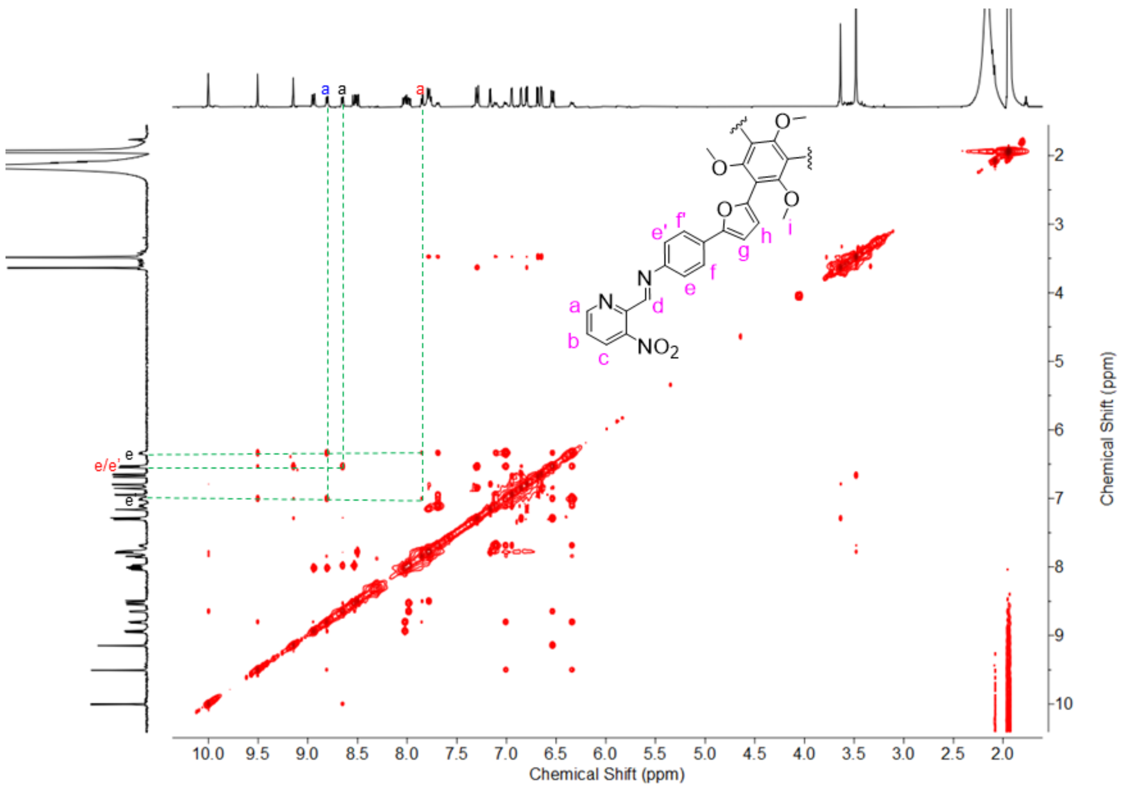

Figure S8. ^{1}H-^{1}H NOESY NMR spectrum of cage **1** (500 MHz, CD_3CN, 25 °C).

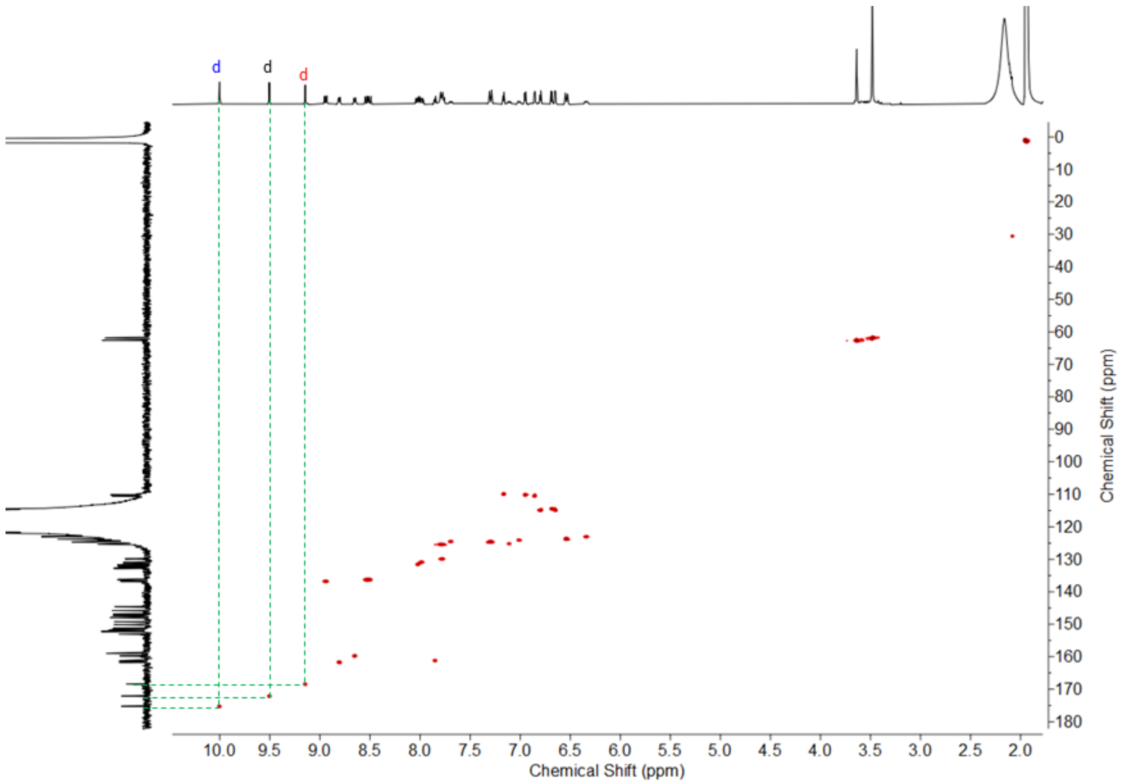

Figure S9. ^{1}H-^{13}C HSQC NMR spectrum of cage **1** (500 MHz, CD_3CN, 25 °C).

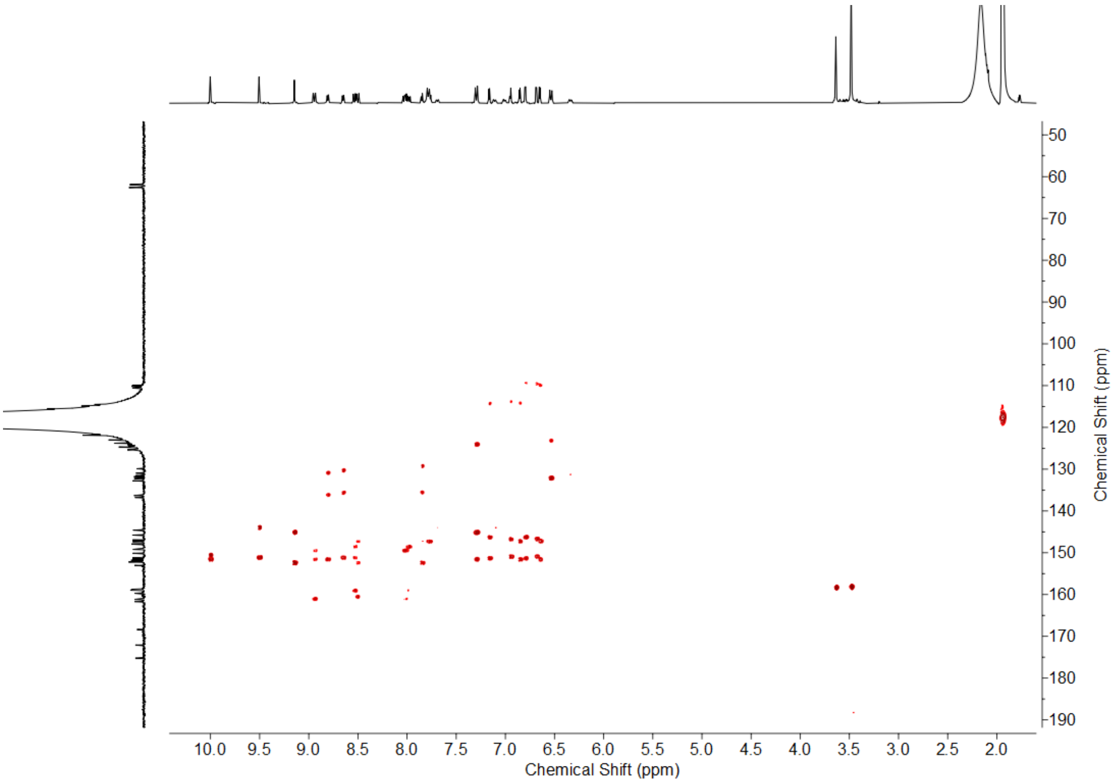

Figure S10. ^{1}H-^{13}C HMBC NMR spectrum of cage **1** (500 MHz, CD_3CN, 25 °C).

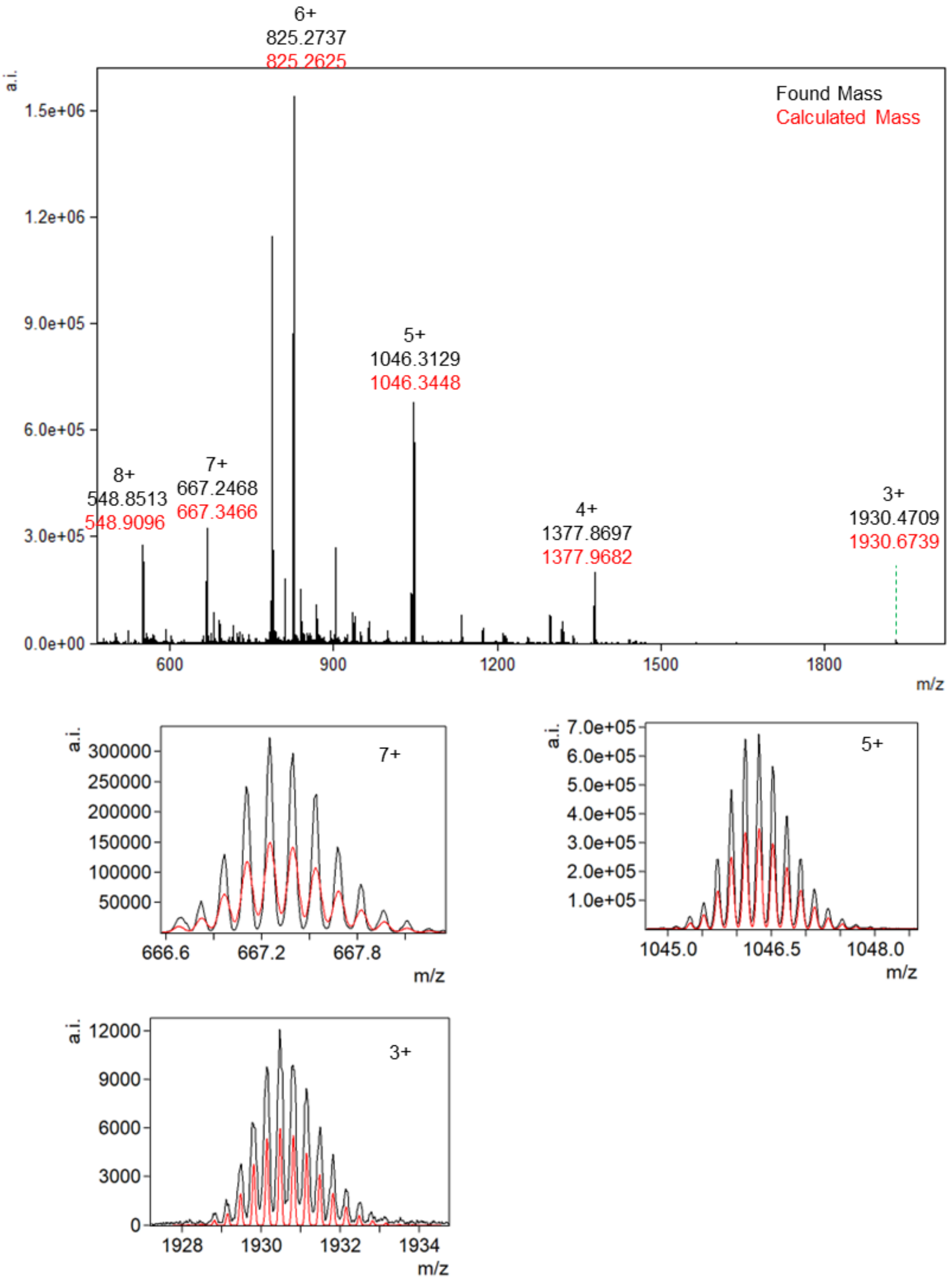

Figure S11. High-resolution ESI-MS spectrum of cage **1** in MeCN.

3.2 Self-Assembly of Sandwich 2

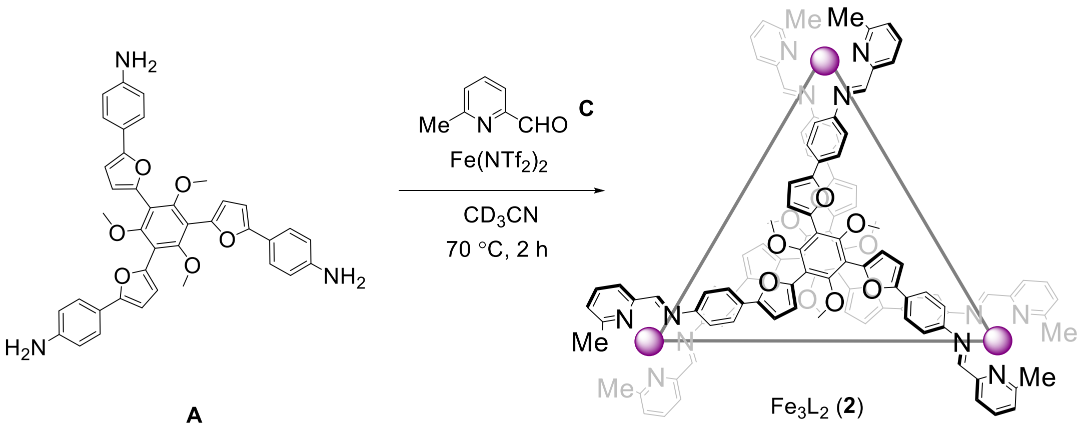

Subcomponent **A** (1.9 mg, 3.0 μmol, 1.0 equiv), 6-methyl-2-formylpyridine **C** (1.5 mg, 9.0 μmol, 3.0 equiv) and $Fe(NTf_2)_2{\cdot}4.5H_2O$ (3.2 mg, 4.5 μmol, 1.5 equiv) were combined in CD_3CN (0.5 mL) in a J Young NMR tube. The reaction mixture was stirred at 70 °C for 2 hours under nitrogen, affording a yellow solution of **2**. The NMR data was collected without purification. **Note:** Different ratios of $Fe(NTf_2)_2{\cdot}4.5H_2O$ and Subcomponents were attempted; in all cases, Fe_3L_2 was produced, without Fe_4L_4 being observed.

HR-ESI-MS: m/z = 469.1159 [**2**-5(NTf$_2$)]$^{5+}$, 656.3798 [**2**-4(NTf$_2$)]$^{4+}$, 968.4887 [**2**-3(NTf$_2$)]$^{3+}$, 1593.2024 [**2**-2(NTf$_2$)]$^{2+}$.

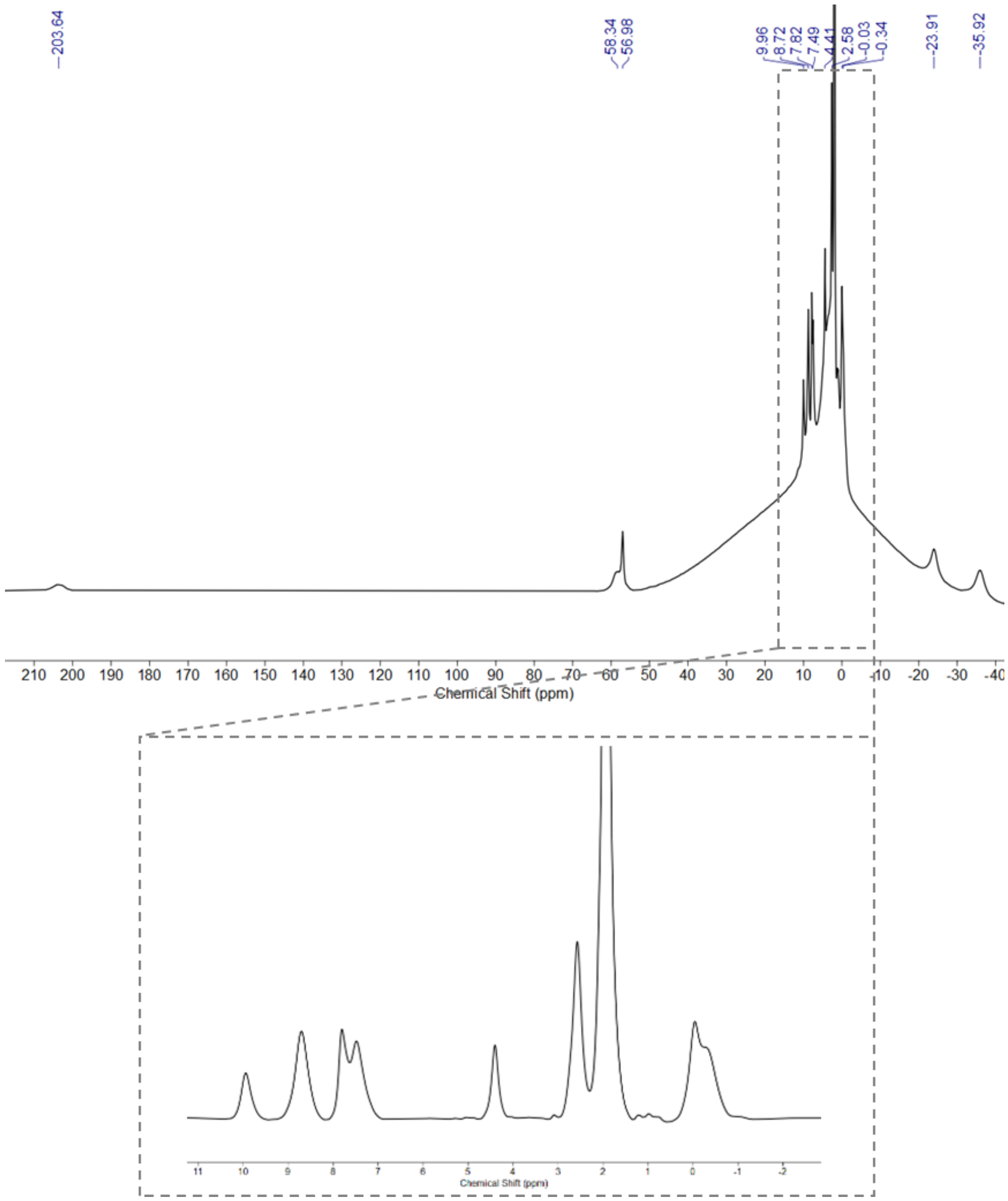

Figure S12. Wide sweep ^{1}H NMR spectrum of sandwich **2** (400 MHz, CD_3CN, 25 °C).

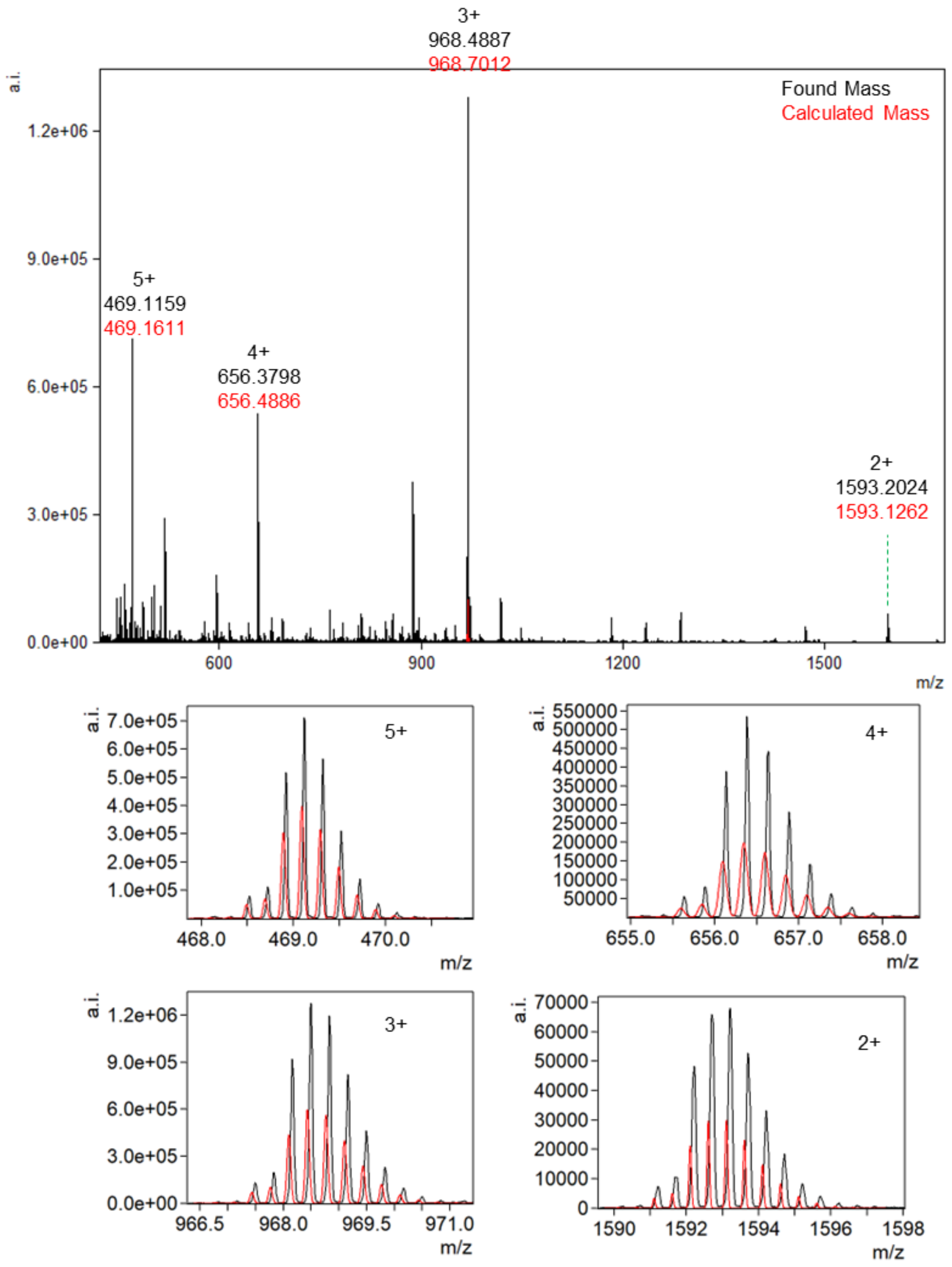

Figure S13. High-resolution ESI-MS spectrum of sandwich **2** in MeCN. Peaks corresponding to **2**·(MeCN)$_x$ were not observed under these experimental conditions.

3.3 Self-Assembly of Cages 3–5

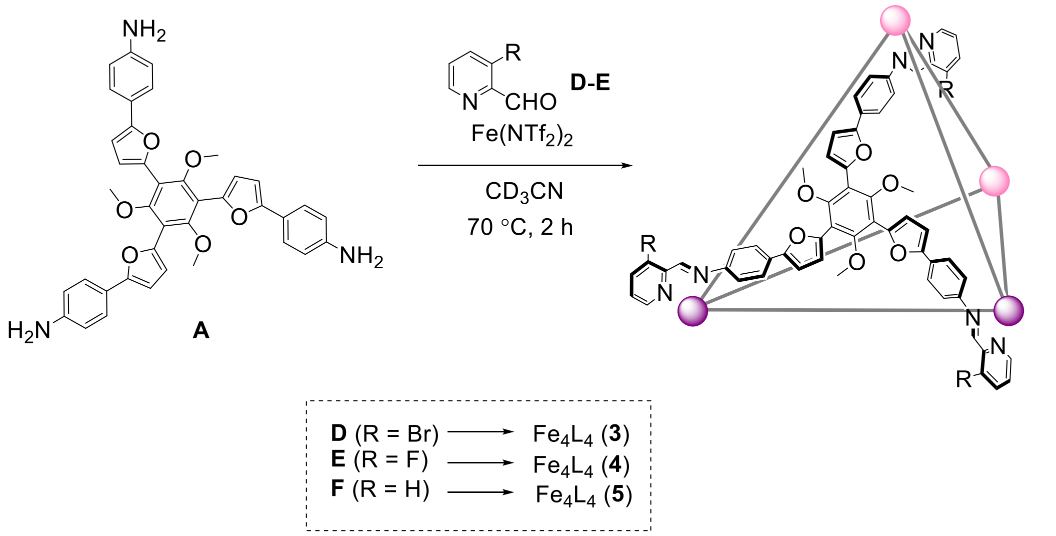

Subcomponent **A** (1.9 mg, 3.0 μmol, 1.0 equiv), 2-formylpyridine (9.0 μmol, 3.0 equiv) and $Fe(NTf_2)_2 \cdot 4.5H_2O$ (2.1 mg, 3.0 μmol, 1.0 equiv) were combined in CD_3CN (0.5 mL) in a J Young NMR tube. The reaction mixture was stirred at 70 °C for 2 hours under nitrogen, affording the CD_3CN solution of $Fe^{II}{}_4L_4$ cage (dark blue: **3**; brown: **4**; purple: **5**). The NMR data was collected without purification.

Three sets of proton signals observed in ^{1}H NMR spectrum indicate the formation of S_4-symmetric complex. Similar diffusion coefficients were observed in DOSY spectra, indicating that all cages have similar hydrodynamic radii. The $Fe^{II}{}_4L_4$ stoichiometry was confirmed by HR-ESI-MS.

Cage **3**:

HR-ESI-MS: m/z = 599.9879 [**1**-8(NTf$_2$)]$^{8+}$, 725.4022 [**1**-7(NTf$_2$)]$^{7+}$, 892.9553 [**1**-6(NTf$_2$)]$^{6+}$, 1127.7312 [**1**-5(NTf$_2$)]$^{5+}$, 1479.6406 [**1**-4(NTf$_2$)]$^{4+}$, 2066.1584 [**1**-3(NTf$_2$)]$^{3+}$.

Cage **4**:

HR-ESI-MS: m/z = 508.3574 [**1**-8(NTf$_2$)]$^{8+}$, 620.9677 [**1**-7(NTf$_2$)]$^{7+}$, 771.1145 [**1**-6(NTf$_2$)]$^{6+}$, 981.5225 [**1**-5(NTf$_2$)]$^{5+}$, 1296.6315 [**1**-4(NTf$_2$)]$^{4+}$, 1822.4788 [**1**-3(NTf$_2$)]$^{3+}$.

Cage **5**:

HR-ESI-MS: m/z = 481.3716 [**1**-8(NTf_2)]$^{8+}$, 590.1286 [**1**-7(NTf_2)]$^{7+}$, 735.3029 [**1**-6(NTf_2)]$^{6+}$, 938.3501 [**1**-5(NTf_2)]$^{5+}$, 1242.9175 [**1**-4(NTf_2)]$^{4+}$, 1750.5269 [**1**-3(NTf_2)]$^{3+}$.

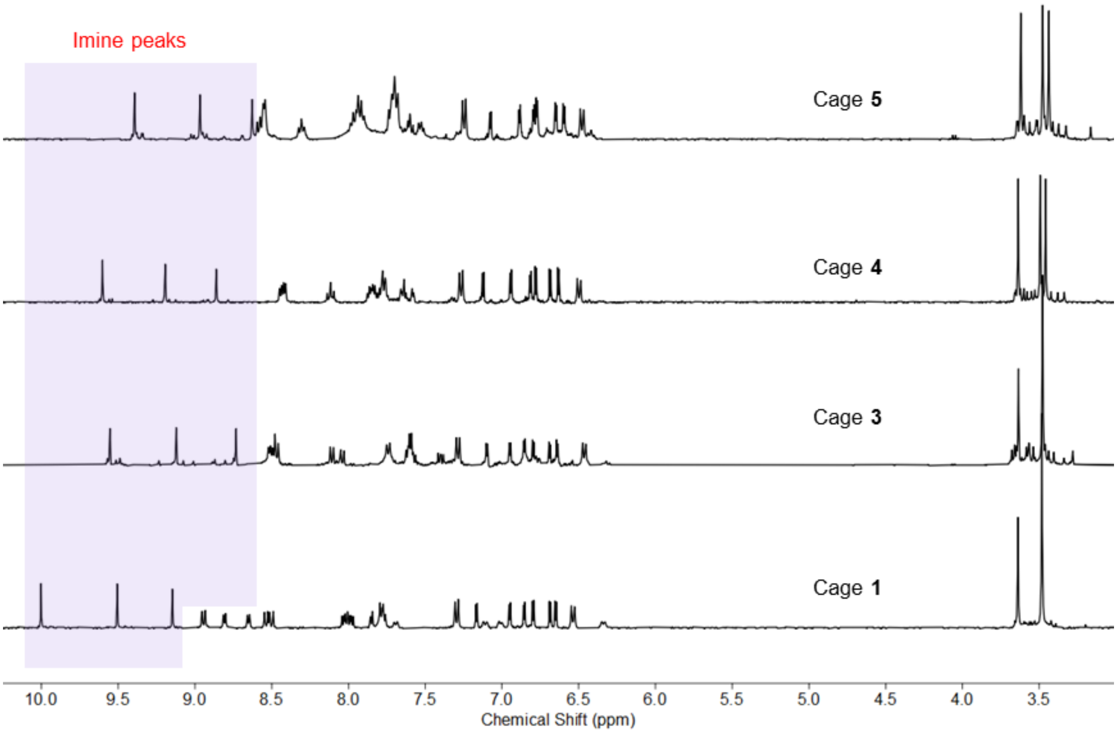

Figure S14. Comparison of ^1H NMR spectra of cages **1** and **3**–**5** (400 MHz, CD_3CN, 25 °C).

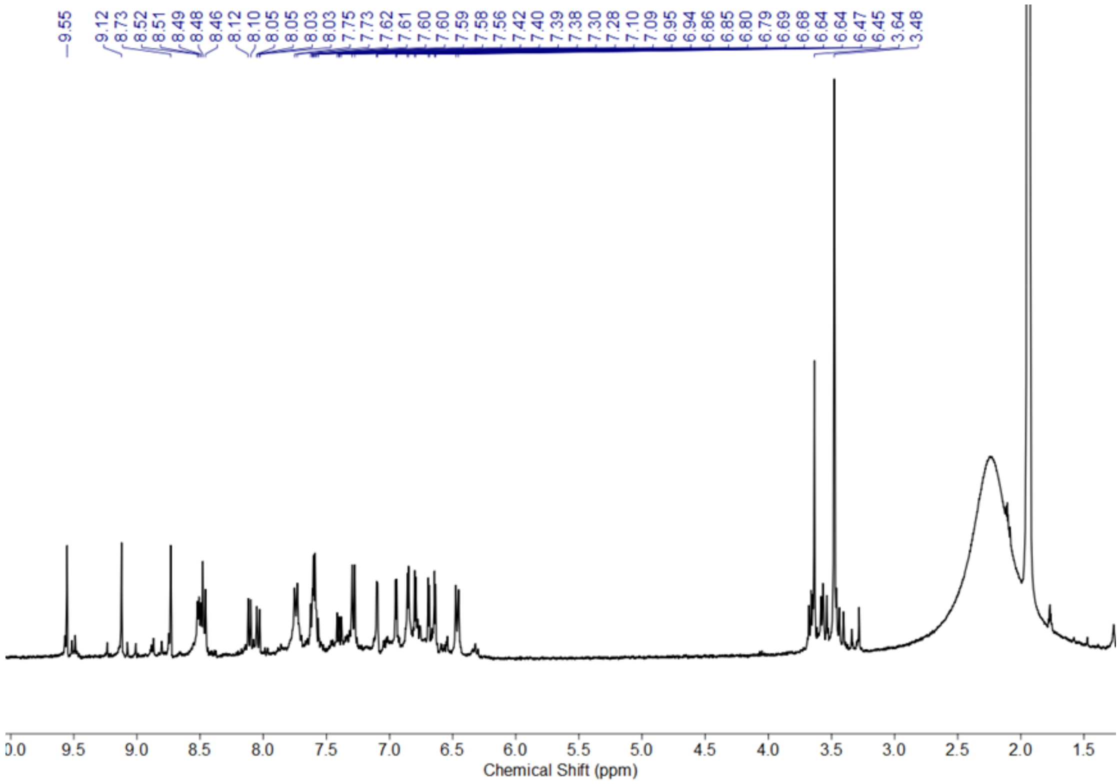

Figure S15. ^{1}H NMR spectrum of cage **3** (400 MHz, CD_3CN, 25 °C).

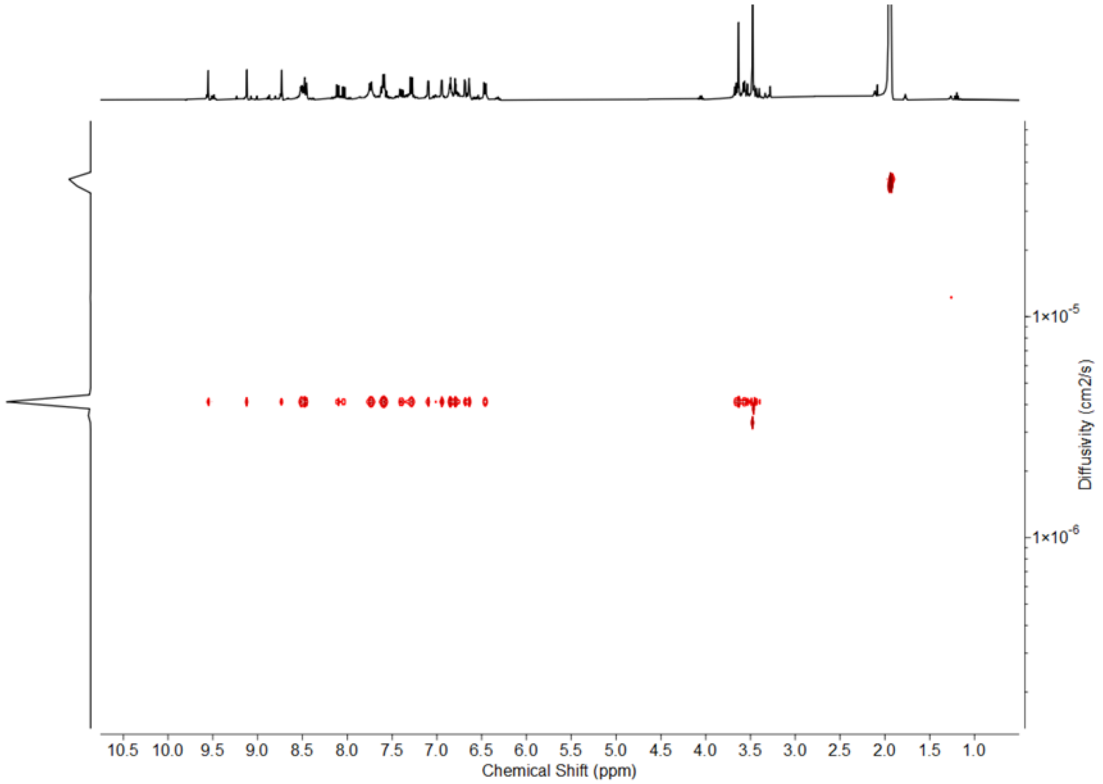

Figure S16. ^{1}H DOSY spectrum of cage **3** (400 MHz, CD_3CN, 25 °C). The diffusion coefficient was measured to be 4.09×10^{-6} cm^2/s.

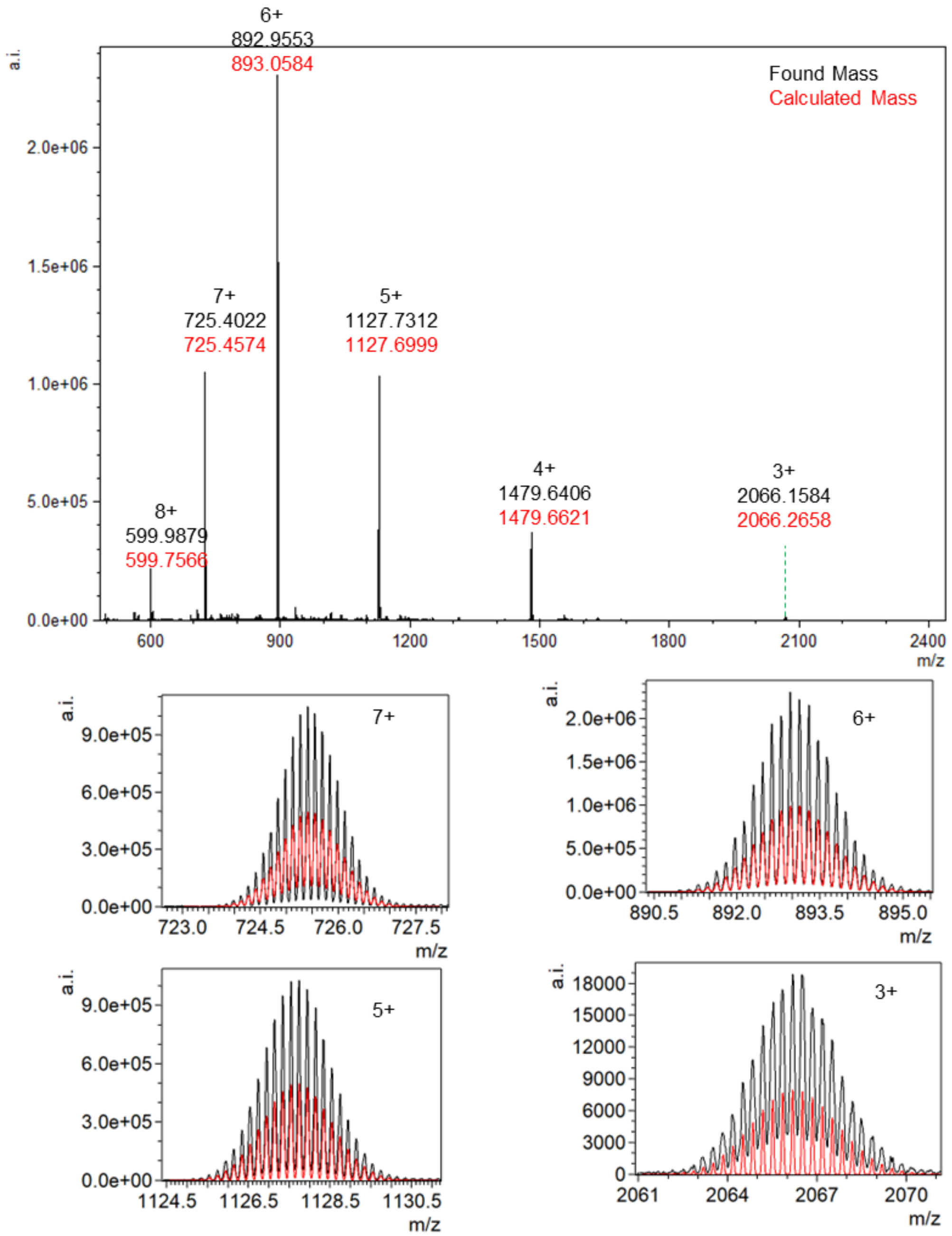

Figure S17. High-resolution ESI-MS spectrum of cage **3** in MeCN.

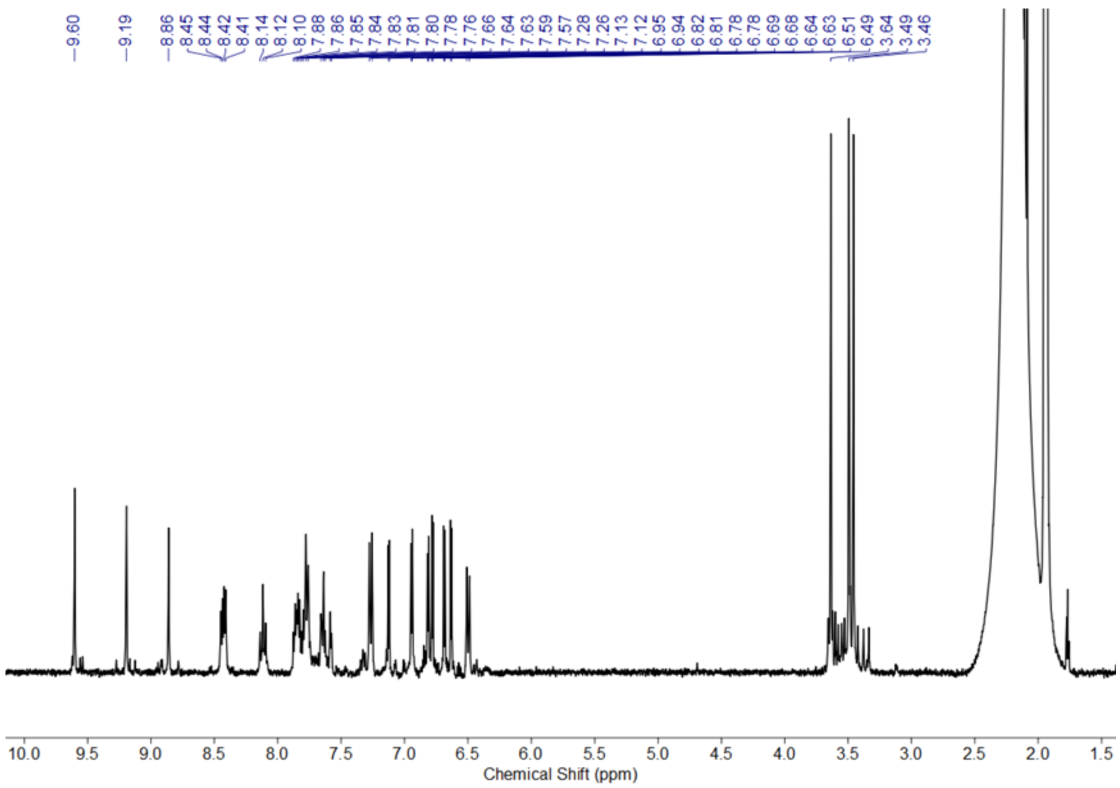

Figure S18. ^{1}H NMR spectrum of cage **4** (400 MHz, CD_3CN, 25 °C).

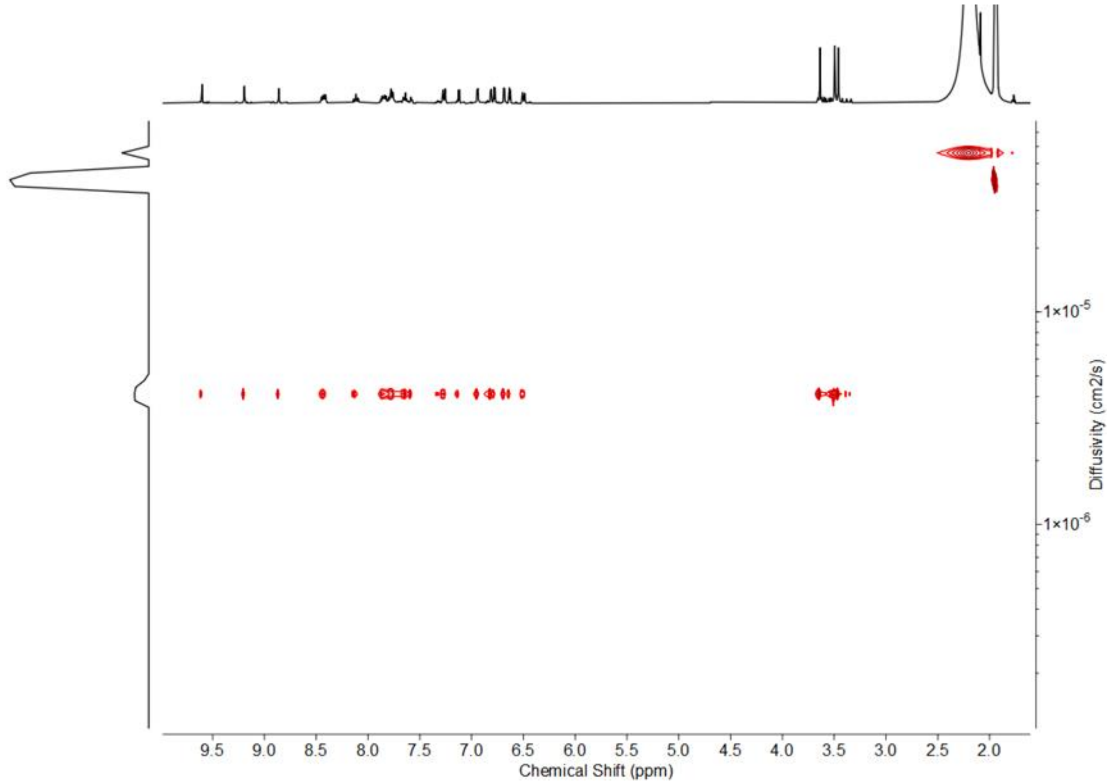

Figure S19. ^{1}H DOSY spectrum of cage **4** (400 MHz, CD_3CN, 25 °C). The diffusion coefficient was measured to be 4.10×10^{-6} cm^2/s.

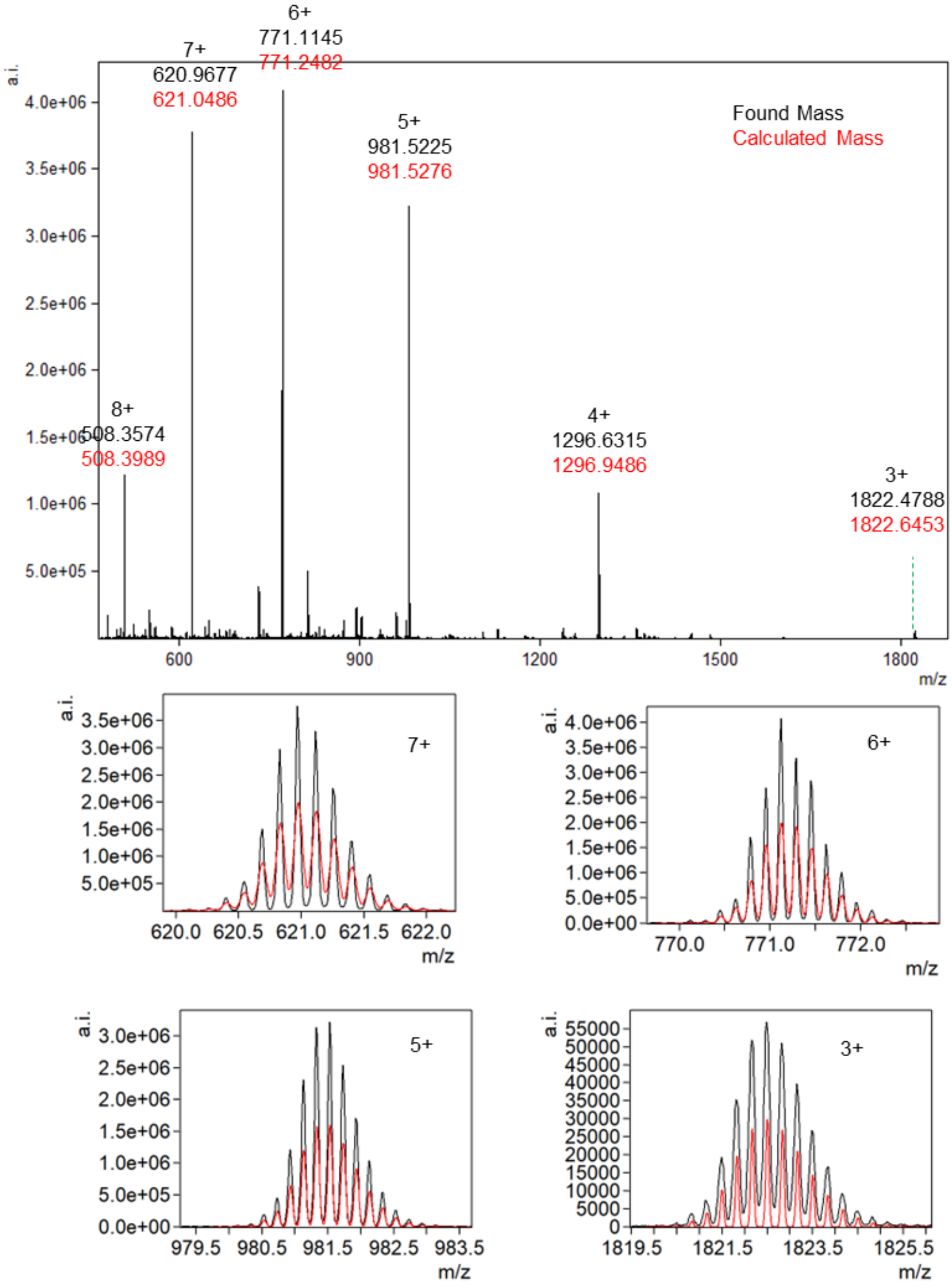

Figure S20. High-resolution ESI-MS spectrum of cage **4** in MeCN.

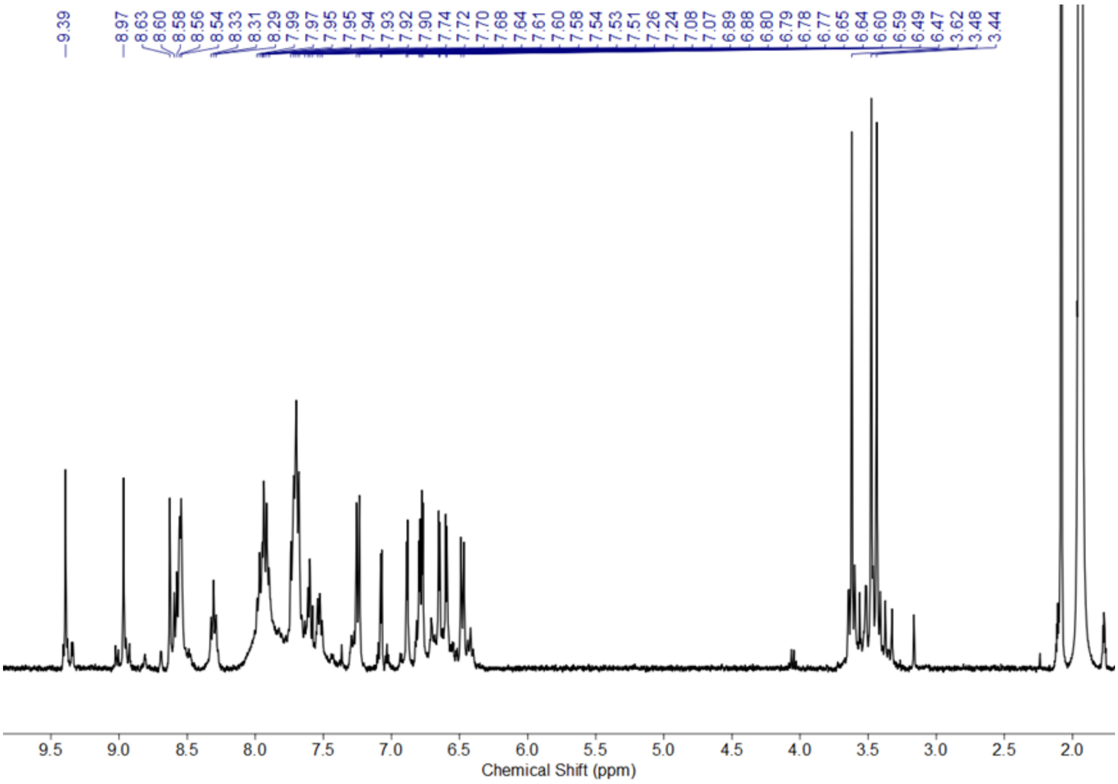

Figure S21. ^{1}H NMR spectrum of cage **5** (400 MHz, CD_3CN, 25 °C).

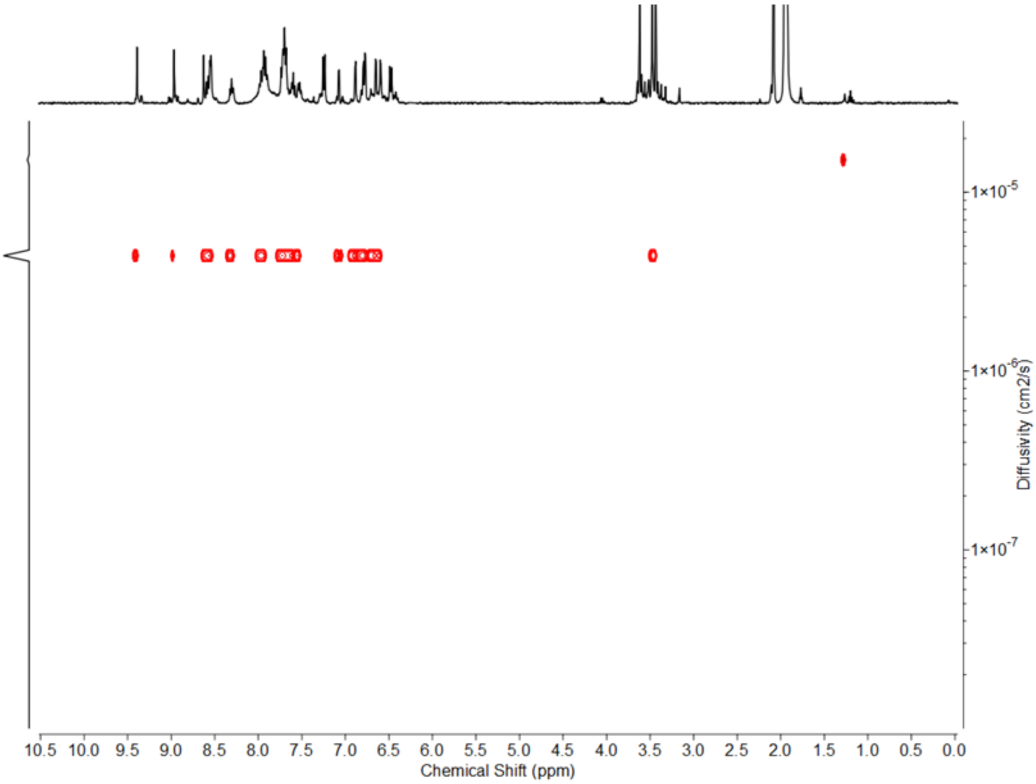

Figure S22. ^{1}H DOSY spectrum of cage **5** (400 MHz, CD_3CN, 25 °C). The diffusion coefficient was measured to be 4.19×10^{-6} cm^2/s.

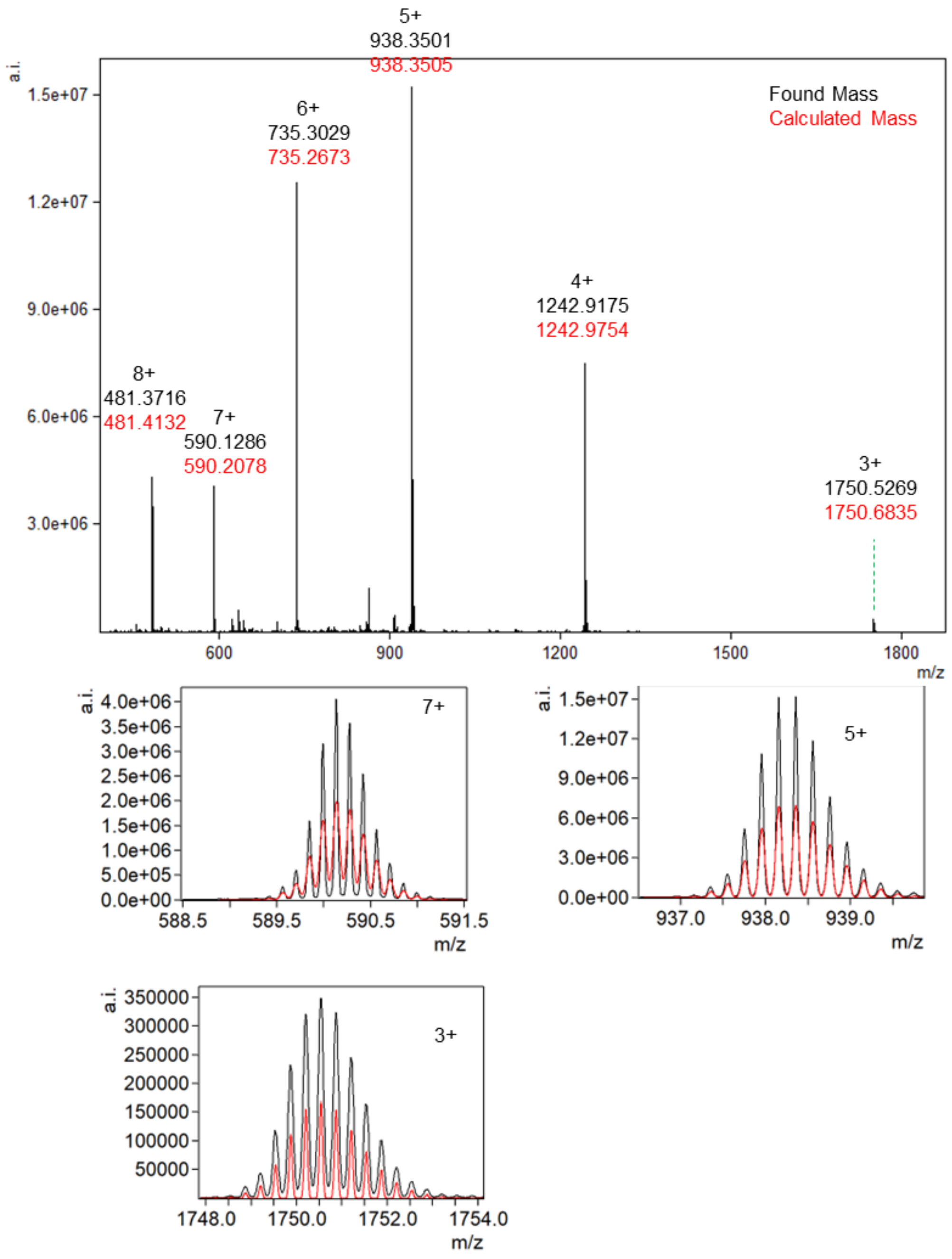

Figure S23. High-resolution ESI-MS spectrum of cage **5** in MeCN.

4 Conversion of Sandwich 2 to Cage 1

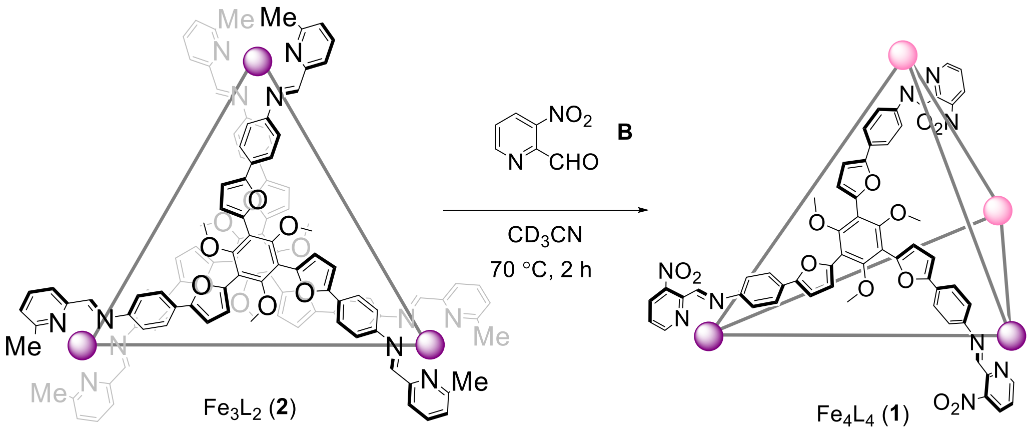

To a solution of sandwich **2** (5.6 mg, 1.5 µmol, 1.0 equiv) in CD_3CN (0.5 mL) was added excess 6-nitro-2-formylpyridine **B** in a J Young NMR tube. The reaction mixture was stirred at 70 °C under nitrogen. The solution colour changed from yellow to purple. After 2 hours, peaks corresponding to sandwich **2** were not observed in the ^1H NMR spectrum, while cage **1** was observed to be formed.

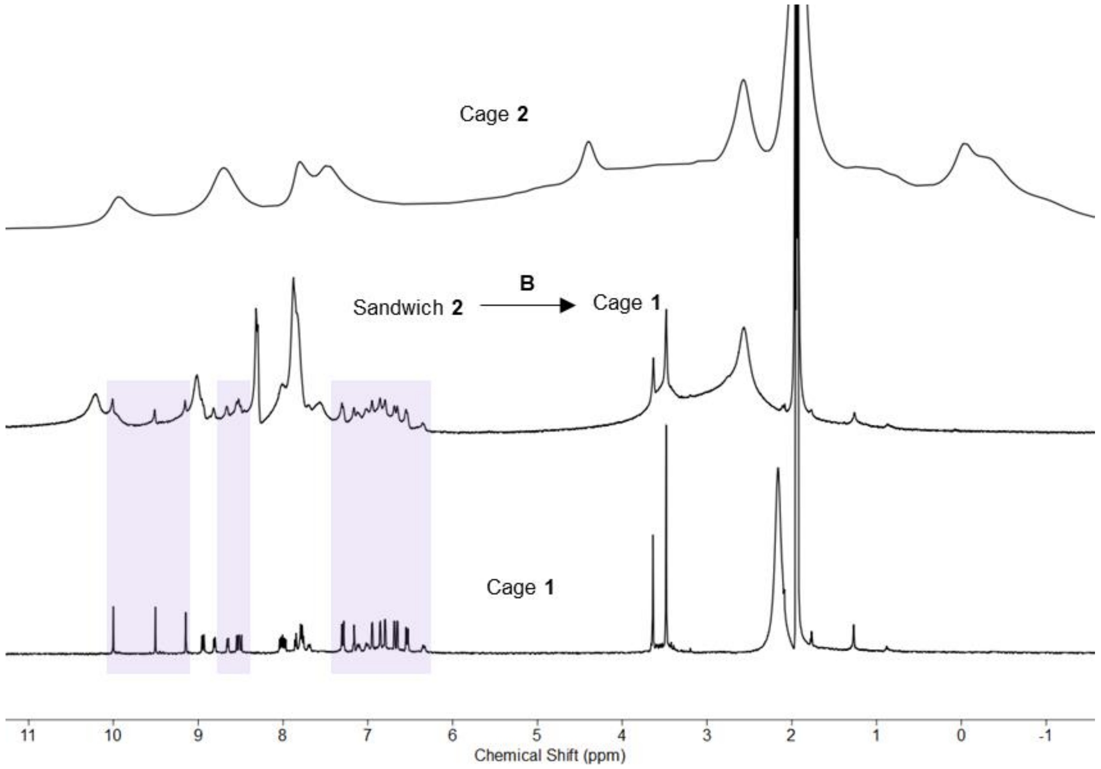

Figure S24. Comparison of ^1H NMR spectra of **1**, **2** and the conversion of **2** to **1** (400 MHz, CD_3CN, 25 °C).

5 Host-Guest Properties of 1

Host-guest complexes were prepared on an NMR scale in CD_3CN (0.5 mL). The NMR spectra were measured upon either shaking the host-guest mixtures for 1-5 mins (fast exchange) or heating the mixtures at 70 °C for 2 h (slow exchange). Binding affinities were quantified by ^{1}H NMR titrations, and binding stoichiometries were determined by either NMR or ESI-MS.

The van der Waals volumes of **G1**, **G3** and **G4** were calculated to be 321 Å^3, 596 Å^3 and 471 Å^3, respectively. Encapsulation of **G3** and **G4** by S_4-symmetric **1** gave rise to T-symmetric host-guest complex **G3**$\subset$**1** and C_3-symmetric host-guest complex **G4**$\subset$**1**, respectively. The cavity occupancies of **G3** and **G4** against the cavity volume of S_4-symmetric **1** (ca. 986 Å^3) are 60% and 48%, respectively. Cage **1** displayed remarkable structural flexibility and could adapt its configuration for favored guest. In accordance with Rebek's 55% rule,[4] we inferred that **G3** might occupy around 55% of the cavity of T-symmetric host framework, while **G4** might likewise occupy around 55% of the cavity of C_3-symmetric host framework.

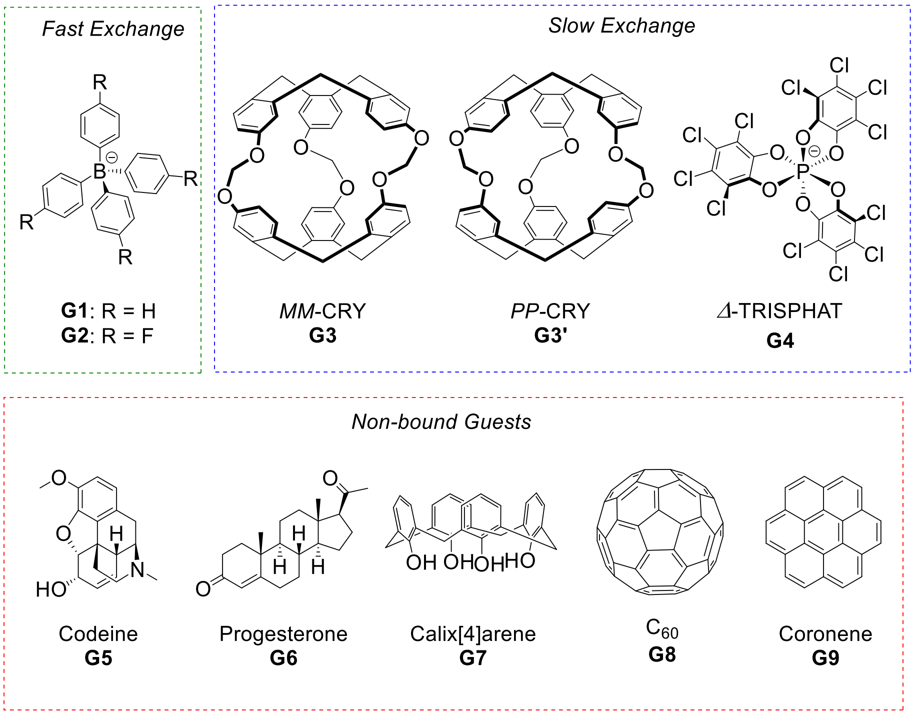

5.1 Host-Guest Interaction of 1 with G1 and G2

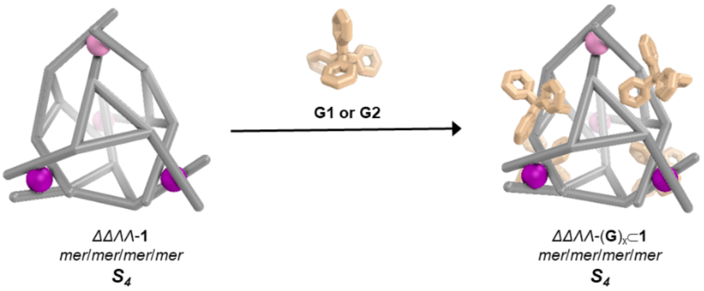

Cage **1** was prepared in 0.5 mL CD_3CN with a concentration of 0.50 mM; **G1** or **G2** were prepared in CD_3CN with a concentration of 50 mM. The guest solution was added into the host solution in an NMR tube. ^{1}H NMR data were collected after shaking the host-guest mixture for 1-5 minutes.

Titration data were fitted to 1:1 and 1:2 systems using BindFit;[5] the data fit both systems with acceptable residuals. The exact binding stoichiometry therefore could not be gauged; higher binding stoichiometry could also not be ruled out.

Apparent associate constant (K_a) and coefficient value (n) were determined by the Hill equation:

$$\theta = \frac{[\mathrm{HG}_n]}{[\mathrm{HG}_n]+[\mathrm{H}]} = \frac{[\mathrm{G}]^n}{[\mathrm{G}]^n+\left(\frac{1}{K_a}\right)^n}$$

and thus

$$\log(\theta/1-\theta) = n\log[\mathrm{G}] + n\log K_a$$

where θ is the fraction of host bound by the guest which is determined by observed chemical shifts (Δδ) against the maximum chemical shift during titrations ($\Delta\delta_{max}$), [G] is the guest concentration, n is the Hill coefficient describing cooperativity, and K_a is the apparent association constant.

Cooperativity is quantified by the Hill coefficient *n*, where $n > 1$ indicates positively cooperative binding, $n < 1$ indicates negatively cooperative binding, and $n = 1$ indicates noncooperative binding.

Chemical shift changes of **1** were plotted and fitted with a Hill function with apparent associate constants determined to be $(1.82 \pm 0.05) \times 10^2$ M^{-1} and $(1.21 \pm 0.05) \times 10^2$ M^{-1} for **G1** and **G2**, respectively. In both cases, Hill coefficients were determined to be approximate 1, indicating non-cooperative binding of tetraphenylborates by **1**. Proton signals of **1** were observed to shift upon addition of guests, particularly those of the furan and phenylene rings as well as OMe groups. Moreover, when **1** (ca. 986Å^3) centrally bound **G3** (596Å^3) and **G4** (471 Å^3) as shown in Section 5.4, the simultaneous binding of **G1** (321Å^3) likewise occurred. These observations indicated that **G1** and **G2** might be bound peripherally at apertures of **1**. It was also observed that peripheral binding of tetraphenylborates during the titration process did not influence the S_4 symmetry of overall host-guest complex, again with two *mer-Δ* and two *mer-Λ* metal vertices.

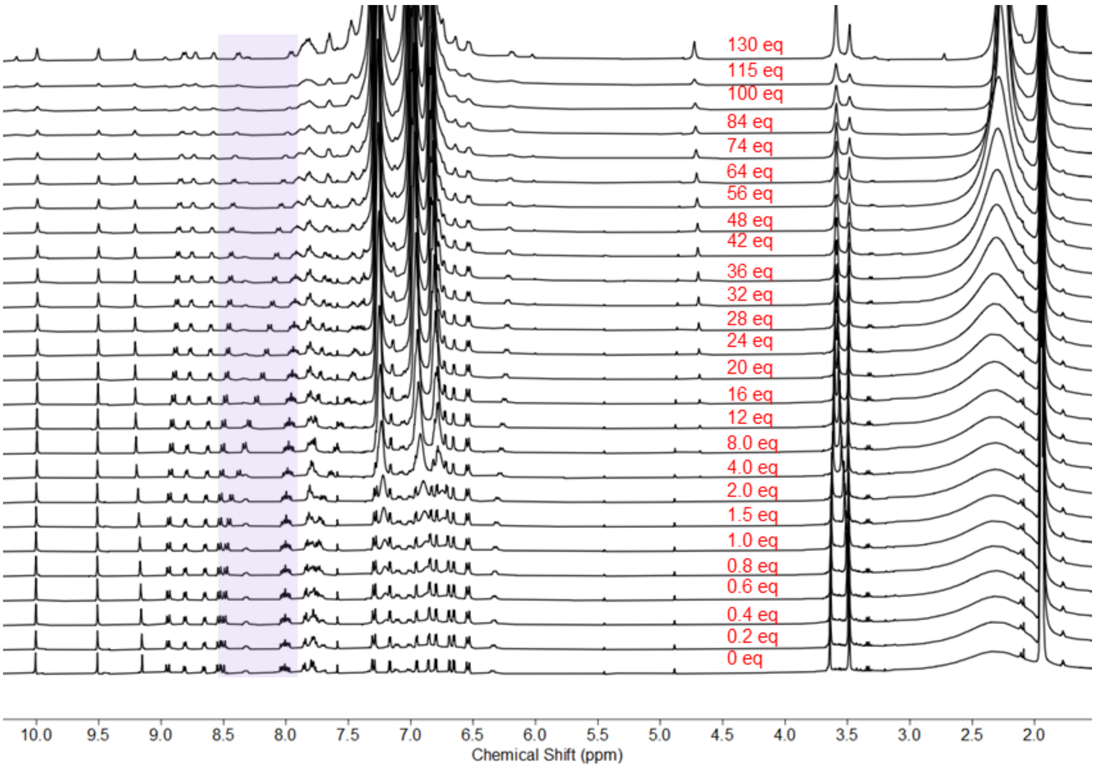

Figure S25. ^{1}H NMR spectra upon addition of **G1** into **1** (400 MHz, CD_3CN, 25 °C).

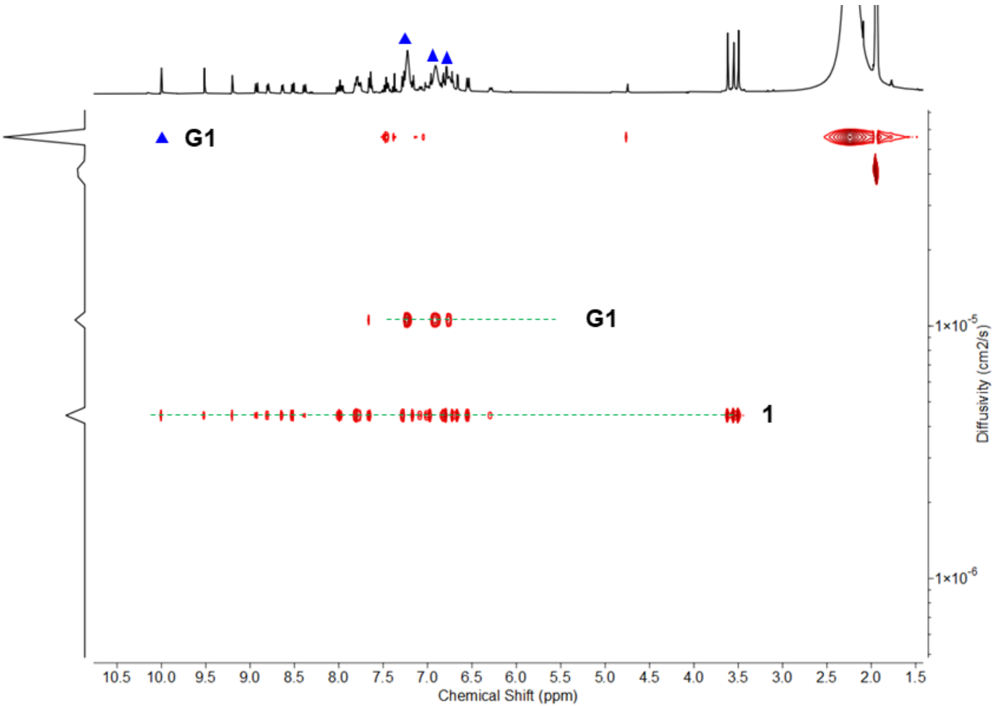

Figure S26. ^{1}H DOSY spectrum of **1** in presence of excess **G1** (400 MHz, CD_3CN, 25 °C). The diffusion coefficient was measured to be 4.38×10^{-6} cm^2/s.

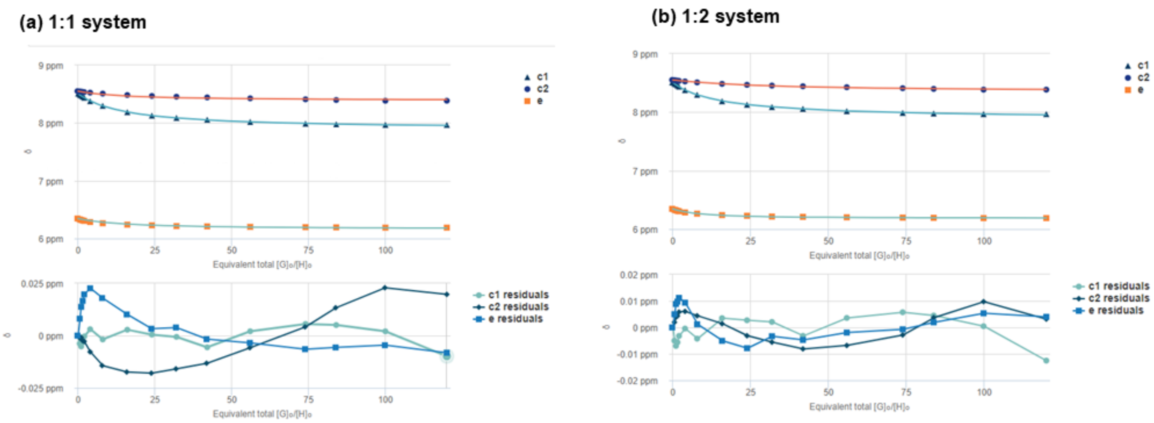

Figure S27. Binding isotherms and residual plots of binding **G1** by **1** using BindFit.[5] **(a)** 1:1 system, $K_a = 136 \pm 6$ M^{-1}. **(b)** 1:2 system, $K_{11} = 273 \pm 18$ M^{-1}, $K_{12} = 52 \pm 3$ M^{-1}.

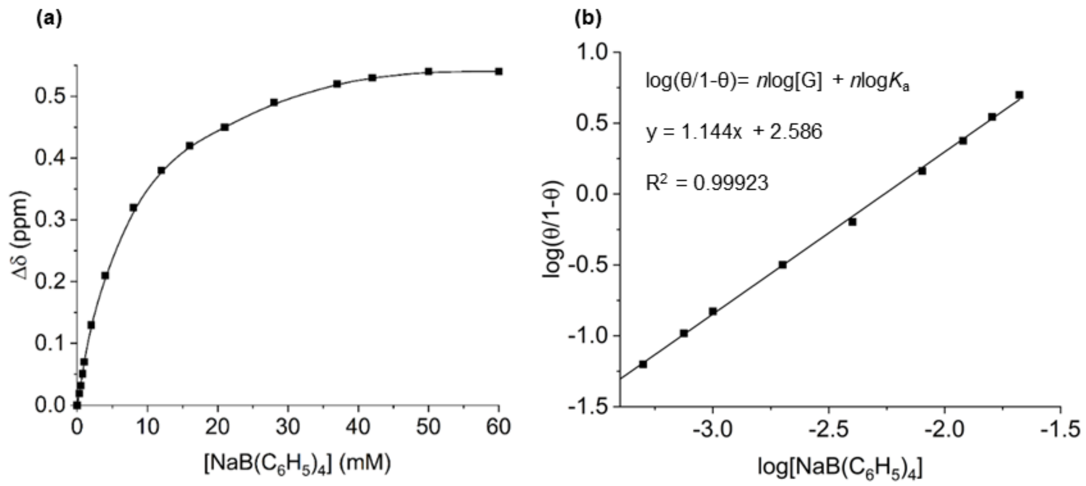

Figure S28. Titration Analysis. **(a)** Titration curves of **G1** bound by **1**. **(b)** Hill function.

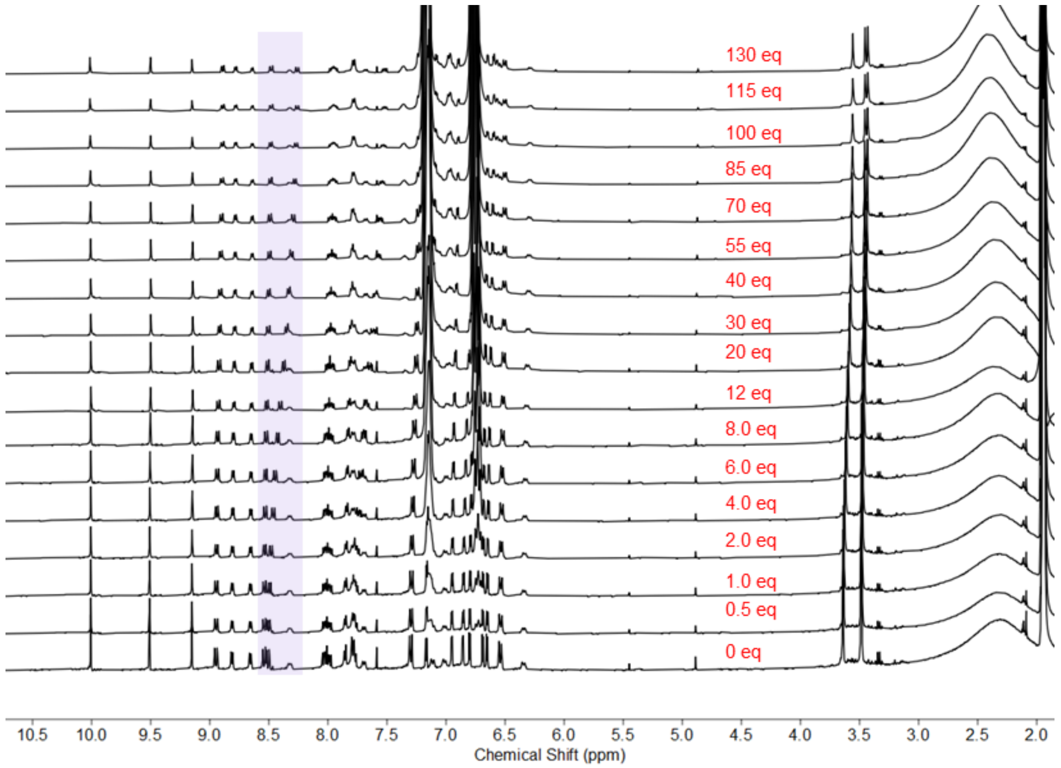

Figure S29. ^{1}H NMR spectra upon addition of **G2** into **1** (400 MHz, CD_3CN, 25 °C).

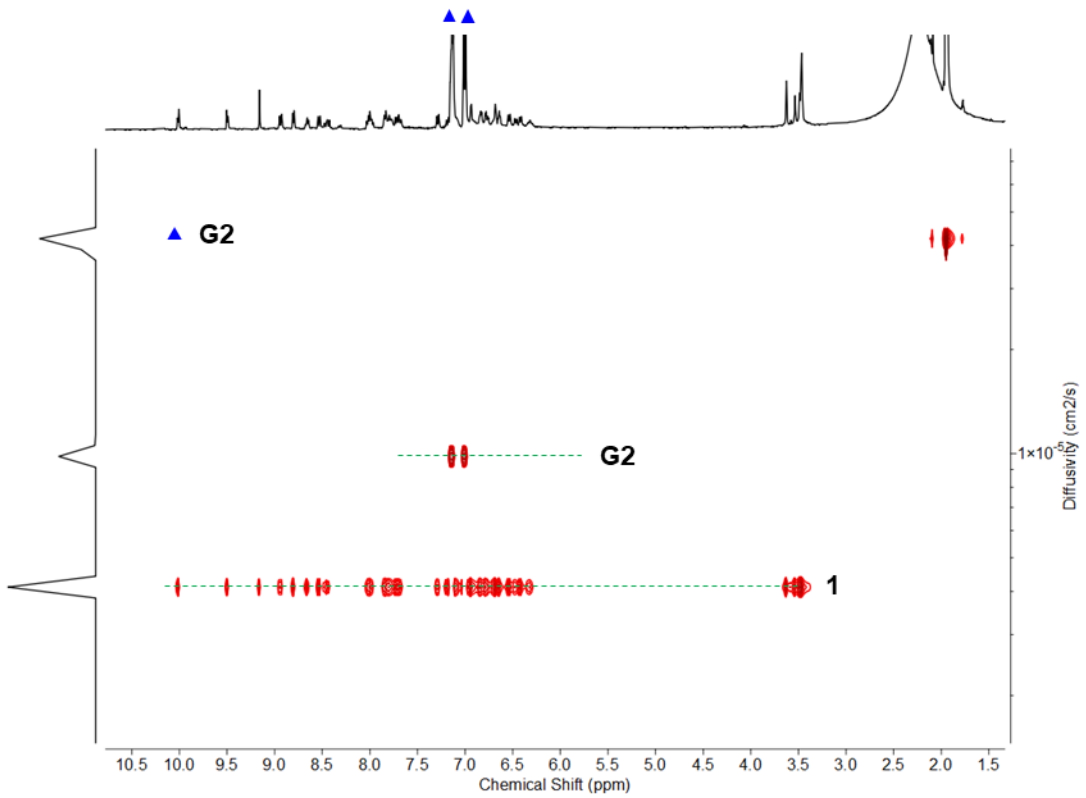

Figure S30. ^{1}H DOSY spectrum of **1** in presence of excess **G2** (400 MHz, CD_3CN, 25 °C). The diffusion coefficient was measured to be 4.18×10^{-6} cm^2/s.

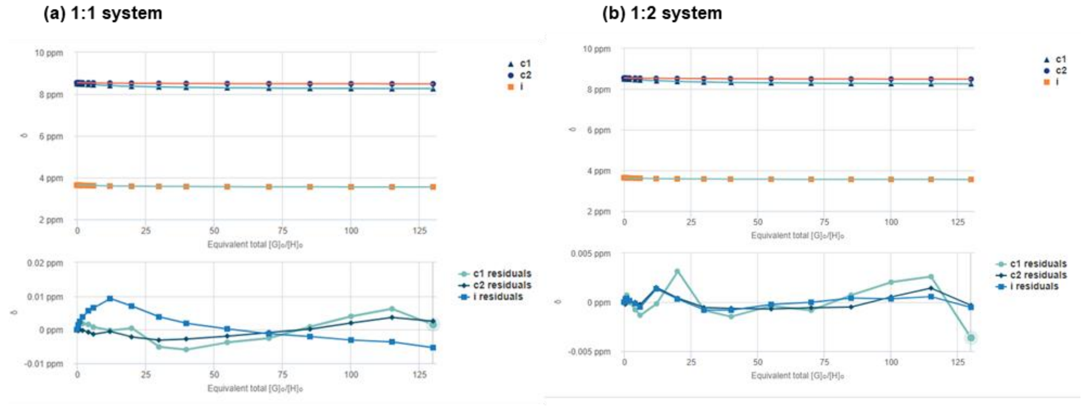

Figure S31. Binding isotherms and residual plots of binding **G2** by **1** using BindFit.[5] **(a)** 1:1 system, K_a = 88 ± 3 M^{-1}. **(b)** 1:2 system, K_{11} = 203 ± 6 M^{-1}, K_{12} = 28 ± 1 M^{-1}.

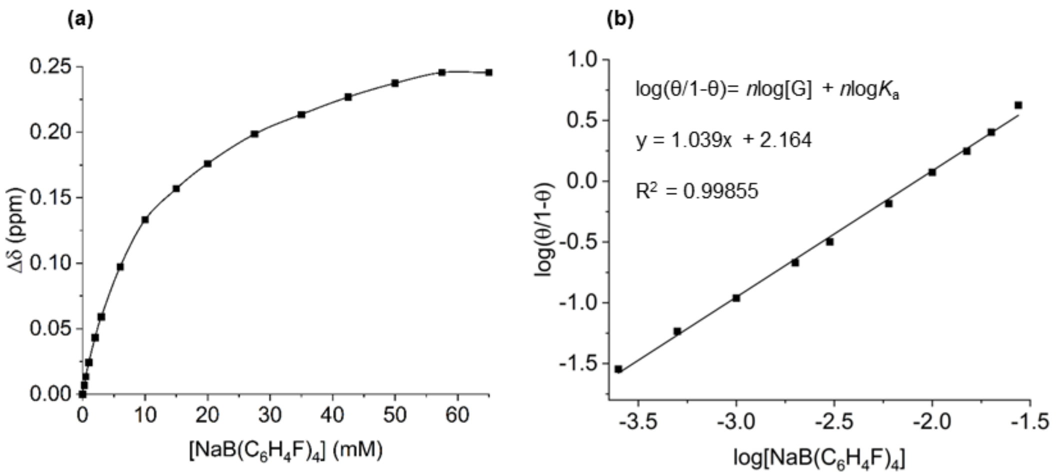

Figure S32. Titration Analysis. **(a)** Titration curves of **G2** bound by **1**. **(b)** Hill function.

5.2 Host-Guest Interaction of 1 with G3 and G3'

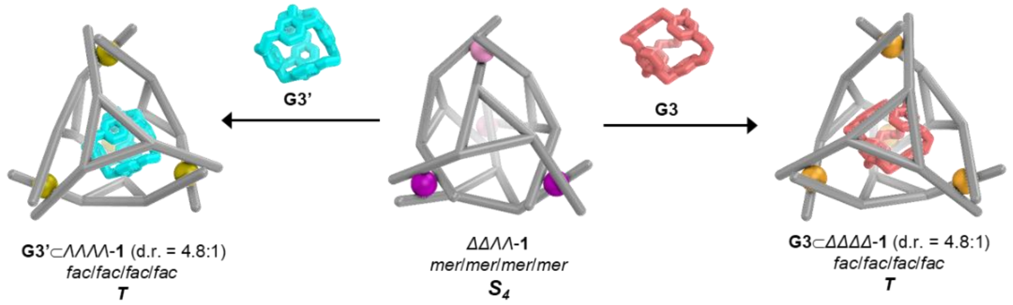

To a solution of cage **1** in 0.5 mL CD_3CN (0.75 mM) was added **G3** or **G3'** portionwise, using *tert*-butyl acetate as internal standard (0.75 mM). After each addition, the reaction mixture was heated at 70 °C for 2 h. ^1H NMR data was collected after cooling down the host-guest complex.

The binding constant was determined using the following equation:

$$K_a = \frac{[HG]}{[H][G]}$$

where [HG], [H] [G] is the concentration for the host-guest complex, host, and guest, respectively.

An average value of K_a from each addition of guest was calculated to quantify the binding affinity, with $K_a = (2.33 \pm 0.10) \times 10^3$ M^{-1} for binding **G3** and $K_a = (2.40 \pm 0.10) \times 10^3$ M^{-1} for binding **G3'**.

The ^{1}H NMR spectrum of **G3⊂1** was consistent with the formation of a *T*-symmetric species, with its Subcomponents in an environment having 3-fold symmetry and all metal centers adopting *facial* (*fac*) configurations with the same handedness. The presence of *fac* stereochemistry was also confirmed by the absence of NOE correlations between pyridyl and phenylene rings. During titration experiments, proton peaks corresponding to host-guest complex emerged at the expense of free **1** in the ^{1}H NMR spectrum, wherein proton integrations revealed the formation of a 1:1 host-guest complex **G3⊂1**, and all proton signals of bound guest significantly shifting up-field as a result of shielding effect indicated central binding of **G3**.

Two groups of proton peaks in a ratio of 4.8:1 observed in the ^{1}H NMR spectrum indicated that **G3⊂1** consists of a pair of diastereomers, that is, *MM*-CRY⊂*ΔΔΔΔ*-**1** and *MM*-CRY⊂*ΛΛΛΛ*-**1**. The circular dichroism (CD) spectrum of **G3⊂1** displayed strong Cotton effects around 280–400 nm and 410–680 nm (Figure S40), arising from π-π* and metal-to-Subcomponent charge transfer (MLCT) transitions, respectively. MLCT bands were correlated with the *Δ* handedness,[6] thus suggesting that the major diastereomer of *MM*-CRY⊂**1** adopts four *fac*-*Δ* metal vertices. Moreover, cage **1** was also capable of binding the mirror *PP*-CRY to yield *PP*-CRY⊂**1** possessing four *fac*-*Λ* metal centers as the major diastereomer. The characterization data of *MM*-CRY⊂**1** and *PP*-CRY⊂**1** is listed below.

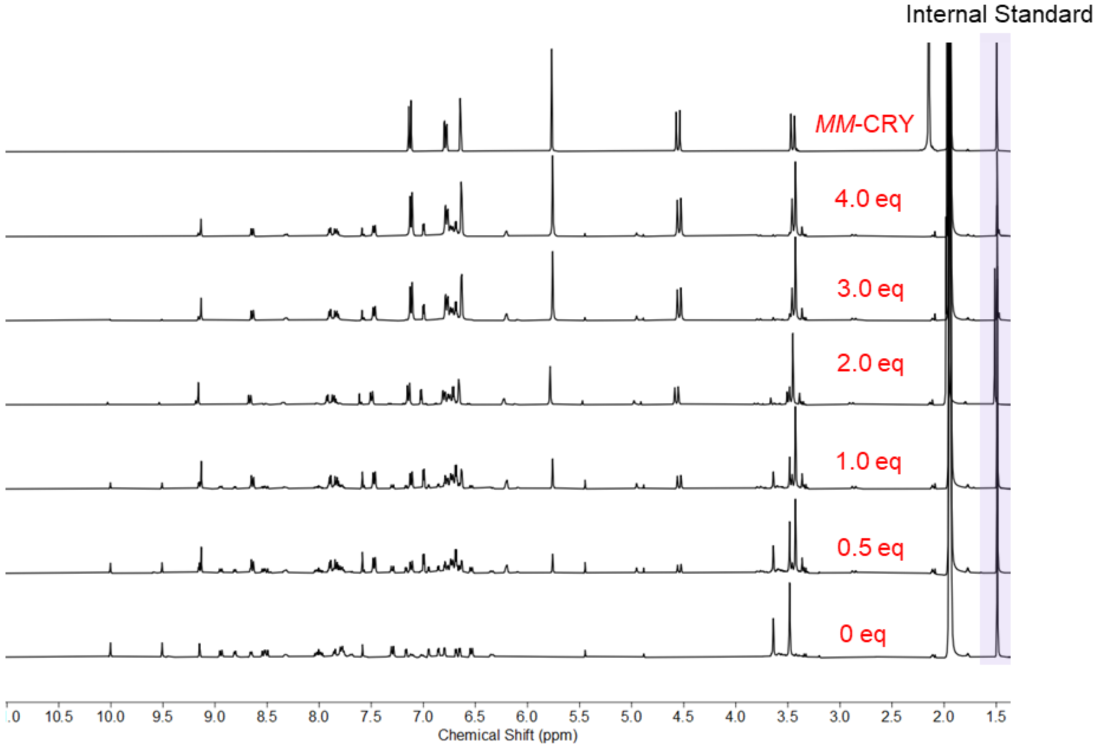

Figure S33. ^{1}H NMR spectra upon addition of **G3** into **1** (400 MHz, CD_3CN, 25 °C).

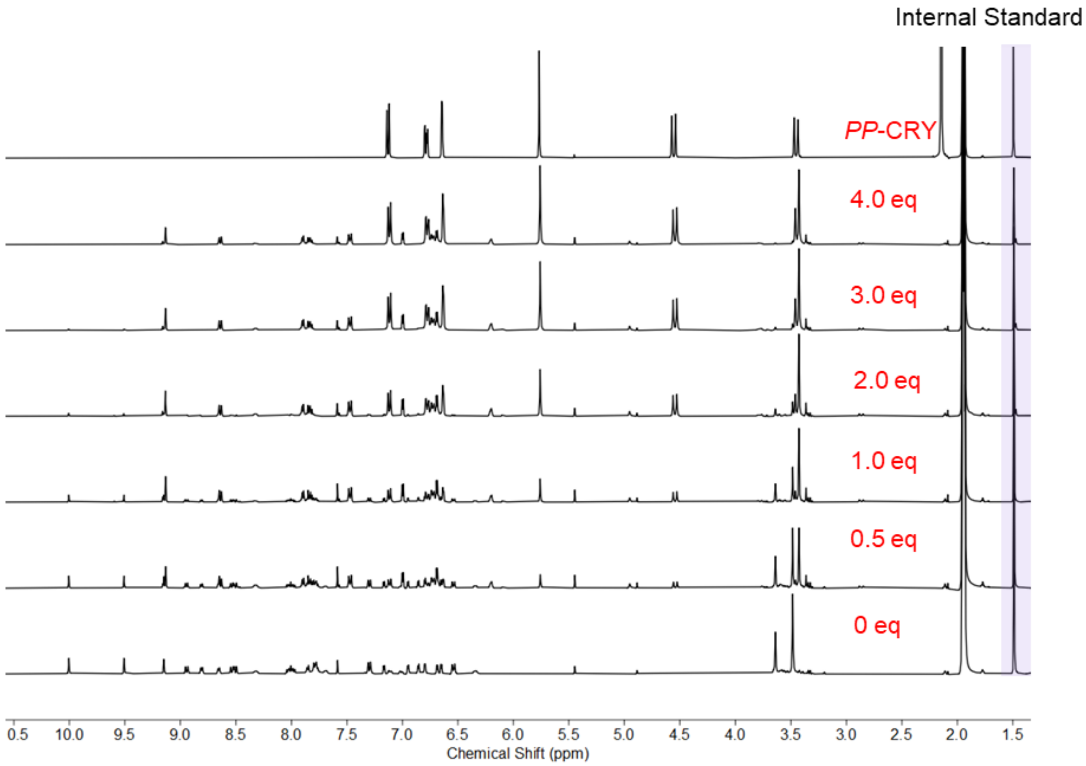

Figure S34. ^{1}H NMR spectra upon addition of **G3'** into **1** (400 MHz, CD_3CN, 25 °C).

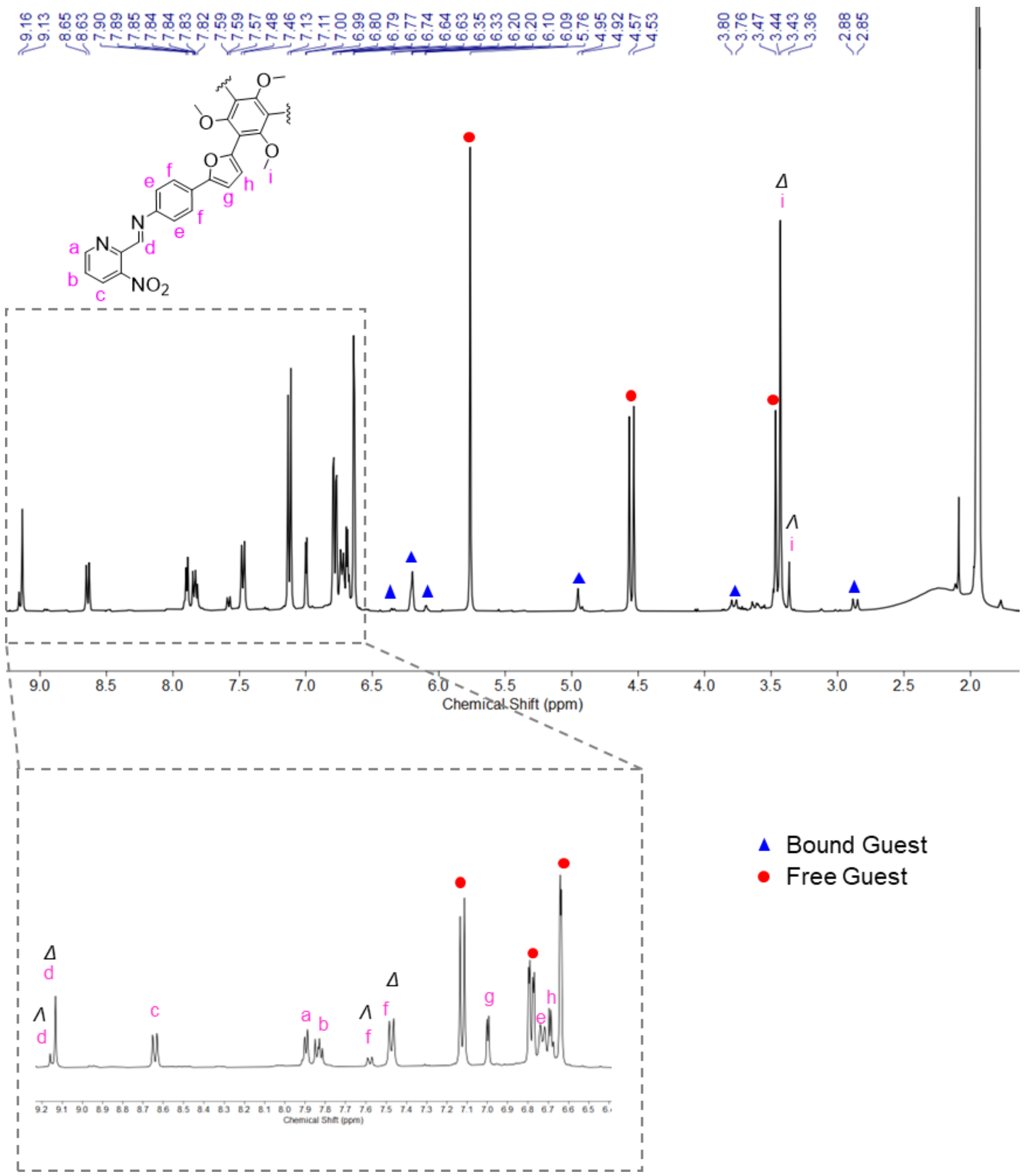

Figure S35. ^{1}H NMR spectrum of **G3⊂1** in presence of excess **G3** (400 MHz, CD_3CN, 25 °C).

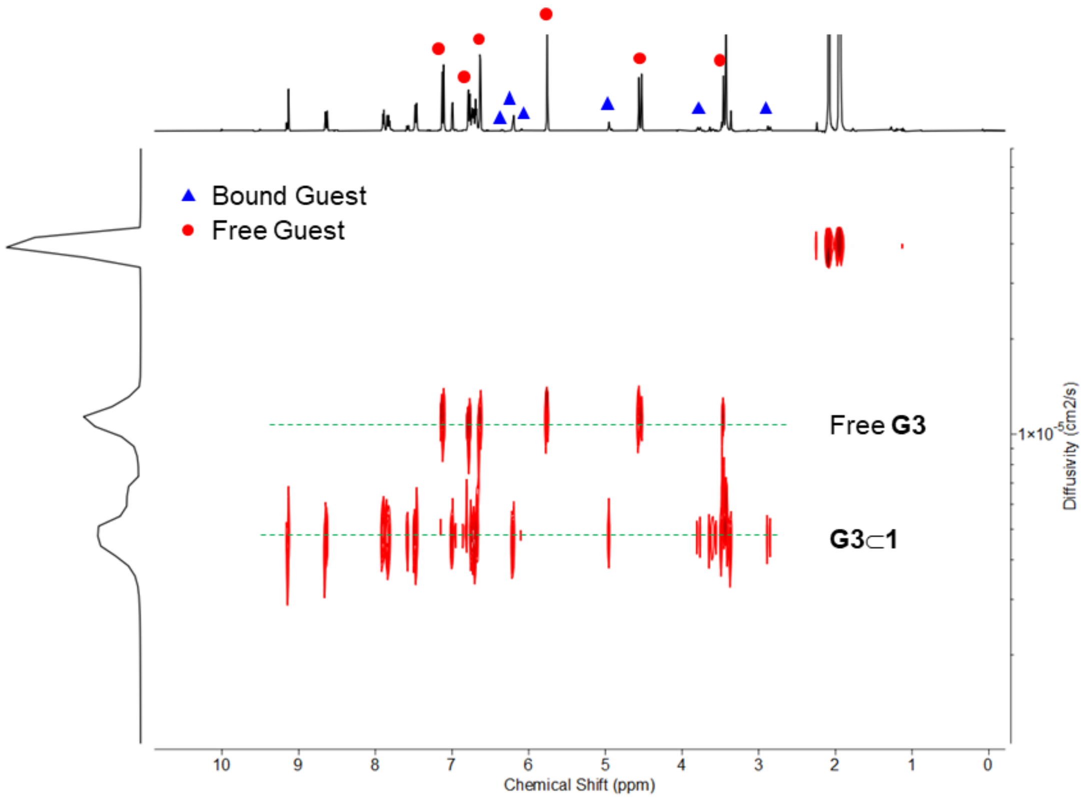

Figure S36. ^{1}H DOSY spectrum of **G3⊂1** in presence of excess **G3** (400 MHz, CD_3CN, 25 °C). The diffusion coefficient was measured to be 4.71×10^{-6} cm^2/s.

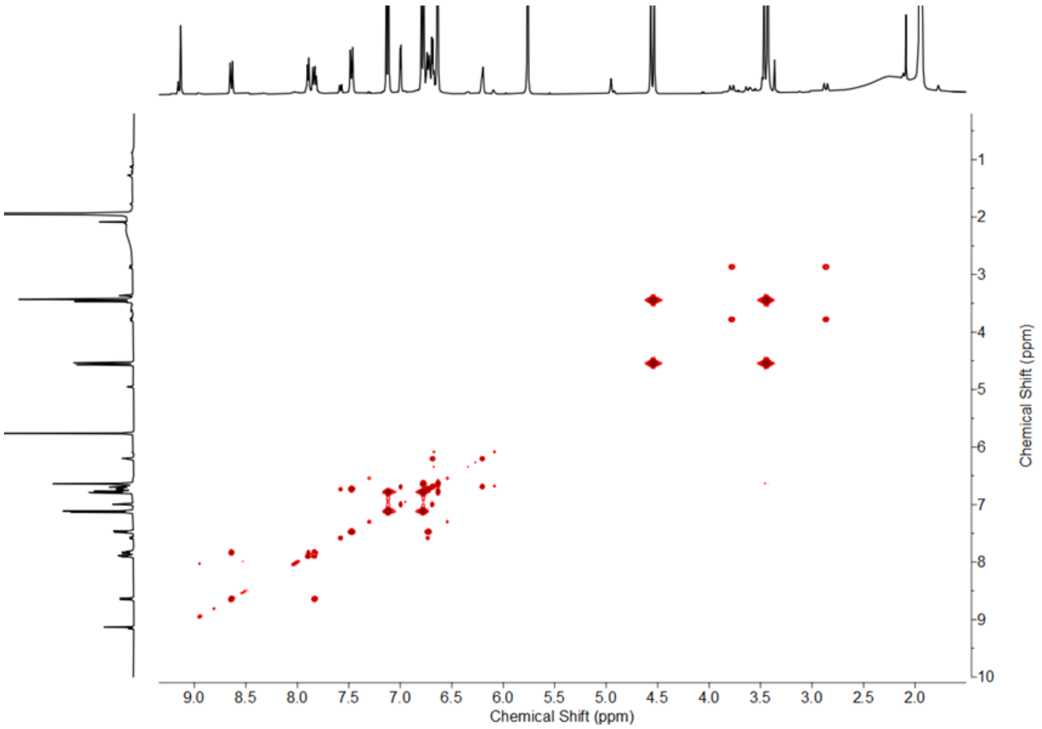

Figure 37. ^{1}H-^{1}H COSY NMR spectrum of **G3⊂1** in presence of excess **G3** (500 MHz, CD_3CN, 25 °C).

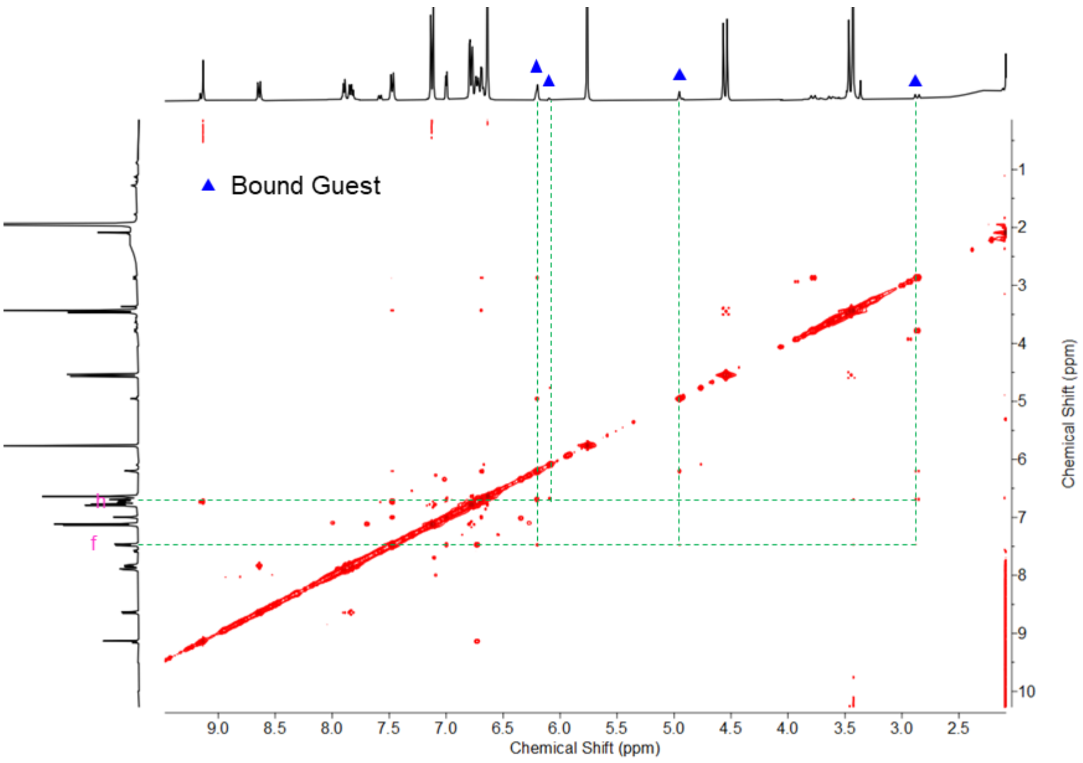

Figure 38. ^{1}H-^{1}H NOESY NMR spectrum of **G3⊂1** in presence of excess **G3** (500 MHz, CD_3CN, 25 °C).

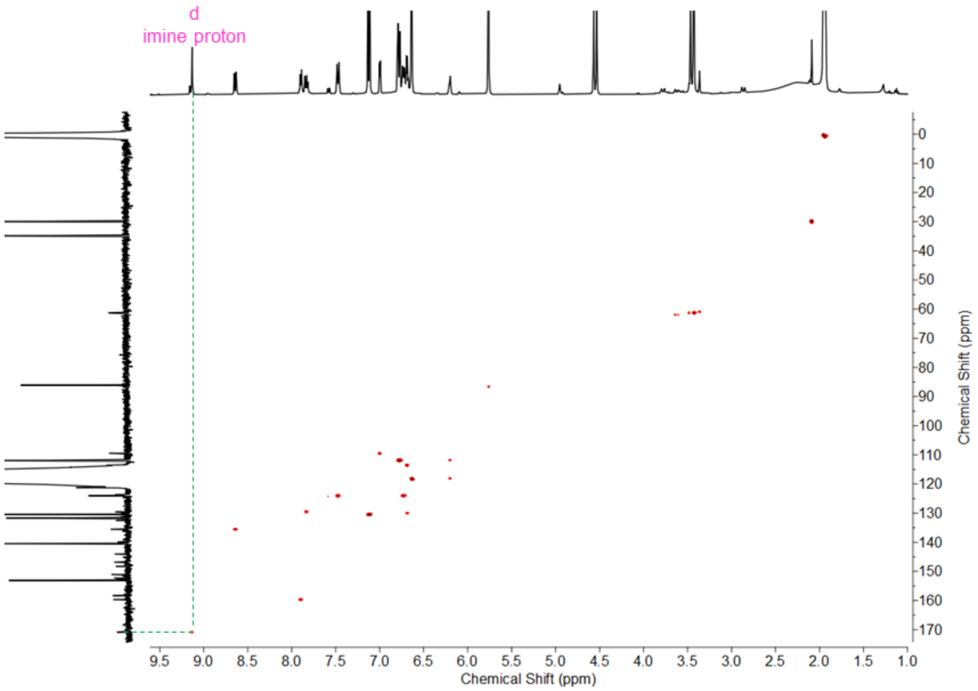

Figure 39. ^{1}H-^{13}C HSQC NMR spectrum of **G3⊂1** in presence of excess **G3** (500 MHz, CD_3CN, 25 °C).

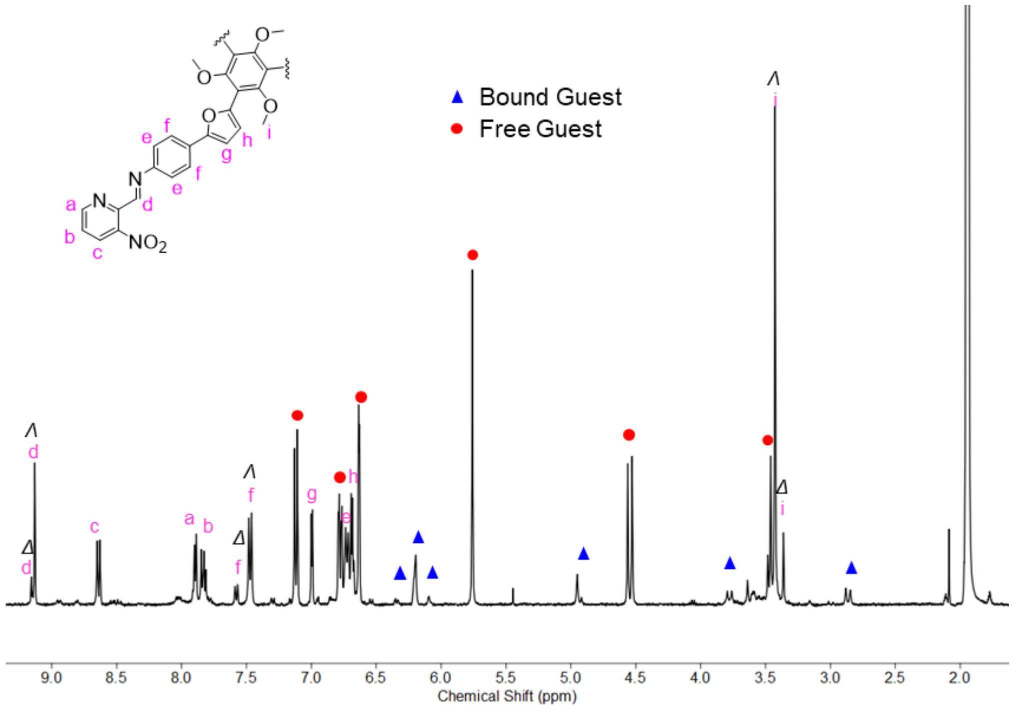

Figure S40. ^{1}H NMR spectrum of **G3'⊂1** in presence of excess **G3'** (400 MHz, CD_3CN, 25 °C).

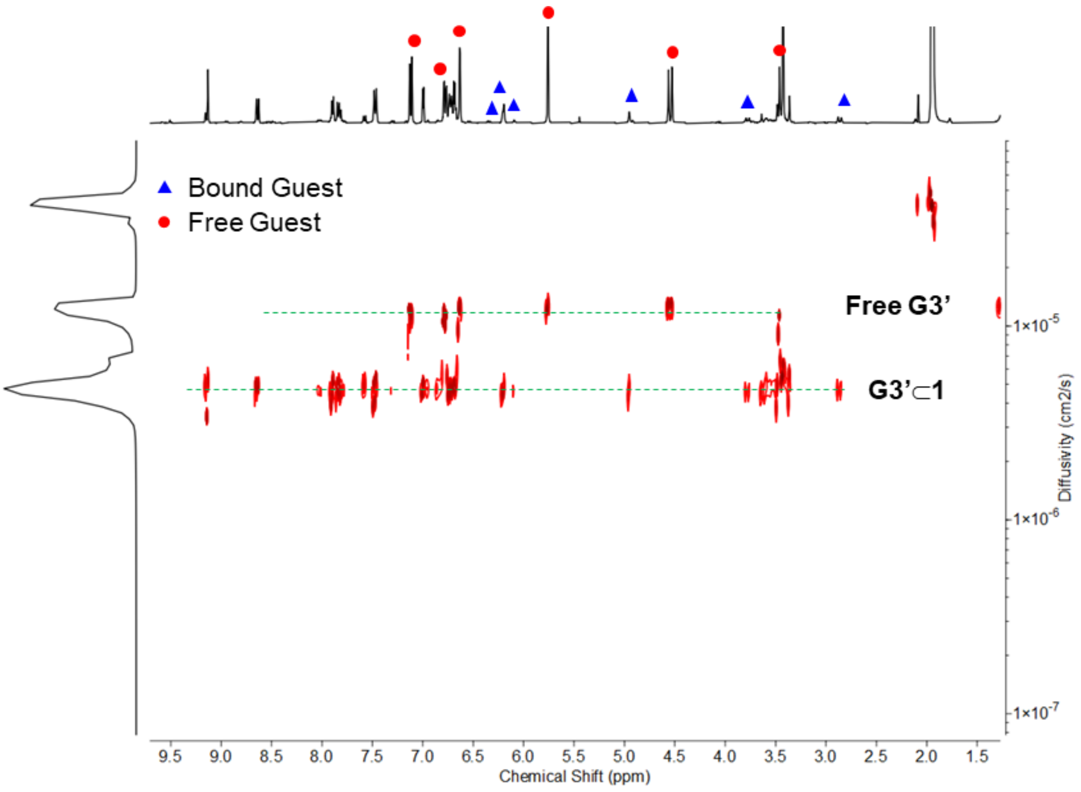

Figure 41. ^{1}H DOSY spectrum of **G3'⊂1** in presence of excess **G3'** (400 MHz, CD_3CN, 25 °C). The diffusion coefficient was measured to be 4.75×10^{-6} cm^2/s.

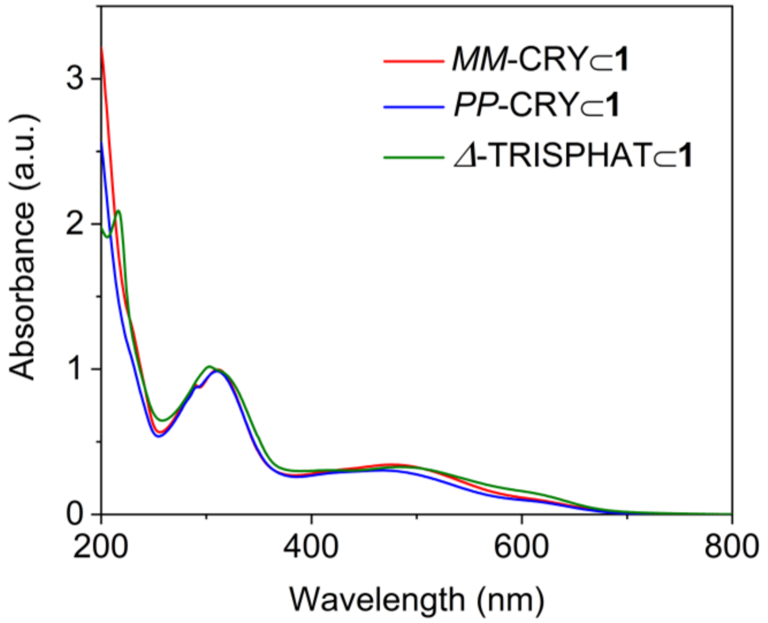

Figure S42. UV-vis spectra of host-guest complexes in MeCN.

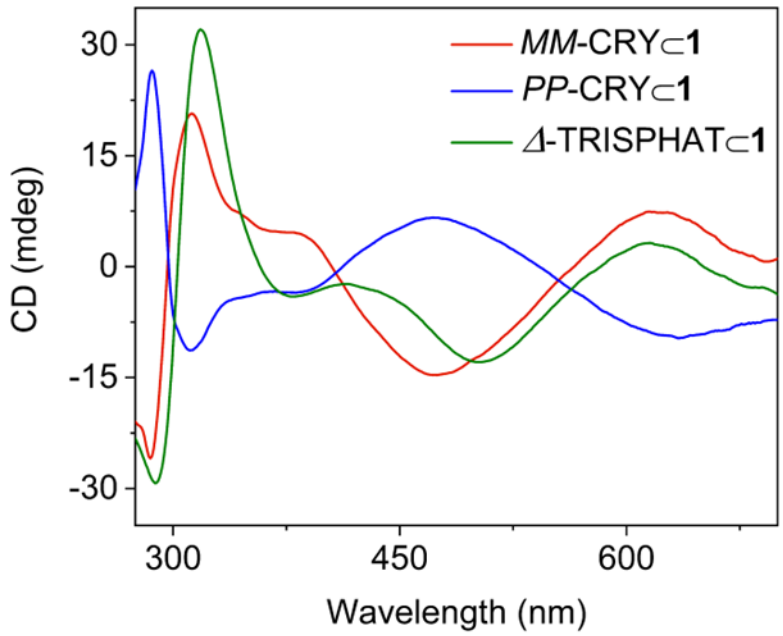

Figure S43. CD spectra of host-guest complexes in MeCN.

5.3 Host-Guest Interaction of 1 with G4

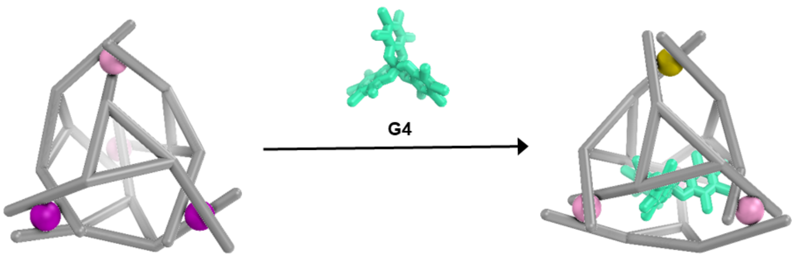

$\Delta\Delta\Lambda\Lambda$-1 *mer/mer/mer/mer* S_4 **G4⊂$\Delta\Delta\Delta\Lambda$-1** *mer/mer/mer/fac* C_3

To a solution of cage **1** in 0.5 mL CD_3CN (0.75 mM) was added **G4** portionwise, using *tert*-butyl acetate as internal standard (0.75 mM). After each addition, the reaction mixture was heated at 70 °C for 2 h. ^{1}H NMR data was collected after cooling down the host-guest complex.

The binding constant was determined using the following equation:

$$K_a = \frac{[HG]}{[H]([G0]-[HG])}$$

where [HG], [H] are the host-guest complex and host concentration, respectively, and [G0] is the concentration of the guest after it was added into the host solution.

An average value of K_a from each addition of guest was calculated to quantify the binding affinity, with a binding constant of $K_a = (1.17 \pm 0.15) \times 10^3$ M^{-1} for binding **G4**.

When extra *Δ*-TRISPHAT more than one equivalent was added during titrations, no changes of peaks corresponding to host-guest complex in ^{1}H and ^{31}P NMR spectra were observed, suggesting the formation of a 1:1 host-guest complex **G4⊂1** with its composition further confirmed by ESI-MS. The ^{1}H NMR spectrum showed two groups of signals with each group having four sets of peaks, consistent with **G4⊂1** existing as two C_3-symmetric diastereomers in a 4.1:1 ratio. As for the major diastereomer, NOE correlations provided evidence for a 1:3 *fac*:*mer* configuration of metal centers, while the MLCT bands in CD spectrum suggested that there is an excess of *Δ* stereochemical configurations within **G4⊂1** (Figure S43).[6] We thus inferred that the major diastereomer of **G4⊂1** with C_3 symmetry consists of one *fac-Λ* and three *mer-Δ*

metal centers, in analogy to a structure type first reported by Hooley and co-workers.[7] The characterization data for *Δ*-TRISPHAT⊂**1** are provided below.

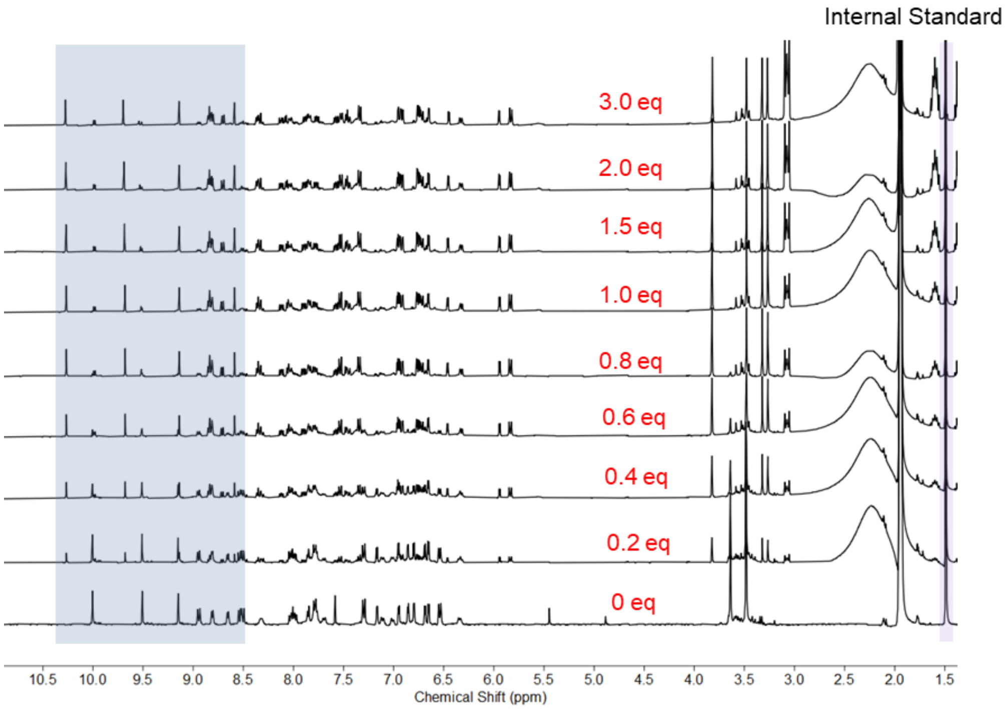

Figure S44. ^{1}H NMR spectra upon addition of **G4** into **1** (400 MHz, CD_3CN, 25 °C).

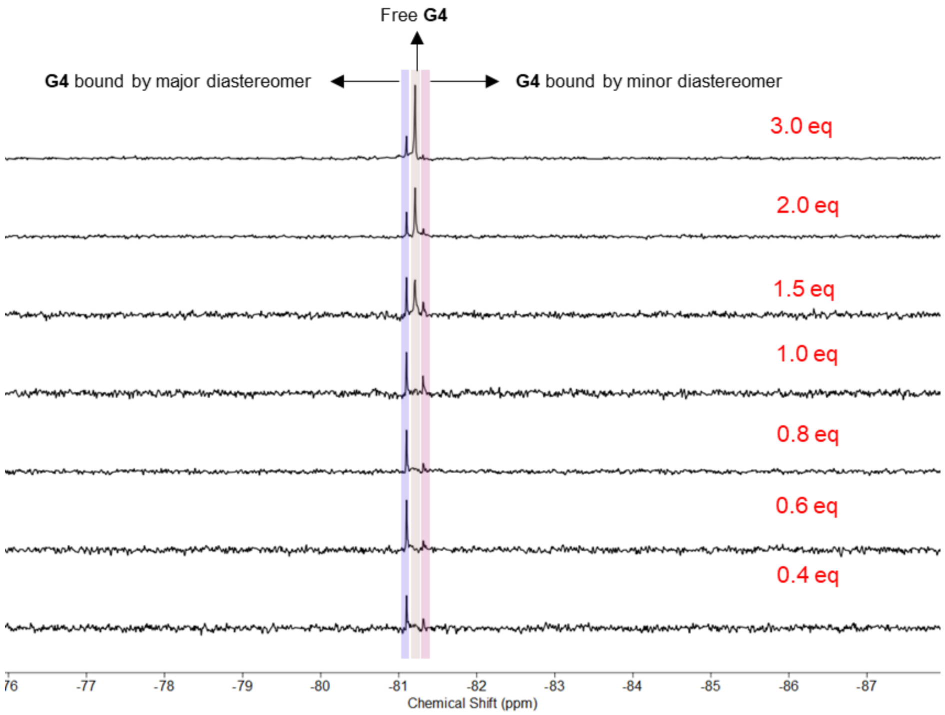

Figure S45. ^{31}P NMR spectra upon addition of **G4** into **1** (162 MHz, CD_3CN, 25 °C).

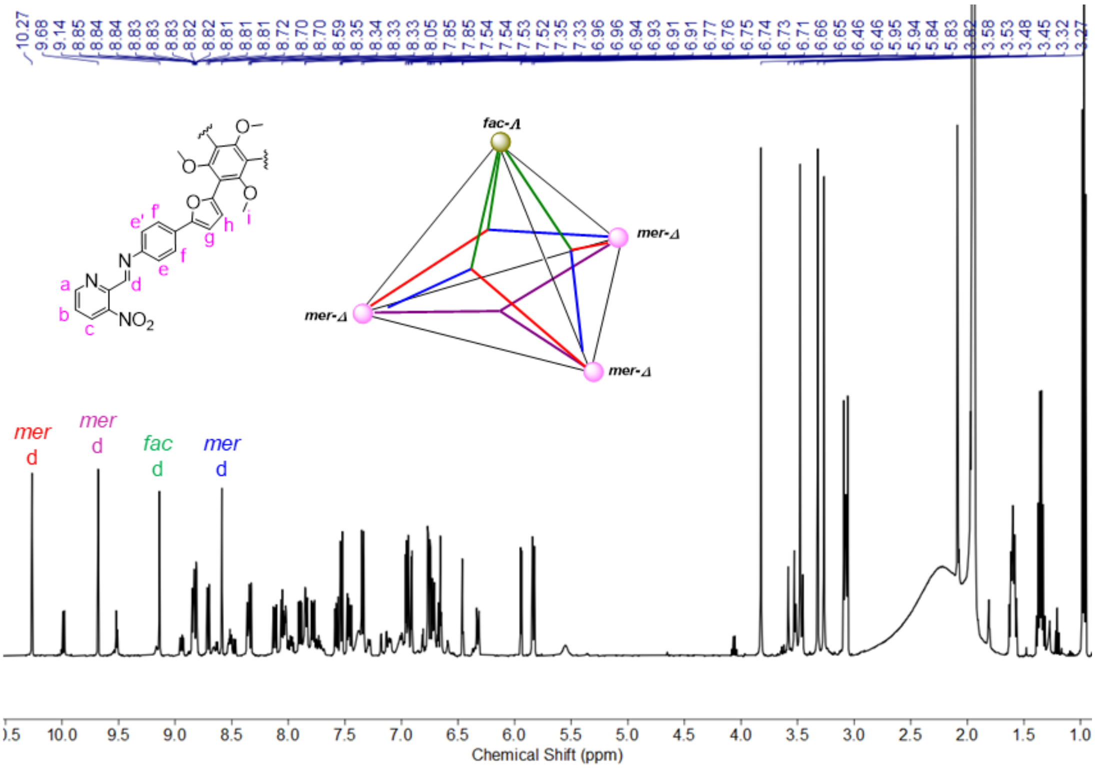

Figure S46. ^{1}H NMR spectrum of **G4⊂1** (500 MHz, CD_3CN, 25 °C).

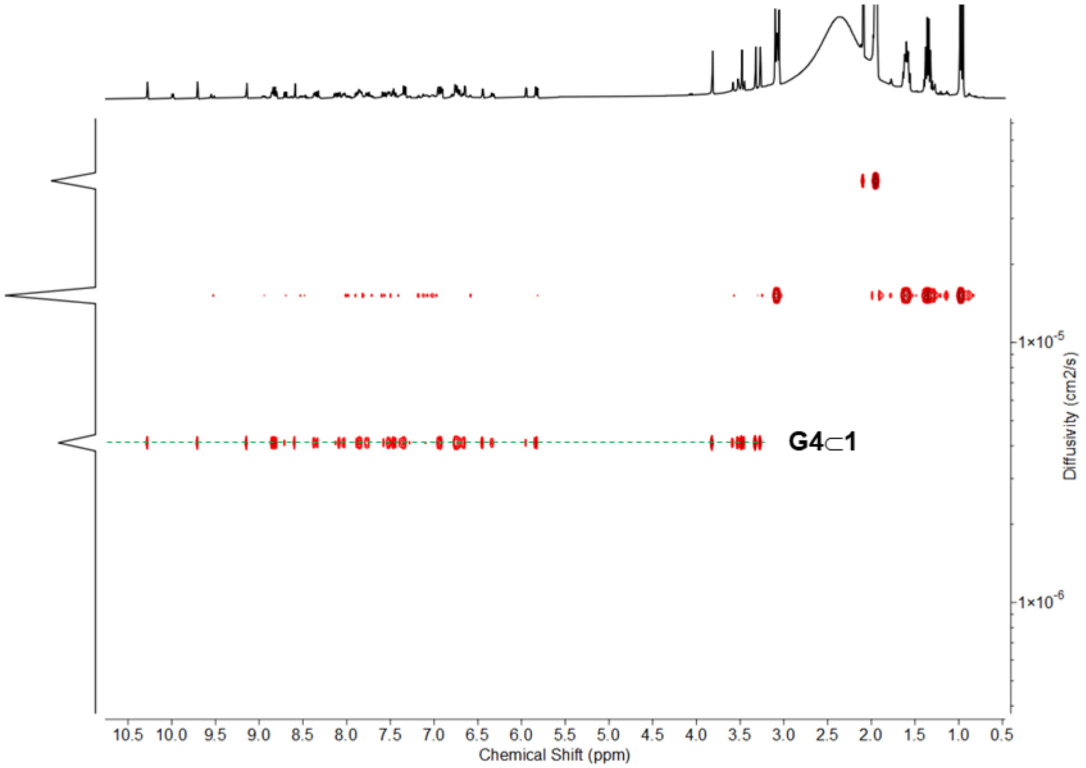

Figure S47. ^{1}H DOSY spectrum of **G4⊂1** (500 MHz, CD_3CN, 25 °C). The diffusion coefficient was measured to be 4.11×10^{-6} cm^2/s.

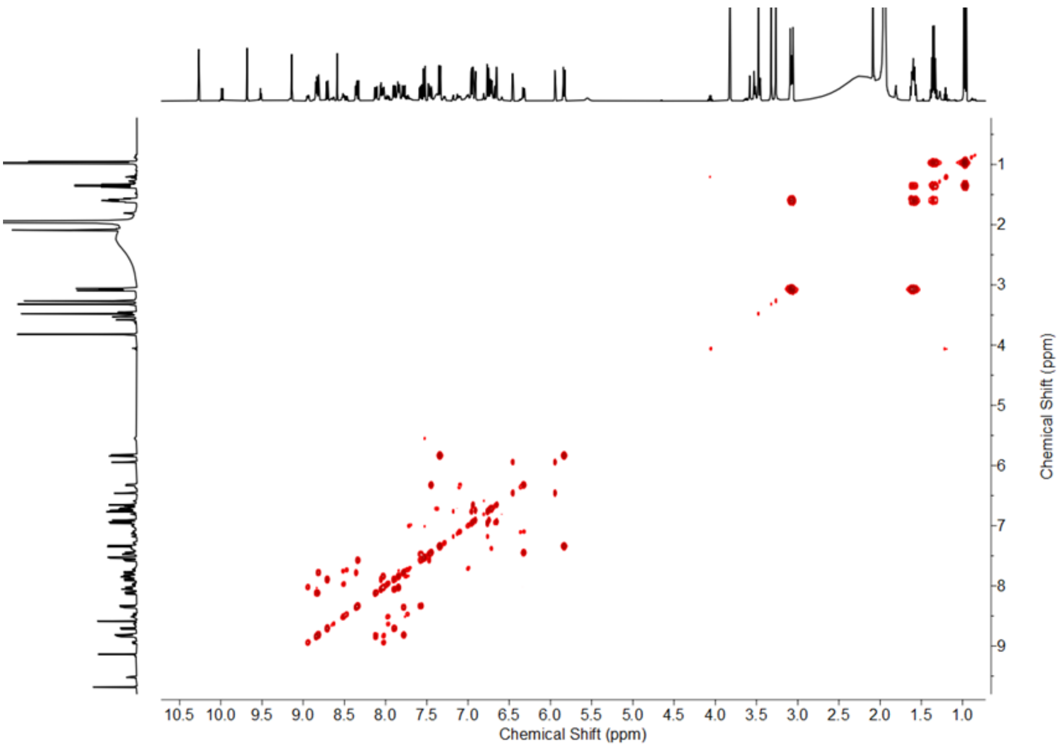

Figure 48. ^{1}H-^{1}H COSY NMR spectrum of **G4⊂1** (500 MHz, CD_3CN, 25 °C).

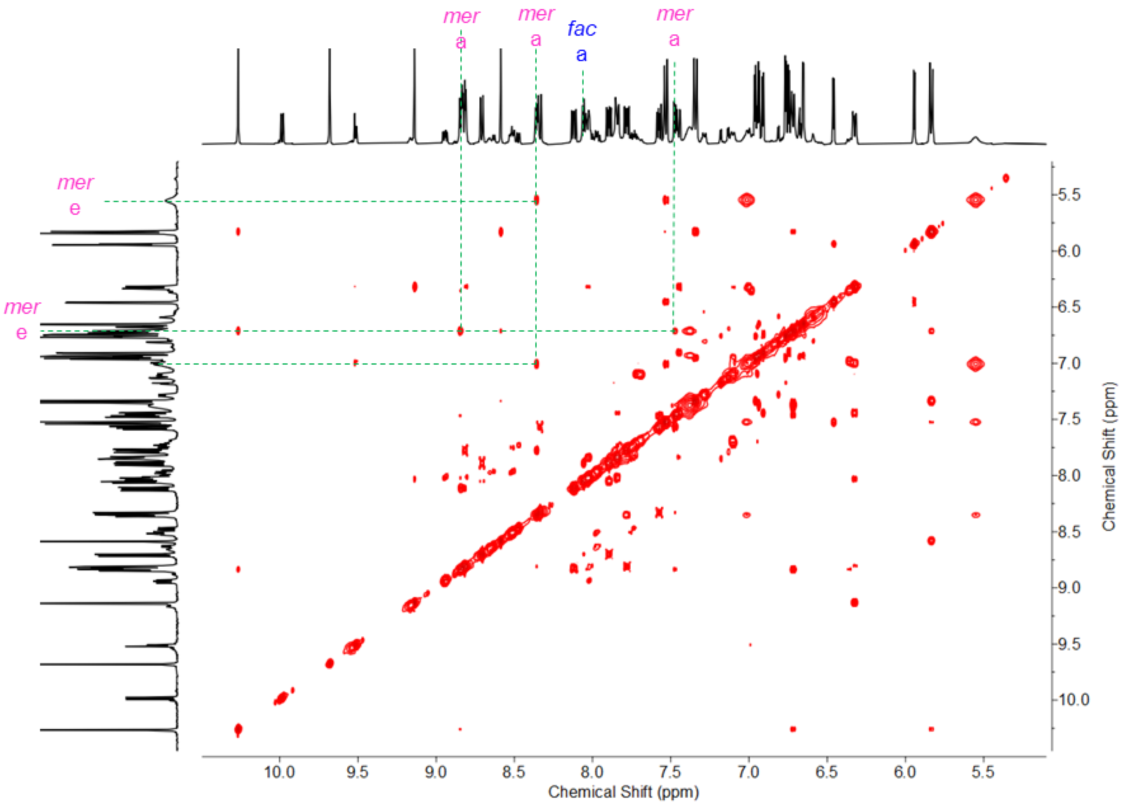

Figure 49. ^{1}H-^{1}H NOESY NMR spectrum of **G4⊂1** (500 MHz, CD_3CN, 25 °C).

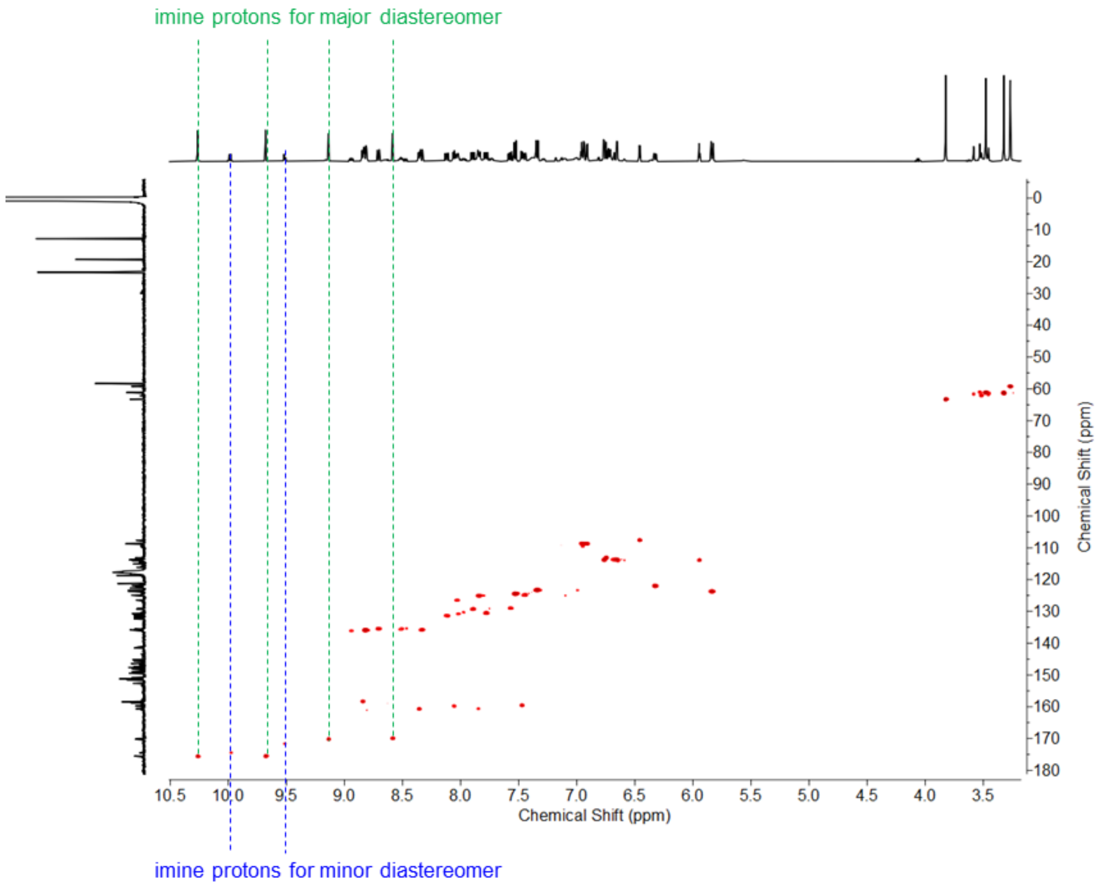

Figure 50. ^{1}H-^{13}C HSQC NMR spectrum of **G4⊂1** (500 MHz, CD_3CN, 25 °C).

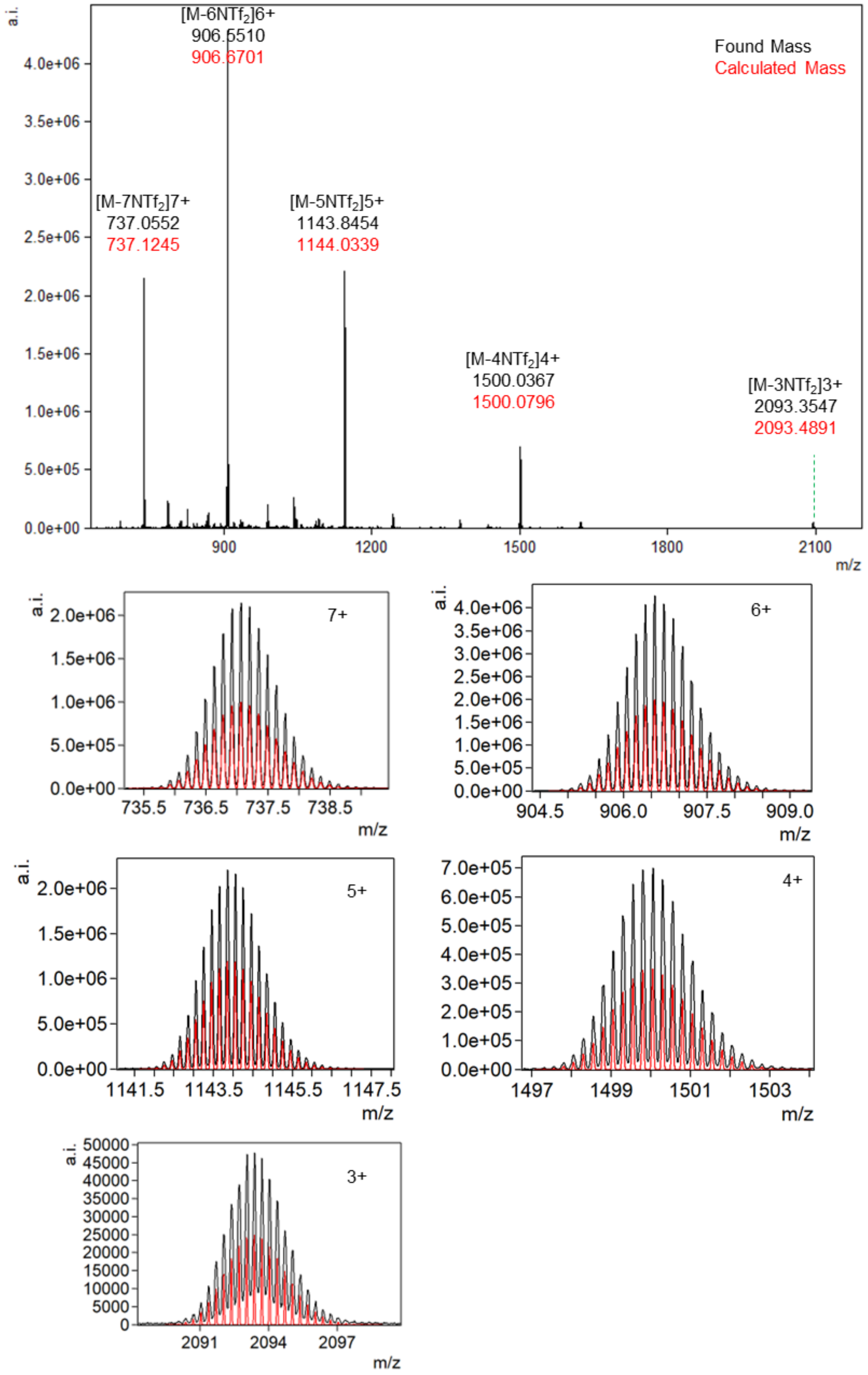

Figure S51. High-resolution ESI-MS spectrum of **G4**⊂**1** in MeCN.

5.4 Host-Guest Interaction of G3⊂1 and G4⊂1 with G1

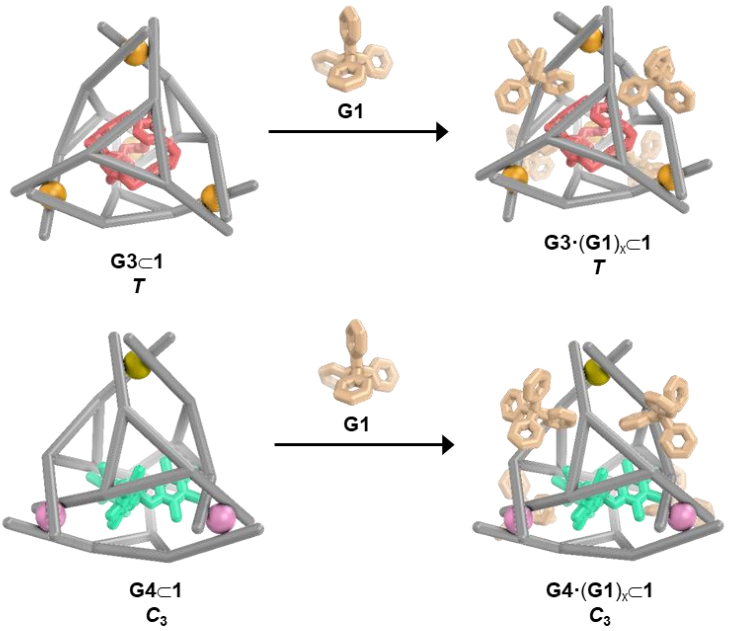

Titrations of G1 bound by G3⊂1: Host-guest complex **G3⊂1** was prepared in CD_3CN (0.50 mM) in presence of excess **G3** (4.0 equiv); **G1** was prepared in CD_3CN with a concentration of 50 mM. The guest solution was added into the host solution in an NMR tube. ^{1}H NMR data was collected after shaking the host-guest mixture for 1-5 minutes.

Titrations of G1 bound by G4⊂1: Host-guest complex **G4⊂1** was prepared in CD_3CN (0.50 mM) in presence of **G4** (1.05 equiv); **G1** was prepared in CD_3CN with a concentration of 50 mM. The guest solution was added into the host solution in an NMR tube. ^{1}H NMR data was collected after shaking the host-guest mixture for 1-5 minutes.

Binding constants were calculated by Hill equation, as described in section 5.1. In both cases, Hill coefficients were determined to be approximate 1, while apparent associate constants determined to be $(1.07 \pm 0.05) \times 10^2$ M^{-1} for **G1** bound by **G3⊂1** and $(1.35 \pm 0.05) \times 10^2$ M^{-1} for **G1** bound by **G4⊂1**.

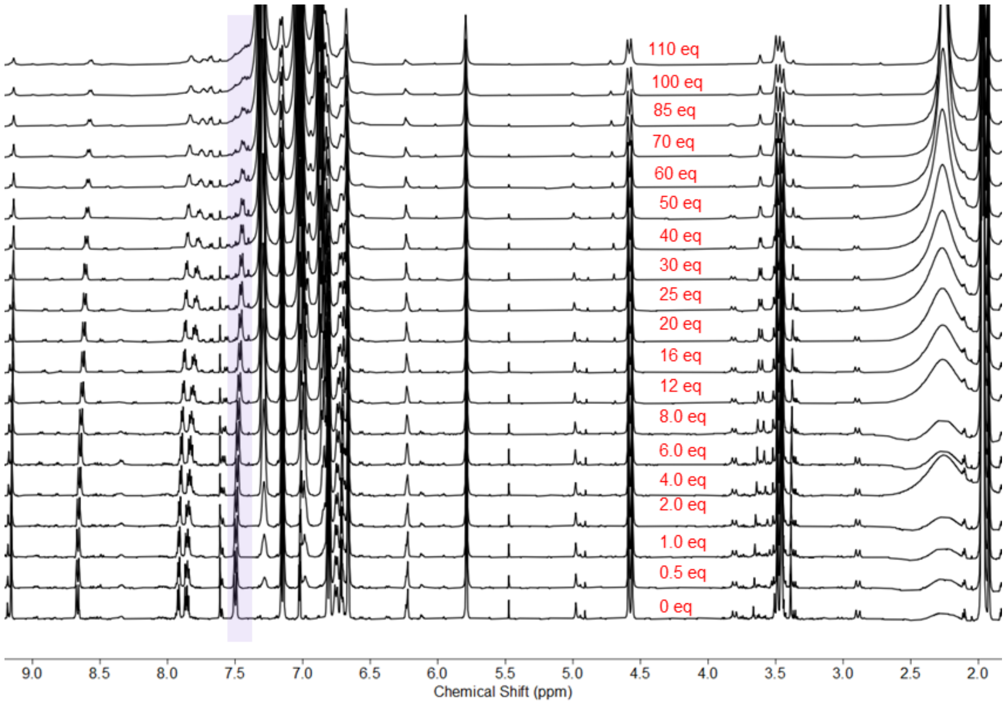

Figure S52. ^{1}H NMR spectra upon addition of **G1** into **G3⊂1** (400 MHz, CD_3CN, 25 °C).

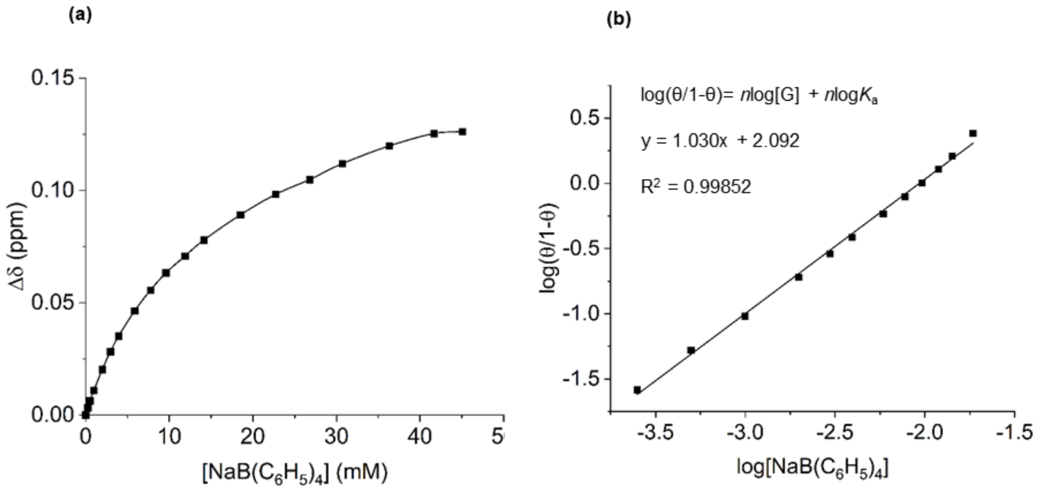

Figure S53. Titration Analysis. **(a)** Titration curves of **G1** bound by **G3⊂1**. **(b)** Hill function.

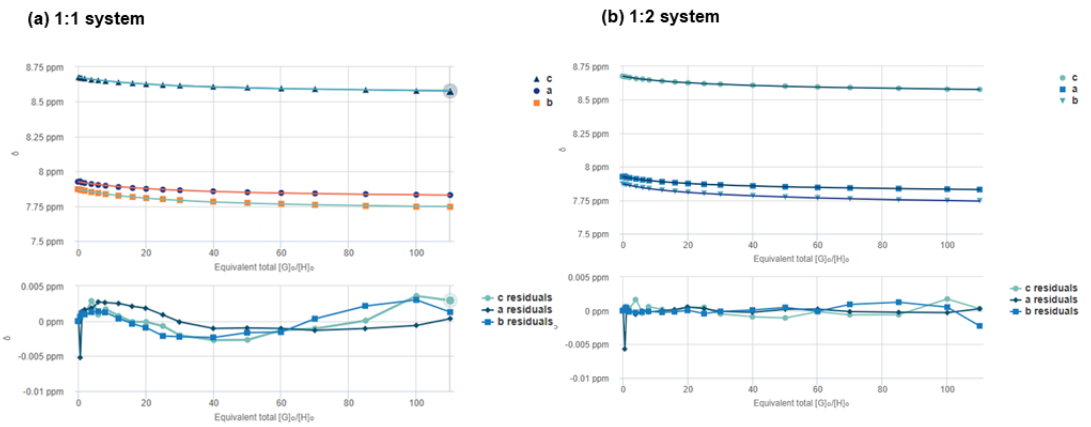

Figure S54. Binding isotherms and residual plots of binding **G1** by **G3⊂1** using BindFit.[5] **(a)** 1:1 system, K_a = 71 ± 2 M^{-1}. **(b)** 1:2 system, K_{11} = 159 ± 5 M^{-1}, K_{12} = 17 ± 1 M^{-1}. Titration data fitted the 1:2 systems with lower and more random residuals, but the exact binding stoichiometry could not be gauged unambiguously; a higher binding stoichiometry could also not be ruled out.

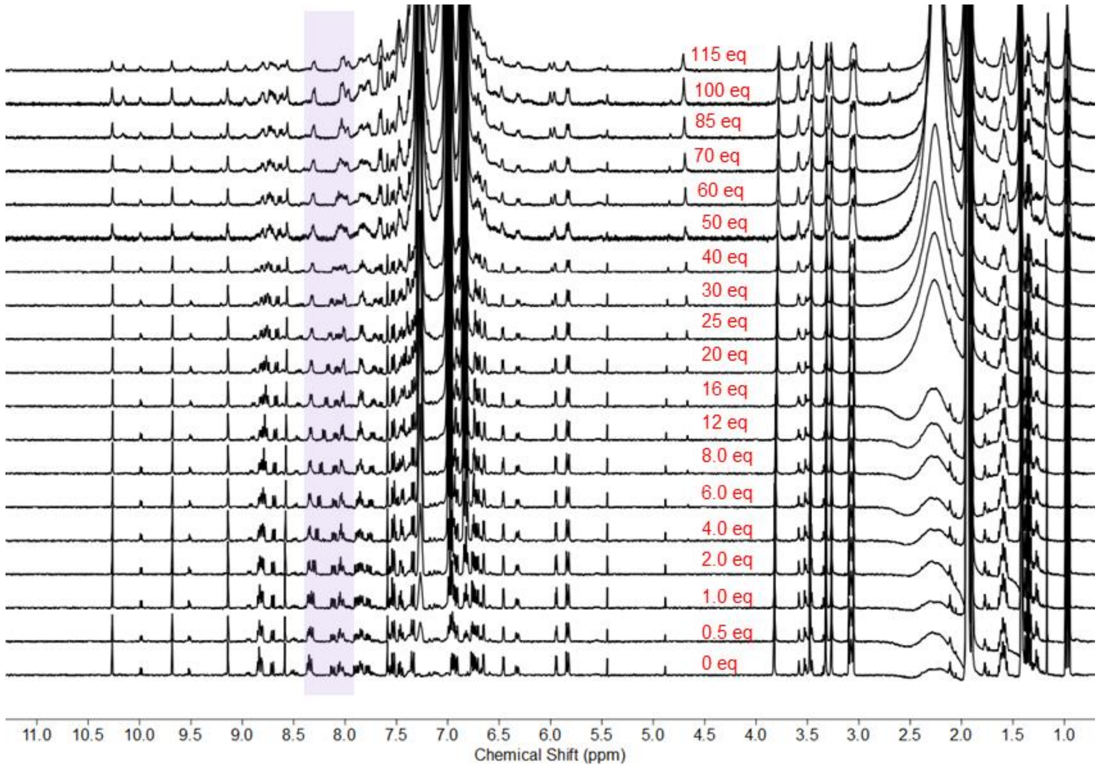

Figure S55. ^{1}H NMR spectra upon addition of **G1** into **G4⊂1** (400 MHz, CD_3CN, 25 °C).

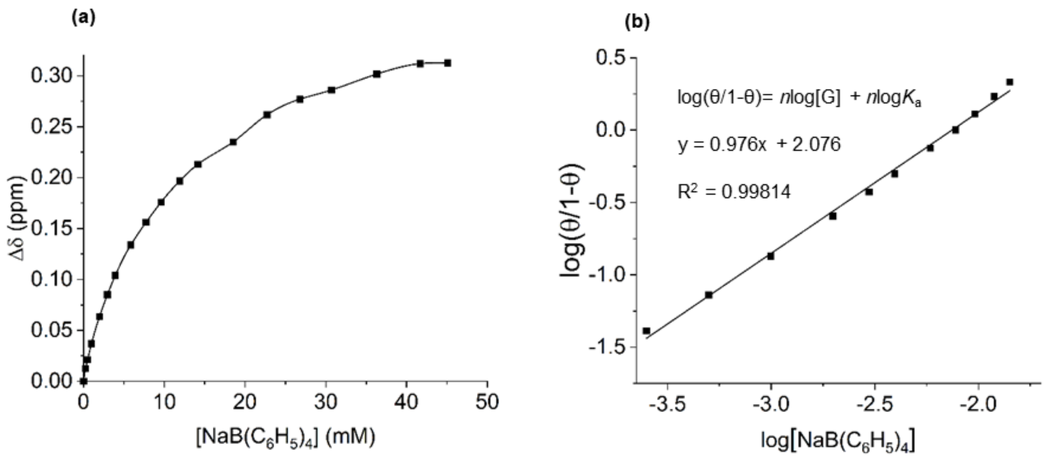

Figure S56 Titration Analysis. **(a)** Titration curves of **G1** bound by **G4⊂1**. **(b)** Hill function.

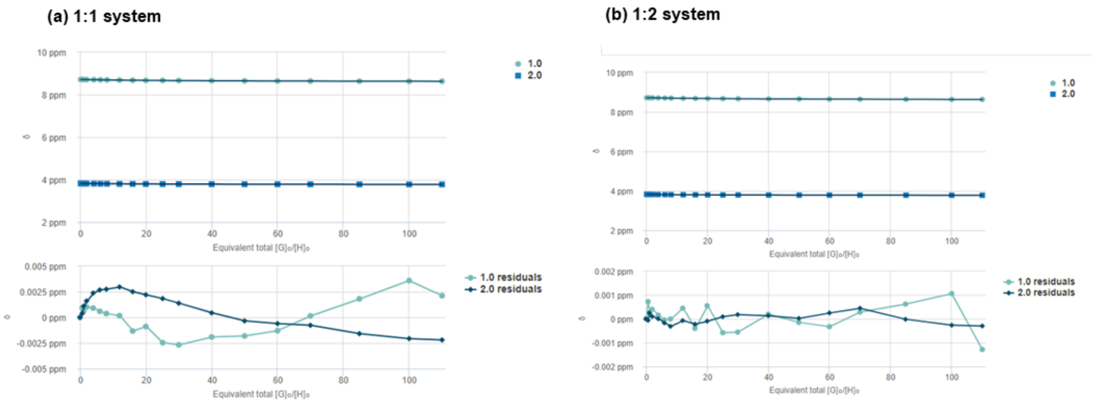

Figure S57. Binding isotherms and residual plots of binding **G1** by **G4⊂1** using BindFit.[5] **(a)** 1:1 system, K_a = 63 ± 6 M^{-1}. **(b)** 1:2 system, K_{11} = 181 ± 6 M^{-1}, K_{12} = 19 ± 1 M^{-1}. Titration data fitted the 1:2 systems with lower and more random residuals, but the exact binding stoichiometry therefore could not be gauged unambiguously; a higher binding stoichiometry could also not be ruled out.

5.5 Host-Guest Interaction of $(\mathbf{G1})_x{\subset}\mathbf{1}$ with G3 and G4

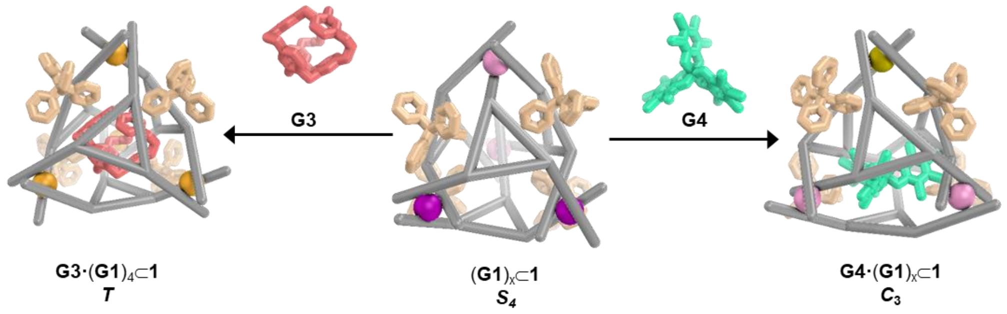

Titrations of G3 bound by (G1)$_x$⊂1: Host-guest complex (**G1**)$_x$⊂**1** was prepared in CD_3CN (0.75 mM) in presence of **G1** (6.0 equiv). To a solution of (**G1**)$_x$⊂**1** in 0.5 mL CD_3CN (0.75 mM) was added **G3** portionwise, using *tert*-butyl acetate as internal standard (0.75 mM). After each addition, the reaction mixture was heated at 70 °C for 2 h. ^{1}H NMR data were collected after cooling down the host-guest complex.

Titrations of G4 bound by (G1)$_x$⊂1: Host-guest complex (**G1**)$_x$⊂**1** was prepared in CD_3CN (0.75 mM) in presence of **G1** (6.0 equiv). To a solution of (**G1**)$_x$⊂**1** in 0.5 mL CD_3CN (0.75 mM) was added **G4** portionwise, using *tert*-butyl acetate as internal standard (0.75 mM). After each addition, the reaction mixture was heated at 70 °C for 2 h. ^{1}H NMR data were collected after cooling down the host-guest complex.

Binding constant for **G3** bound by (**G1**)$_x$⊂**1** was determined to be $K_a = (1.39 \pm 0.10) \times 10^3\ M^{-1}$, using the equation described in section 5.2. Binding constant for **G4** bound by (**G1**)$_x$⊂**1** was determined to be $K_a = (9.48 \pm 0.15) \times 10^4\ M^{-1}$, using the equation described in section 5.3. During titrations, decomposition of cage framework was observed in presence of two guests upon heating.

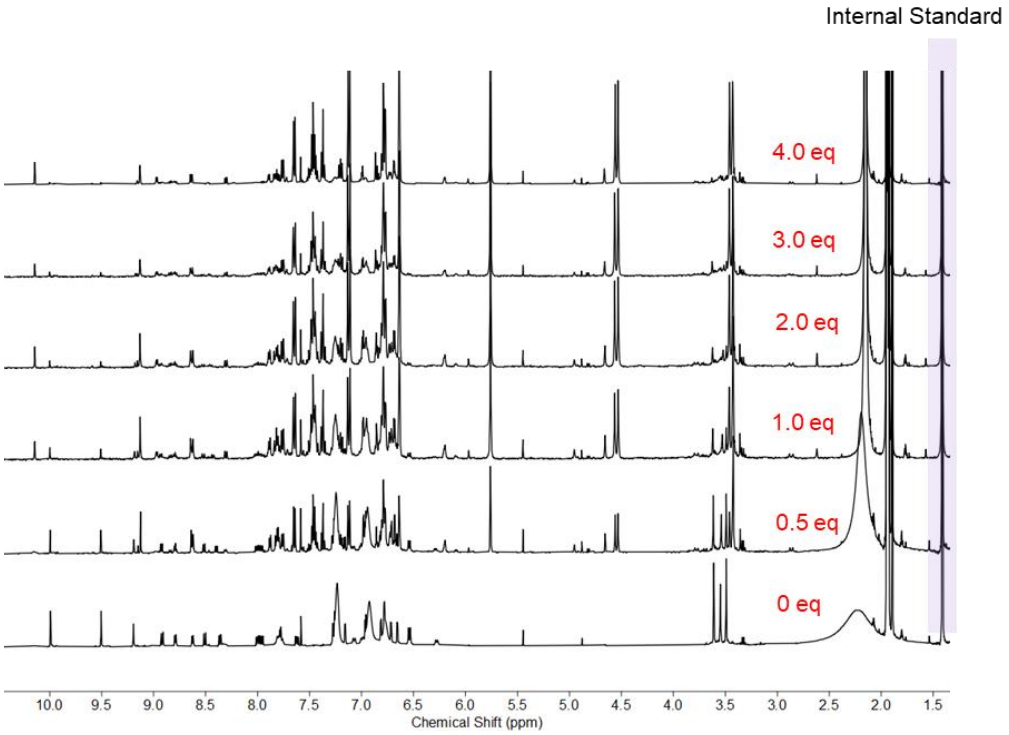

Figure S58. ^{1}H NMR spectra upon addition of **G3** into **(G1)$_x$⊂1** (500 MHz, CD_3CN, 25 °C).

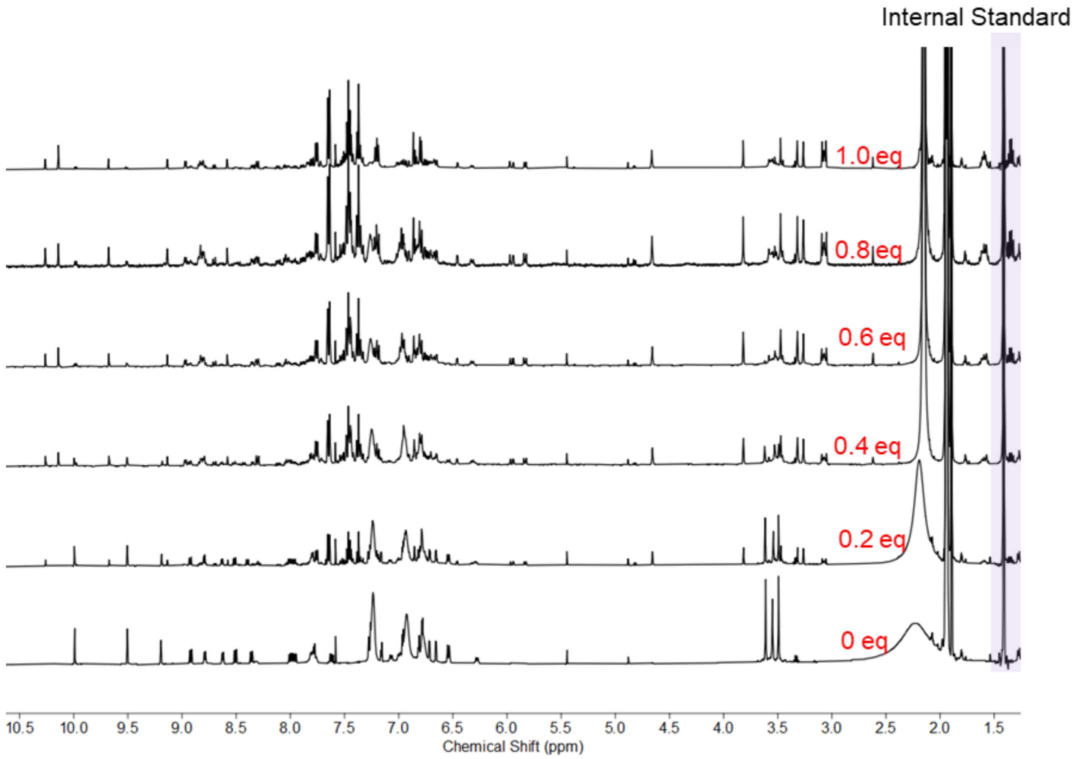

Figure S59. ^{1}H NMR spectra upon addition of **G4** into **(G1)$_x$⊂1** (500 MHz, CD_3CN, 25 °C).

5.6 Host-Guest Interaction of G3⊂1 and G3·(G1)$_x$⊂1 with G4

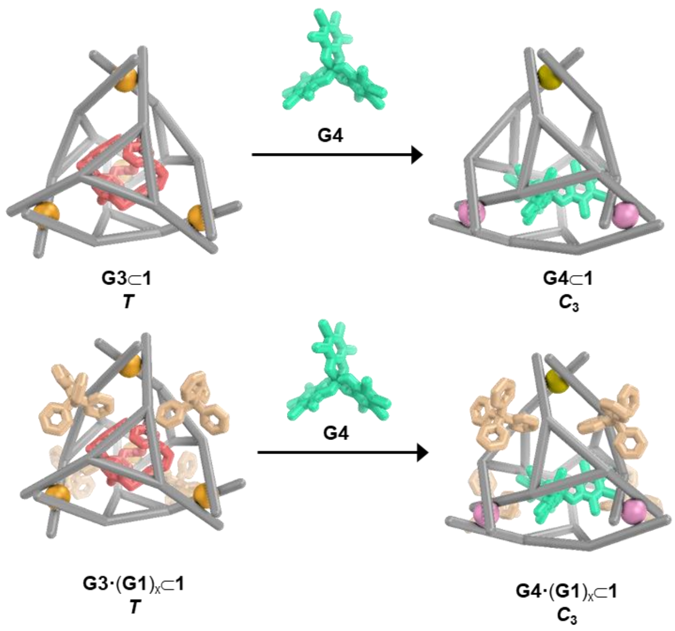

To a solution of **G3⊂1** or **G3·(G1)$_x$⊂1** in 0.5 mL CD_3CN (0.75 mM) was added **G4** (1.5 equiv). The reaction mixture was heated at 70 °C for 2 h. ^{1}H NMR data was collected after cooling down the host-guest complex.

The higher binding affinity of **G4** over **G3** allowed for the guest displacement, thus enabling the conversion of *T*-symmetric species into C_3-symmetric species.

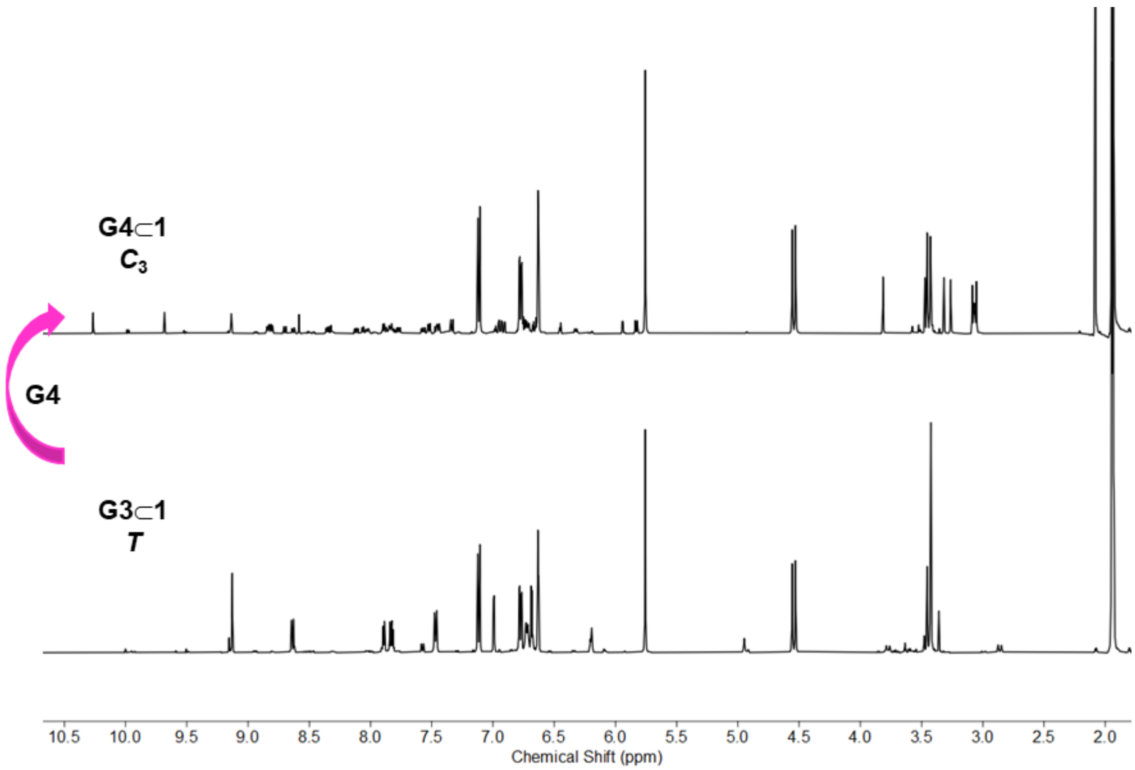

Figure S60. ^{1}H NMR spectra upon addition of **G4** into **G3⊂1** (500 MHz, CD_3CN, 25 °C).

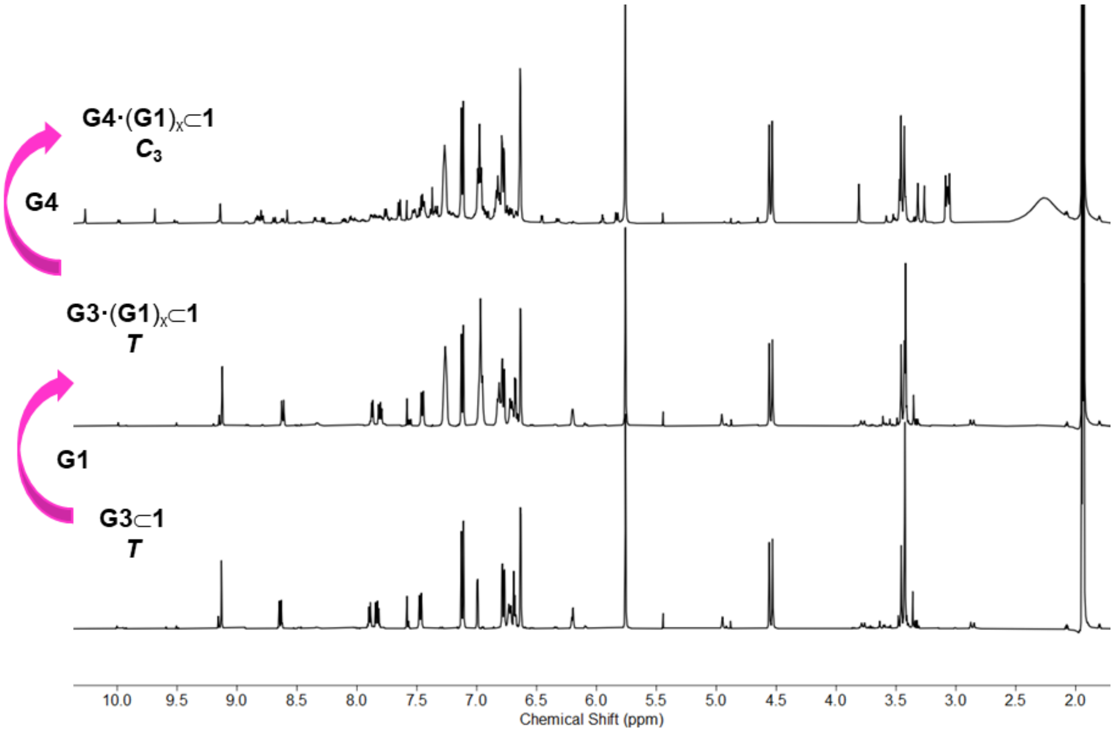

Figure S61. ^{1}H NMR spectra upon addition of **G4** into **G3·(G1)$_x$⊂1** (500 MHz, CD_3CN, 25 °C).

6 Volume Calculations

In order to determine the available void spaces within the structure of **3,** MoloVol[8] calculations based on the crystal structures were performed. A probe with a radius of 2.8 Å was employed. The standard parameters are tabulated below, and the results are shown in Figure S62.

Probe mode: one probe

Probe radius: 2.8 Å

Grid resolution: 0.1 Å

Optimization depth: 4

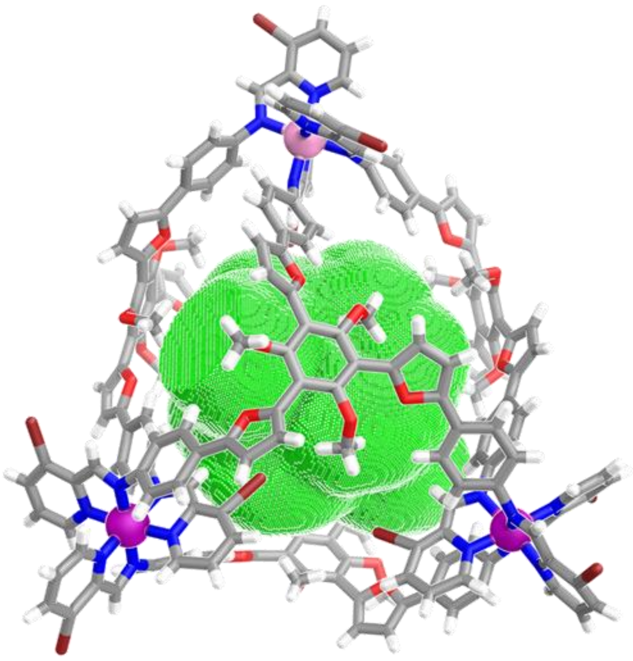

986 Å^3

Figure S62. MoloVol[7]-calculated void space (green mesh) within the crystal structure of **3**.

7 Crystal Structure of 3

Crystals with composition $2[Fe_4L_4]\cdot 8NTf_2+7Et_2O$ were grown by diffusion of ethyl ether into an acetonitrile solution of the cage. Data were collected at Beamline I19 of Diamond Light Source employing silicon double crystal monochromated synchrotron radiation (0.6889 Å) with ω and *ψ* scans at 100(2) K.[9] Data integration and reduction were undertaken with Xia2.[10-11] Subsequent computations were carried out using the WinGX-32 graphical user interface.[12] A multi-scan empirical absorption correction using spherical harmonics was applied to the data using DIALS.[11] The structure was solved by intrinsic phasing using SHELXT[13], and then refined and extended using SHELXL.[14] Carbon-bound hydrogen atoms were included in idealised positions and refined using a riding model. Disorder was modelled using standard crystallographic methods including constraints and restraints where necessary.

The crystals employed immediately lost solvent after removal from the mother liquor. However rapid handling prior to flash cooling in liquid nitrogen and the use of synchrotron radiation enabled the collection of high-resolution data. These measures and the use of synchrotron radiation few reflections at greater than 1.1 Å resolution were observed and the data were trimmed accordingly. Nevertheless, the quality of the data is far more than sufficient to establish the connectivity of the structure. The asymmetric unit was found to contain two halves of two Fe_4L_4 structures, associated counterions and solvent molecules. Thermal parameter restraints (SIMU, RIGU) were applied to all atoms except for Fe.

Due to the limited resolution of the data, the GRADE program[15] was employed using the GRADE Web Server[16] to generate a full set of bond distance and angle restraints (DFIX, DANG, FLAT) for the organic parts of the structure. Thermal parameter restraints (SIMU, RIGU) were applied to all atoms except for Fe and S to facilitate a stable anisotropic refinement. Even with these restraints, some thermal parameters remain larger than ideal as a consequence of the high level of thermal motion throughout the structure, especially for the anions.

The anions within the structure also show evidence of substantial disorder. One NTf_2^- anion was modelled as disordered over two locations, and the occupancies of all located anions were freely refined. Some additional minor occupancy positions of the anions could not be located in the electron density map and were not included in the model.

Consequently, the SQUEEZE[17] function of PLATON[18] was employed to remove the contribution of the electron density associated with the remaining anions and further highly disordered solvent, which gave a potential solvent accessible void of 6523 Å^3 per unit cell (a total of approximately 1538 electrons). Diffuse solvent molecules could not be assigned to MeCN or diethyl ether and were therefore not included in the formula. Consequently, the molecular weight and density given above are underestimated.

Crystallographic data have been deposited with the CCDC (2235215).

We also attempted to obtain the crystal structures of *T*-symmetric host-guest complex **G3**$\subset$**1** and C_3-symmetric host-guest complex **G4**$\subset$**1**. Various conditions (>200 conditions for each) were screened, including solvents, temperatures, concentrations, and counter anions. However, these attempts to obtain single crystals suitable for X-ray diffraction analysis were unsuccessful.

Table S1. Crystal Data and Structure Refinement for Cage 3.

CCDC number	2235215
Empirical formula	$C_{532}H_{406}Br_{24}F_{96}Fe_{8}N_{72}O_{119}S_{32}$
Formula weight	14925.82
Temperature [K]	100(2)
Crystal system	monoclinic
Space group (number)	$P2/c$ (13)
a [Å]	38.8028(3)
b [Å]	24.9335(2)
c [Å]	35.8662(3)
α [°]	90
β [°]	95.6700(10)
γ [°]	90
Volume [Å^3]	34530.4(5)
Z	2
ρ_{calc} [gcm^{-3}]	1.436
μ [mm^{-1}]	1.747
F(000)	14956
Crystal size [mm^3]	0.100×0.100×0.100
Crystal colour	purple
Crystal shape	block
Radiation	(λ=0.6889 Å)

2ϴ range [°]	1.02 to 36.50 (1.10 Å)
Index ranges	-35 ≤ h ≤ 35
	-22 ≤ k ≤ 22
	-32 ≤ l ≤ 32
Reflections collected	137627
Independent reflections	27151<br>R_{int} = 0.0659<br>R_{sigma} = 0.0917
Completeness to<br>ϴ = 18.248°	99.8 %
Data / Restraints / Parameters	27151/7318/4124
Goodness-of-fit on F^2	1.295
Final *R* indexes<br>[$I \geq 2\sigma(I)$]	R_1 = 0.1247<br>wR_2 = 0.3538
Final *R* indexes<br>[all data]	R_1 = 0.1602<br>wR_2 = 0.3721
Largest peak/hole [eÅ^{-3}]	1.94/-1.03

8 References

1. Y. Tamura, H. Takezawa, M. Fujita, *J. Am. Chem. Soc.* **2020**, *142*, 5504–5508.

2. T. Buffeteau, D. Pitrat, N. Daugey, N. Calin, M. Jean, N. Vanthuyne, L. Ducasse, F. Wien, T. Brotin, *Phys. Chem. Chem. Phys.* **2017**, *19*, 18303–18310.

3. M. P. Sibi, G. Petrovic, *Tetrahedron Asymmetry* **2003**, *14*, 2879–2882.

4. S. Mecozzi, J. Rebek, Julius, C*hem. Eur. J.* **1998**, *4*, 1016–1022.

5. a) P. Thordarson, *Chem. Soc. Rev.* **2011**, *40*, 1305–1323; b) H. Takezawa, T. Murase, G. Resnati, P. Metrangolo, M. Fujita, *J. Am. Chem. Soc*. **2014**, *136*, 1786−1788; c) D. Brynn Hibbert, P. Thordarson, *Chem. Commun.* **2016**, *52*, 12792−12805.

6. a) S. E. Howson, L. E. N. Allan, N. P. Chmel, G. J. Clarkson, R. J. Deeth, A. D. Faulkner, D. H. Simpson, P. Scott, *Dalton Trans.* **2011**, *40*, 10416–10433; b) J. M. Dragna, G. Pescitelli, L. Tran, V. M. Lynch, E. V. Anslyn, L. Di Bari, *J. Am. Chem. Soc.* **2012**, *134*, 4398−4407.

7. M. C. Young, L. R. Holloway, A. M. Johnson, R. J. Hooley, *Angew. Chem. Int. Ed.* **2014**, *53*, 9832–9836.

8. J. B. Maglic, R. Lavendomme, *J. Appl. Cryst.* **2022**, *55*, 1033–1044.

9. D. Allan, H. Nowell, S. Barnett, M. Warren, A. Wilcox, J. Christensen, L. Saunders, A. Peach, M. Hooper, L. Zaja, S. Patel, L. Cahill, R. Marshall, S. Trimnell, A. Foster, T. Bates, S. Lay, M. Williams, P. Hathaway, G. Winter, M. Gerstel, R. Wooley, *Crystals* **2017**, *7*, 336.

10. G. Winter, *J. Appl. Crystallogr.* **2010**, *43*, 186–190.

11. G. Winter, D. G. Waterman, J. M. Parkhurst, A. S. Brewster, R. J. Gildea, M. Gerstel, L. Fuentes-Montero, M. Vollmar, T. Michels-Clark, I. D. Young, N. K. Sauter, G. Evans, *Acta Cryst.* **2018**, *D74*, 85–97.

12. L. Farrugia, *J. Appl. Crystallogr.* **2012**, *45*, 849–854.

13. G. M. Sheldrick, *Acta. Cryst.* **2015**, *A71*, 3–8.

14. G. M. Sheldrick, *Acta. Cryst.* **2015**, *C71*, 3–8.

15. G. Bricogne, E. Blanc, M. Brandle, C. Flensburg, P. Keller, W. Paciorek, P. Roversi, A. Sharff, O. S. Smart, C. Vonrhein, T. O. Womack, *BUSTER*, 2.11.2 ed., Global Phasing Ltd., Cambridge, United Kingdom, **2011**.

16. O. S. Smart, T. O. Womack, *Grade Web Server*, Global Phasing Ltd., **2014**.

17. P. van der Sluis, A. L. Spek, *Acta Cryst.* **1990**, *A46*, 194–201.

18. A. L. Spek, *PLATON: A Multipurpose Crystallographic Tool*, Utrecht University, Utrecht, The Netherlands, **2008**.
